# Vegetarian and vegan diets and the risk of cardiovascular disease, ischemic heart disease and stroke: a systematic review and meta-analysis of prospective cohort studies

**DOI:** 10.1007/s00394-022-02942-8

**Published:** 2022-08-27

**Authors:** Jarle Sæby Dybvik, Mette Svendsen, Dagfinn Aune

**Affiliations:** 1 Institute of Clinical Medicine, Faculty of Medicine, University of Oslo, Oslo, Norway; 2 Department of Nutrition Sciences, Institute of Basic Medical Sciences, University of Oslo, Oslo, Norway; 3 Department of Endocrinology, Morbid Obesity and Preventive Medicine, Oslo University Hospital, Oslo, Norway; 4 Department of Epidemiology and Biostatistics, School of Public Health, Imperial College London, London, UK; 5 Department of Nutrition, Oslo New University College, Oslo, Norway; 6 Unit of Cardiovascular and Nutritional Epidemiology, Institute of Environmental Medicine, Karolinska Institutet, Stockholm, Sweden

**Keywords:** Vegetarian, Vegan, Cardiovascular disease, Ischemic heart disease, Stroke, Meta-analysis

## Abstract

**Purpose:**

Vegetarian diets have been associated with reduced risk of ischemic heart disease (IHD). However, results regarding cardiovascular disease (CVD) overall and stroke are less clear. We conducted a systematic review and meta-analysis of prospective cohort studies on CVD, IHD and stroke risk among vegetarians or vegans versus nonvegetarians to clarify these associations.

**Methods:**

PubMed and Ovid Embase databases were searched through August 12, 2021. Prospective cohort studies reporting adjusted relative risk (RR) estimates and 95% confidence intervals (CIs) for incidence or mortality from CVD, IHD and stroke, comparing vegetarians and vegans to nonvegetarians were included. Risk of bias (RoB) was assessed using ROBINS-I and the strength of evidence was assessed using World Cancer Research Fund (WCRF) criteria. Summary RRs (95% CIs) were estimated using a random effects model.

**Results:**

Thirteen cohort studies (844,175 participants, 115,392 CVD, 30,377 IHD, and 14,419 stroke cases) were included. The summary RR for vegetarians vs. nonvegetarians was 0.85 (95% CI: 0.79–0.92, *I*^2^ = 68%, *n* = 8) for CVD, 0.79 (95% CI: 0.71–0.88, *I*^2^ = 67%, *n* = 8) for IHD, 0.90 (95% CI: 0.77–1.05, *I*^2^ = 61%, *n* = 12) for total stroke, and for vegans vs. nonvegetarians was 0.82 (95% CI: 0.68–1.00, *I*^2^ = 0%, *n* = 6) for IHD. RoB was moderate (*n* = 8) to serious (*n* = 5). The associations between vegetarian diets and CVD and IHD were considered probably causal using WCRF criteria.

**Conclusions:**

Vegetarian diets are associated with reduced risk of CVD and IHD, but not stroke, but further studies are needed on stroke. These findings should be considered in dietary guidelines.

**Review registration:**

No review protocol registered.

**Supplementary Information:**

The online version contains supplementary material available at 10.1007/s00394-022-02942-8.

## Introduction

Cardiovascular disease (CVD), which is mainly due to ischemic heart disease (IHD) and stroke [1], is still the leading cause of death and disability globally [2], in spite of decreasing trends in CVD rates in recent decades [2]. IHD and stroke accounted for 9 and 6.6 million deaths in 2019, respectively, according to data from the Global Burden of Disease Study [2]. While suboptimal diets account for at least 46% of all CVD deaths in high- and middle-income countries [3] and dietary risks have been recognized as the second most impactful CVD target [2], plant-based diets have been recommended in several guidelines for preventing CVD [4–7].

There is a continued need for public health interventions to further reduce CVD risk through changes in diet and other lifestyle habits like smoking and physical activity [5]. Plant-based diets are effective for improving CVD risk factors [8, 9]. This is further supported by the favourable cardiometabolic profile seen among vegetarians who predominantly exclude meat, fish and poultry from their diet, when compared to people consuming meat [10–12]. This includes lower prevalence of hypertension [12–14], high serum cholesterol [15], and type 2 diabetes mellitus [16]. Studies also report less overweight and obesity in vegetarians [17, 18], a finding consistent with the lower body mass index (BMI) observed among vegetarians [16, 19].

Vegetarian diets have been consistently shown to reduce the risk of IHD in prospective cohort studies. A pooled analysis of five cohort studies by Key et al. found a 24% reduction in the relative risk of IHD mortality among vegetarians vs. nonvegetarians [20]. Subsequently published studies have also reported associations in the direction of reduced risk [21–23], although not all studies reported clear associations [21, 22]. In contrast, results on risk of stroke or cerebrovascular disease have been less consistent with null results reported in a pooled analysis [20], the UK Biobank [22], and the Nurses’ Health Study 1 and 2 and Health Professionals Follow-up Study [24], but a positive association was reported in the EPIC-Oxford cohort [23] and inverse associations were reported in two Taiwanese studies [25]. Studies on CVD overall have also shown mixed results, with some studies showing inverse associations [20, 22, 23] and others reporting no clear associations [26, 27]. Therefore, to clarify these findings, we conducted a systematic review and meta-analysis of prospective cohort studies on vegetarian or vegan diets and risk of CVD, IHD and stroke.

## Methods

### Study design

We conducted a systematic review and meta-analysis of prospective cohort studies on the association between vegetarian or vegan diets and risk of incidence and mortality from CVD, IHD and stroke, both overall and subtypes. The PRISMA (Preferred Reporting Items for Systematic Reviews and Meta-Analyses) 2020 guideline was followed throughout the process [28]. The PRISMA checklist (both main and for abstract) is available in *Online Resource 1 (Supplementary Tables 1 and 2).*

### Eligibility

The review question was framed with the PECO(S) elements [29] as recommended [30]. Of interest were presumable healthy individuals in the general population, with no restrictions regarding age, sex (men and women) or pregnancy status (P), who adhered to vegetarian or vegan diets (E) and were compared to nonvegetarian subjects (C). Vegetarian diets were defined as diets excluding meat, poultry and seafood, regardless of whether they allowed dairy products (lacto-) or eggs (ovo-) or both (lacto-ovo-vegetarian), and vegan diets as diets excluding all animal products, e.g., dairy products and eggs. Nonvegetarian diets were defined as diets allowing consumption of all types of animal foods, e.g. meat, poultry, seafood, dairy products and eggs. Incidence or mortality from CVD, IHD and stroke (overall and subtypes) were the outcomes of interest (O), and we focused entirely on reports from prospective cohort studies (S).

We applied the following exclusion criteria: (1) duplicate citations, (2) studies reporting unadjusted risk estimates, (3) studies on patient groups, (4) non-relevant exposures like other plant-based nonvegetarian dietary patterns or diet scores (e.g., Mediterranean diet, Dietary Approaches to Stop Hypertension – DASH diet, Plant-based Dietary Index) and (5) non-relevant outcomes (e.g., cardiovascular risk factors) and (6) not relevant study design (e.g., intervention studies, case-control studies, cross-sectional studies, reviews and meta-analyses).

### Search strategy

The search was conducted by one author (JSD) using PubMed and Ovid Embase databases from their inception in 1958 and 1947 to February 14, 2020, and was later updated on August 12, 2021, using the search strategy shown in *Supplementary Table 3 (Online Resource 2)*. We searched for prospective cohort studies reporting on the association between vegetarian or vegan diets and incidence or mortality from CVD, IHD or stroke. Terms like vegetarian(s) or vegan(s) or vegetarian/vegan diet were searched in combination with cardiovascular disease, ischemic heart disease, coronary heart disease, myocardial infarction, cerebrovascular disease, stroke, cerebral hemorrhage, and subarachnoid hemorrhage. We added terms for ‘Seventh-day Adventists’ as many of the relevant cohort studies have been done in this religious group. ‘Subject headings’ were used for PubMed (MeSHs) and Ovid Embase (Emtrees) databases, and text words were applied to retrieve any articles in press. No restrictions were used for, e.g. age, sex, geographic location, language or date. We also screened the reference lists of relevant cohort studies and reviews to check for papers not contained within our search.

### Screening and study selection

All references were imported into EndNote X9 and initially screened by JSD, while the second part of the screening (studies deemed potentially relevant based on abstract/title) was performed in duplicate by JSD and DA. Any disagreements were resolved through discussion. We included prospective cohort studies if they reported adjusted relative risk (RR) estimates (including hazard ratios [HRs] and incidence or death rate ratios) with 95% confidence intervals (95% CIs) for the association between vegetarian or vegan vs. nonvegetarian diets and incidence and/or mortality from CVD, IHD or stroke. When study data had been published on more than one occasion, we included the report with the largest number of cases. Prospective cohort studies were deemed the most relevant study design, as there are to our knowledge no randomized controlled trials available on vegetarian diets and primary prevention of CVD. Retrospective case-control studies were not included since they can be affected by recall bias and selection bias and cross-sectional studies were excluded because of a lack of temporal relation between the exposure and the outcome. A complete list of citations excluded after full-text assessment is shown in *Supplementary Table 4 (Online Resource 3)*.

### Data extraction

Data regarding results and study characteristics were extracted to tables by one author (JSD) and checked for accuracy by a second author (DA). More specifically, the information extracted was as follows: author, year of publication, location by country, study name, study period and years of follow-up, age and sex, sample size, number of cases or deaths from CVD, IHD, total stroke, ischemic stroke and hemorrhagic stroke, and type of diet. We also extracted RRs and 95% CIs and information regarding confounders adjusted for in each study´s statistical analysis.

### Risk of bias assessment

All studies were critically appraised by two authors (JSD, DA) and discrepancies resolved through consensus with a third author (MS) using the Cochrane ROBINS-I (Risk Of Bias In Non-randomised Studies—of Interventions) tool [31] as this is recommended for a more adequate and qualitative assessment of internal validity in studies [30]. However, we did modifications to the tool in accordance with a similarly adapted version [32, 33] to better assess risk of bias (RoB) in exposure studies [34], but otherwise, we followed the detailed guidance for ROBINS-I [35]. With the ROBINS tool, each study was measured against a hypothetical target randomized trial in a way that deviations from such a target trial was considered bias. We assessed seven bias domains, including confounding, selection of participants, classification of diet groups, departures from baseline diet groups, missing data, measurement of outcomes and selection of the reported results by answering signalling questions and critically mirroring each domain against a set of prespecified criteria (*Supplementary Table 5, Online Resource 4*). Each domain could be judged as ‘low’, ‘moderate’, ‘serious’ or ‘critical’ RoB or ‘no information’ on which to base a judgement. An overall RoB judgement was then assigned to each study (study-level assessment) using another set of criteria (*Supplementary Table 6*, Online Resource 4). In agreement with ROBINS, the most severe RoB judgement that was assigned to a given domain was assigned as the overall RoB for a study.

### Evidence grading

The grading of the evidence was done initially by DA and then discussed between all the authors to reach a conclusion. We used the World Cancer Research Fund (WCRF) grading criteria (*Supplementary Table 7*, Online Resource 4) for grading the overall evidence regarding vegetarian diets and CVD, IHD and stroke, with possible gradings rated as convincing, probable, limited-suggestive, limited-no conclusion, or substantial effect on risk unlikely [36, 37].

### Statistical methods

We calculated summary RRs and 95% CIs comparing vegetarians and vegans to nonvegetarians in relation to the risk of CVD, IHD and stroke (overall, ischemic and hemorrhagic) using the random-effects model by DerSimonian and Laird [38] to account for any study level variability. A two-tailed p < 0.05 was considered statistically significant. The results of the synthesis were visually displayed as forest plots. For studies reporting both on incidence and mortality from CVD, IHD or stroke, only risk estimates for incidence were used in the main analysis. When studies reported on both risk of IHD and stroke/cerebrovascular disease separately but not for CVD overall, we pooled results for the two outcomes using a fixed effects model before inclusion in the main analysis of CVD (Adventist Mortality Study, Adventist Health Study 1, EPIC-Oxford) [20, 23]. Since the vast majority of cerebrovascular disease deaths are due to stroke, we included studies reporting on cerebrovascular disease mortality together with studies on stroke. Studies reporting risk estimates for circulatory disease were included in the analysis of CVD.

Heterogeneity was assessed using Cochran’s Q-statistics, Chi-squared-statistics [39] and I^2^-statistics [40]. We considered the *p* value for Chi-squared statistics significant if < 0.05, and *I*^2^-percentage ranged from 0-100%. Subgroup analyses were conducted stratified by sex (men or women), years of follow-up (≥ 10 vs. < 10 years), exclusion of early follow-up years or not, use of incidence or mortality estimates, number of cases or deaths (< 250, 250-499 and ≥ 500), geographic location (Europe, North America or Asia), risk of bias (low, moderate, serious), and adjustment for confounding factors (age, education, smoking, alcohol, BMI, physical activity) to investigate potential sources of heterogeneity in the main analysis. For CVD, we also conducted a separate subgroup analysis distinguishing between CVD and circulatory disease. For stroke, we added separate subgroup analyses distinguishing between total stroke and cerebrovascular disease, and between ischemic and hemorrhagic stroke. An additional sensitivity analysis was conducted only on studies reporting both BMI adjusted and BMI unadjusted risk estimates to assess more directly the impact of adjustment for BMI on the results, since BMI could be an intermediary variable through which vegetarian or vegan diets can influence cardiovascular risk. We added a similar sensitivity analysis restricted to studies reporting results with both inclusion and exclusion of early follow-up to assess more directly the impact of excluding early follow-up, since reverse causality could potentially bias the results. Meta-regression analyses were conducted to test for heterogeneity between subgroups. We conducted a sensitivity analysis using more adjusted estimates from a pooled analysis by Key et al. [20] (also adjusted for education, alcohol, physical activity and BMI in addition to age, sex and smoking) in the analysis of IHD, replacing the overlapping individual studies that were included in the primary analysis with the pooled estimate to assess the impact of more rigorous adjustment on the overall conclusion.

Egger´s test [41], Begg´s test [42], and inspection of the funnel plots [43] were used to investigate publication bias, and we considered a *p* value < 0.10 or funnel plot asymmetry to indicate possible publication bias. To assess the robustness of the summary RRs and to ensure that the summary RRs were not driven by one very large study or a study with an extreme RR, influence analyses were conducted re-calculating the summary RRs when omitting one study at a time from the analysis. Furthermore, we calculated E-values for the association between vegetarian diets and CVD, IHD and stroke to assess the potential impact of unmeasured or uncontrolled confounding [44]. The E-value is defined as the minimum strength that an unmeasured or uncontrolled confounder would have with both the exposure and the outcome to fully explain away the observed association. The statistical analysis was conducted by JSD using Stata SE version 15 (StataCorp, TX, USA).

## Results

**Fig. 1:**
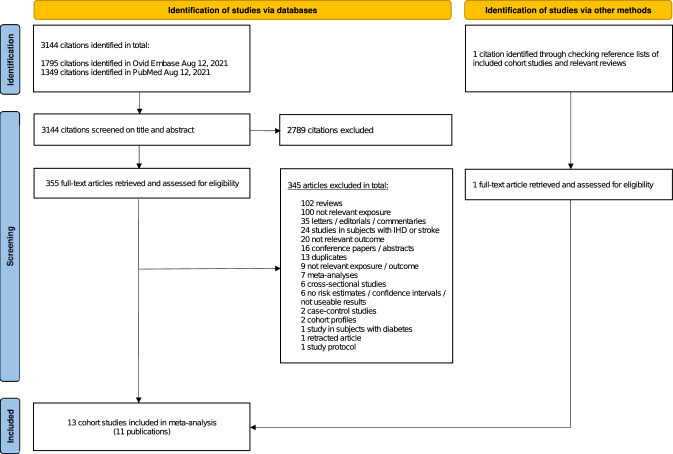
PRISMA flowchart depicting the literature search and the inclusion/exclusion process. From: Page MJ, McKenzie JE, Bossuyt PM, Boutron I, Hoffmann TC, Mulrow CD, et al. (2021) The PRISMA 2020 statement: an updated guideline for reporting systematic reviews. BMJ 372:n71. https://doi.org/10.1136/bmj.n71

Our search yielded a total of 3145 citations (details are shown in the flowchart in Fig. 1), including one citation [26] which was retrieved by checking reference lists of relevant papers. After screening and full-text assessment, 11 publications [20–27, 45–47] with data from 13 unique prospective cohort studies were included in the analysis (Table 1). Two publications reported RRs for two studies each [25, 26] and one publication reported results for three studies [24]. For two studies (Adventist Mortality Study, Adventist Health Study 1), we used data from a pooled re-analysis of Key et al. [20] in the main analysis of vegetarian diets and CVD and IHD, although in the sex-stratified analysis on IHD, we included rather data from the original papers [46, 47] as these contained data for men and women separately. As the original publication from the Heidelberg Study [27] did not contain results on cerebrovascular disease, we also used data from the pooled analysis of Key et al. [20] for our analysis of vegetarian diets and stroke. Results from the pooled analyses [20] were also included in the analyses of vegans and risk of CVD, IHD and total stroke.

**﻿Table 1:** Characteristics of the included cohort publications

Author, year, country [reference]	Study name	Study period, follow-up	Number of participants, sex, age: number of cases/deaths	﻿Exposure vs. comparator, subgroup	Outcome	﻿RR (95% CI)	﻿Adjustment
Snowden, 1984, USA [47]	Adventist Mortality Study (AMS)	1960–1980, 21 years follow-up	22362 M/W, age 30–84 years: 1243 IHD deaths	Vegetarians vs. nonvegetarians, M Vegetarians vs. nonvegetarians, W	IHD mort. IHD mort.	0.67 (0.56–0.77)^a^ 0.73 (0.63–0.83)^a^	Age
Fraser, 1995, USA [46]	Adventist Health Study 1 (AHS-1)	1974–1976 – 1982, 5.14 years follow-up	27321 M/W, age > 24 years: 448 IHD cases	Vegetarians vs. nonvegetarians, M Vegetarians vs. nonvegetarians, W	IHD inc. IHD inc.	0.56 (0.40–0.78)^a^ 1.02 (0.74–1.41)^a^	Smoking, BMI, exercise, diabetes mellitus, hypertension, nut consumption
Key, 1999, USA [20]	Adventist Mortality Study (AMS)	1959–1960 – 1965, 5.6 years follow-up	24538 M/W, age 16–89 years: 780 CVD deaths 598 IHD deaths 182 CBVD deaths	Vegetarians vs. nonvegetarians Vegetarians vs. nonvegetarians Vegetarians vs. nonvegetarians	CVD mort.^b^ IHD mort. CBVD mort.	0.72 (0.62–0.83)^b^ 0.74 (0.63–0.88) 0.65 (0.48–0.87)	Age, sex, and smoking status
Key, 1999, USA [20]	Adventist Health Study 1 (AHS-1)	1976–1980 – 1988, 11.1 years follow-up	28952 M/W, age 16–89 years: 1238 CVD deaths 921 IHD deaths 317 CBVD deaths	Vegetarians vs. nonvegetarians Vegetarians vs. nonvegetarians Vegetarians vs. nonvegetarians	CVD mort.^b^ IHD mort. CBVD mort.	0.70 (0.61–0.80)^b^ 0.62 (0.53–0.73) 0.93 (0.73–1.19)	Age, sex, and smoking status
Key, 1999, Germany [20]	Heidelberg Study (HBS)	1978–1981 – 1989, 9.9 years follow-up	1757 M/W, age 16–89 years: 31 CBVD deaths	Vegetarians vs. nonvegetarians	CBVD mort.	1.69 (0.69–4.15)	Age, sex, and smoking status
Key, 1999, USA, Germany, UK (pooled analysis) [20]	Adventist Mortality Study (AMS) + Adventist Health Study 1 (AHS-1) + Heidelberg Study (HBS) + Oxford Vegetarian Study (OVS)	11.7 years follow-up	76172 M/W, age 16–89 years: 3173 CVD deaths 2264 IHD deaths 909 CBVD deaths	Vegans vs. regular meat-eaters Vegans vs. regular meat-eaters Vegans vs. regular meat-eaters Vegetarians vs. regular meat-eaters Vegetarians vs. regular meat-eaters	CVD mort.^b,c^ IHD mort.^c^ CBVD mort.^c^ IHD mort.^d−^ IHD mort.^d+^	0.73 (0.47–1.14)^b,c^ 0.74 (0.46–1.21)^c^ 0.70 (0.25–1.98)^c^ 0.64 (0.53–0.77)^d−^ 0.66 (0.55–0.79)^d+^	Age, sex, and smoking ^d^Age, sex, smoking status, alcohol use, education, exercise ± BMI
			43048 M/W (detailed confounders available): 1047 IHD deaths				
Appleby, 2002, UK [26]	Oxford Vegetarian Study (OVS)	1980–1984 – 2000, 17.6 years follow-up	11045 M/W, 16–89 years: 469 circulatory disease deaths 375 CVD deaths 250 IHD deaths 125 CBVD deaths	Vegetarians vs. nonvegetarians Vegetarians vs. nonvegetarians Vegetarians vs. nonvegetarians Vegetarians vs. nonvegetarians Vegetarians vs. nonvegetarians Vegetarians vs. nonvegetarians Vegetarians vs. nonvegetarians	CircD mort. CircD mort.^e^ CVD mort.^f^ IHD mort. IHD mort.^e^ CBVD mort. CBVD mort.^e^	0.93 (0.77–1.12) 1.01 (0.82–1.24)^e^ 0.93 (0.75–1.14)^f^ 0.86 (0.67–1.12) 0.98 (0.73–1.30)^e^ 1.08 (0.75–1.54) 1.10 (0.75–1.63)^e^	Age, sex, smoking
Appleby, 2002, UK [26]	Health Food Shoppers Study (HFSS)	1973–1979 – 1997, 18.7 years follow-up	10736 M/W, age 16–89 years: 1117 circulatory disease deaths 892 CVD deaths 562 IHD deaths 330 CBVD deaths	Vegetarians vs. nonvegetarians Vegetarians vs. nonvegetarians Vegetarians vs. nonvegetarians Vegetarians vs. nonvegetarians Vegetarians vs. nonvegetarians Vegetarians vs. nonvegetarians Vegetarians vs. nonvegetarians	CircD mort. CircD mort.^e^ CVD mort.^f^ IHD mort. IHD mort.^e^ CBVD mort. CBVD mort.^e^	0.95 (0.84–1.07) 0.97 (0.85–1.12)^e^ 0.90 (0.78–1.03)^f^ 0.85 (0.71–1.01) 0.89 (0.73–1.09)^e^ 0.99 (0.79–1.24) 0.97 (0.75–1.24)^e^	Age, sex, smoking
Chang-Claude, 2005, Germany [27]	Heidelberg Study (HBS)	1978–1999, 21 years follow-up	1724 M/W, age ≥ 10 years: 219 circulatory disease deaths 60 IHD deaths	Vegetarians vs. nonvegetarians Vegetarians vs. nonvegetarians	CircD mort. IHD mort.	0.83 (0.62–1.12) 0.70 (0.41–1.18)	Age, sex, smoking, physical activity, alcohol, BMI, education
Orlich, 2013, USA [21]	Adventist Health Study 2 (AHS-2)	2002–2007 – 2009, 5.79 years follow-up	73308 M/W, age ≥ 25 years: 987 CVD deaths 372 IHD deaths	Vegetarians vs. nonvegetarians Vegetarians vs. nonvegetarians Vegetarians vs. nonvegetarians, M Vegetarians vs. nonvegetarians, W Vegetarians vs. nonvegetarians, M Vegetarians vs. nonvegetarians, W Vegans vs. nonvegetarians Vegans vs. nonvegetarians	CVD mort. IHD mort. CVD mort. CVD mort. IHD mort. IHD mort. CVD mort. IHD mort.	0.87 (0.75–1.01) 0.81 (0.64–1.02) 0.71 (0.57–0.90) 0.99 (0.83–1.18) 0.71 (0.51–1.00) 0.88 (0.65–1.20) 0.91 (0.71–1.16) 0.90 (0.60–1.33)	Age, sex (all), race, smoking, exercise, income, education, marital status, alcohol, region, sleep, and for women: menopause and hormone therapy
Appleby, 2016, UK pooled analysis [45]	Oxford Vegetarian Study (OVS) + EPIC-Oxford	1980–1984 – 2014 + 1993-1999 – 2014, 16.6 years follow-up	60310 M/W, age 20–89 years: 433 circulatory disease deaths 175 IHD deaths 152 CBVD deaths	Vegetarians vs. regular meat-eaters Vegetarians vs. regular meat-eaters Vegetarians vs. regular meat-eaters	CircD mort.^g^ IHD mort.^g^ CBVD mort.^g^	1.10 (0.95–1.27)^g^ 0.99 (0.79–1.23)^g^ 1.21 (0.94–1.56)^g^	Age, smoking, alcohol, physical activity, married or cohabiting, nutritional supplementation, method of recruitment, sex, parity, oral contraceptive use, hormone replacement therapy use, prior diabetes, prior high blood pressure, receipt of long-term medical treatment
Tong, 2019, UK [23]	EPIC-Oxford	1993–2001 – 2016, 18.1 years follow-up	48188 M/W, age 20–90 years: 3892 CVD cases 2820 IHD cases 1072 stroke cases 519 ischemic stroke cases 300 hemorrhagic stroke cases	Vegetarians vs. meat-eaters Vegetarians vs. meat-eaters Vegetarians vs. meat-eaters Vegetarians vs. meat-eaters Vegetarians vs. meat-eaters Vegetarians vs. meat-eaters Vegetarians vs. meat-eaters Vegetarians vs. meat-eaters Vegetarians vs. meat-eaters Vegetarians vs. meat-eaters Vegetarians vs. meat-eaters, M Vegetarians vs. meat-eaters, W Vegetarians vs. meat-eaters, M Vegetarians vs. meat-eaters, W Vegans vs. meat-eaters Vegans vs. meat-eaters Vegans vs. meat-eaters	CVD inc.^b^ CVD inc.^b,e^ IHD inc. IHD inc.^h^ IHD inc.^e^ Total stroke inc. Total stroke inc.^h^ Total stroke inc.^e^ Ischemic stroke inc. Hemorr. stroke inc. IHD inc. IHD inc. Total stroke inc. Total stroke inc. CVD inc.^b^ IHD inc. Total stroke inc.	0.90 (0.82–0.98)^b^ 0.90 (0.82–0.99)^b,e^ 0.78 (0.70–0.87) 0.83 (0.75–0.92)^h^ 0.80 (0.72–0.90)^e^ 1.20 (1.02–1.40) 1.21 (1.03–1.42)^h^ 1.19 (1.00–1.41)^e^ 1.12 (0.90–1.41) 1.43 (1.08–1.90) 0.77 (0.66–0.91) 0.78 (0.68–0.90) 0.99 (0.73–1.34) 1.28 (1.07–1.53) 0.94 (0.75–1.17)^b^ 0.82 (0.64–1.05) 1.35 (0.95–1.92)	Age, sex (all), method of recruitment, region, year of recruitment, education, Townsend deprivation index, smoking, alcohol, physical activity, dietary supplements, plus oral contraceptive, and hormone replacement therapy use in women ^h^BMI-adjusted model: same adjustments as M/W-model plus BMI
Chiu, 2020, Taiwan [25]	Tzu Chi Health Study (TCHS, cohort 1)	2007–2009 – 2014, 6.1 years follow-up	5050 M/W, age ≥ 20 years: 54 stroke cases 31 ischemic stroke cases	Vegetarians vs. nonvegetarians Vegetarians vs. nonvegetarians	Total stroke inc. Ischemic stroke inc.	0.51 (0.25–1.06) 0.26 (0.08–0.88)	Age, sex, smoking, alcohol, betel nut, physical activity, education, hypertension, diabetes mellitus, dyslipidaemia, IHD and BMI
Chiu, 2020, Taiwan [25]	Tzu Chi Vegetarian Study (TCVS, cohort 2)	2005–2014, 9.25 years follow-up	8302 M/W, age ≥ 20 years: 121 stroke cases 54 ischemic stroke cases 28 hemorrhagic stroke cases	Vegetarians vs. nonvegetarians Vegetarians vs. nonvegetarians Vegetarians vs. nonvegetarians	Total stroke inc. Ischemic stroke inc. Hemorr. stroke inc.	0.52 (0.33–0.82) 0.41 (0.19–0.88) 0.34 (0.12–1.00)	Age, sex, smoking, alcohol, betel nut, physical activity, education, hypertension, diabetes mellitus, dyslipidaemia, IHD
Petermann-Rocha, 2021, UK [22]	UK Biobank (UKB)	2006–2010 – 2020, 8.5 years follow-up	422791 M/W, age 37–73 years: 106690 CVD cases 6580 CVD deaths 24794 IHD cases 2767 IHD deaths 5946 stroke cases 1088 stroke deaths	Vegetarians vs. meat-eaters Vegetarians vs. meat-eaters Vegetarians vs. meat-eaters Vegetarians vs. meat-eaters Vegetarians vs. meat-eaters Vegetarians vs. meat-eaters Vegetarians vs. meat-eaters Vegetarians vs. meat-eaters Vegetarians vs. meat-eaters Vegetarians vs. meat-eaters, M Vegetarians vs. meat-eaters, W Vegetarians vs. meat-eaters, M Vegetarians vs. meat-eaters, W Vegetarians vs. meat-eaters, M Vegetarians vs. meat-eaters, W	CVD inc. CVD inc.^i^ CVD mort. IHD inc. IHD inc.^i^ IHD mort. Total stroke inc. Total stroke inc.^i^ Total stroke mort. CVD inc. CVD inc. IHD inc. IHD inc. Total stroke inc. Total stroke inc.	0.91 (0.86–0.96) 0.84 (0.80–0.89)^i^ 0.91 (0.71–1.17) 0.96 (0.85–1.07) 0.90 (0.80–1.01)^i^ 0.88 (0.59–1.30) 0.84 (0.66–1.07) 0.82 (0.64–1.05)^i^ 0.87 (0.48–1.58) 0.88 (0.81–0.96) 0.93 (0.87–0.99) 0.91 (0.78–1.07) 1.02 (0.87–1.20) 0.81 (0.56–1.18) 0.88 (0.63–1.22)	Age, sex (all), deprivation, ethnicity, morbidity (43 diseases), smoking, sedentary time, alcohol, physical activity, BMI ^i^Model with no BMI-adjustment: same adjustments as BMI-adjusted model except for BMI
Baden, 2021, USA [24]	Nurses’ Health Study 1 (NHS1)	1984–2016, 28.3 years follow-up	73890 W, age 30–55 years: 3604 stroke cases	Vegetarians vs. nonvegetarians, W	Total stroke inc.	1.03 (0.72–1.49)	Age, white race, smoking status, alcohol intake, physical activity, multivitamin use, aspirin use, margarine intake, total energy intake, BMI, postmenopausal hormone use
Baden, 2021, USA [24]	Nurses’ Health Study 2 (NHS2)	1991–2017, 25.5 years follow-up	92352 W, age 25–42 years: 740 stroke cases	Vegetarians vs. nonvegetarians, W	Total stroke inc.	0.71 (0.32–1.59)	Age, white race, smoking status, alcohol intake, physical activity, multivitamin use, aspirin use, margarine intake, total energy intake, BMI, postmenopausal hormone use
Baden, 2021, USA [24]	Health Professionals Follow-up Study (HPFS)	1986–2012, 22.5 years follow-up	43266 M, age 40–75 years: 1897 stroke cases	Vegetarians vs. nonvegetarians, M	Total stroke inc.	0.96 (0.59–1.58)	Age, white race, smoking status, alcohol intake, physical activity, multivitamin use, aspirin use, margarine intake, total energy intake, BMI, postmenopausal hormone use

The relative risk estimates (RRs) compare either vegetarians to nonvegetarians or vegans to nonvegetarians and the main analysis uses incidence and mortality data on both sexes combined, but only incidence data if studies reported both

*AHS-1* Adventist Health Study 1, *AHS-2* Adventist Health Study 2, *AMS* Adventist Mortality Study,* CBVD* cerebrovascular disease, *CI* confidence interval,* CircD* circulatory disease, *CVD* cardiovascular disease, *EPIC-Oxford* European Prospective Investigation into Cancer and Nutrition – Oxford, *HBS* Heidelberg Study,* hemorr.* hemorrhagic, *HFSS* Health Food Shoppers Study, *HPFS* Health Professionals Follow-up Study, *IHD* ischemic heart disease,* inc.* incidence, *M* men,* mort.* mortality, *NHS1* Nurses’ Health Study 1, *NHS2* hemorr. hemorrhagic, Nurses’ Health Study 2, *OVS* Oxford Vegetarian Study, *RR* relative risk, *TCHS* Tzu Chi Health Study, *TCVS* Tzu Chi Vegetarian Study, *UK* United Kingdom, *UKB* UK Biobank Study, *USA* United States of America, *W* women

^a^The data (RRs and 95% CIs) were calculated using the inverse method as original data compared nonvegetarians to vegetarians

^b^Data on IHD and total stroke were pooled into RRs for CVD for these publications: Key 1999 (AMS, AHS-1) and Tong 2019 (EPIC-Oxford)

^c^The risk estimate is a pooled RR and was only used in the analysis on vegan diets and the risk of CVD, IHD and total stroke

^d^^−/d+^The risk estimate is a pooled RR and was only used in a subgroup analysis on BMI-adjustment for vegetarian diets and IHD. Minus (-) indicates no BMI-adjustment. Pluss ( +) indicates BMI-adjustment

^e^Model with RR for exclusion of early follow-up: Appleby 2002 (HFSS, OVS) excluded the first 5 years of follow-up, Tong 2019 (EPIC-Oxford) excluded participants with less than 5 years of follow-up, and Petermann-Rocha 2021 (UKB) excluded the first two years of follow-up

^f^Data on IHD and CBVD were pooled into RRs for CVD for one publication (Appleby 2002-HFSS, OVS)

^g^The risk estimate is for EPIC-Oxford and OVS combined and was only used in subgroup analysis of vegetarian diets and the risk of mortality from CVD, IHD and total stroke

^h^Model with BMI adjustment

^i^Model without BMI-adjustment

### Study characteristics

Table 1 shows a summary of the study characteristics and RRs of the cohort studies included in our main and subgroup analyses. The included studies were as follows: Adventist Mortality Study [20], Adventist Health Study 1 [20], Adventist Health Study 2 [21], Nurses' Health Study 1 [24], Nurses' Health Study 2 [24], Health Professionals Follow-up Study [24] , EPIC-Oxford [23], Health Food Shoppers Study [26], Oxford Vegetarian Study [26], UK Biobank [22], Heidelberg Study [27], Tzu Chi Health Study [25], Tzu Chi Vegetarian Study [25]. Six studies were from North America (USA), five from Europe (United Kingdom and Germany) and two from Asia (Taiwan) (Table 1) and participants were regarded as health-conscious people, both vegetarians and nonvegetarians.

All studies used food frequency questionnaires (FFQs) [20–27] to ascertain vegetarian status, except one study (Health Food Shoppers Study) [26] that relied exclusively on self-identification of vegetarian status without further elaboration on frequency of consumption of meat, fish or poultry. A total of seven studies (five publications) [21, 23–25, 48] reported using a validated FFQ and four of these studies (two publications) [23, 24] made efforts to account for dietary changes during follow-up through updated analyses using data from repeated measurements.

Age ranged from 10 to 90 years at baseline across studies. In the studies that included non-adult participants, the participants below < 18 years are likely to have made up a small percentage of the total population. No studies were performed exclusively in pregnant or breastfeeding women.

Follow-up ranged from 5.14 to 28.3 years. The number of incident cases or deaths ranged from 219 to 1117 for circulatory disease, 375 to 106,690 for CVD, 60 to 24,794 for IHD, 31 to 909 for cerebrovascular disease, 54 to 5946 for total stroke, 31 to 519 for ischemic stroke and 28 to 300 for hemorrhagic stroke. The sample size ranged from 1724 to 422,791 participants across all outcomes. All studies used record linkage for outcome ascertainment except the Heidelberg Study [27], which retrieved copies of death certificates from the Registrar’s office.

The basis for RoB judgements are shown in *Supplementary Tables* 5, 6 and *8* (Online Resource 4)*,* and results of RoB judgements for the domains and for each study overall are shown for CVD, IHD and stroke separately in *Supplementary Tables 9 and 10* (Online Resource 4), *and Supplementary Figs. 1, 2, 3* (Online Resource 4). In the overall (study-level) RoB assessment for CVD and IHD, we judged three studies (three publications) [21–23] to be at ‘moderate’ and five studies (three publications) [20, 26, 27] to be at ‘serious’ RoB. In the overall (study-level) RoB assessment for stroke, we judged seven studies (four publications) [22–25] to be at ‘moderate’, and five studies (two publications) [20, 26] to be at ‘serious’ RoB.

Most studies included a statement on funding (see their original papers) [21, 23–25, 47–49], and no studies reported industry funding, but two studies (two publications) [27, 50] did not report on funding.

### Cardiovascular disease

**Fig. 2:**
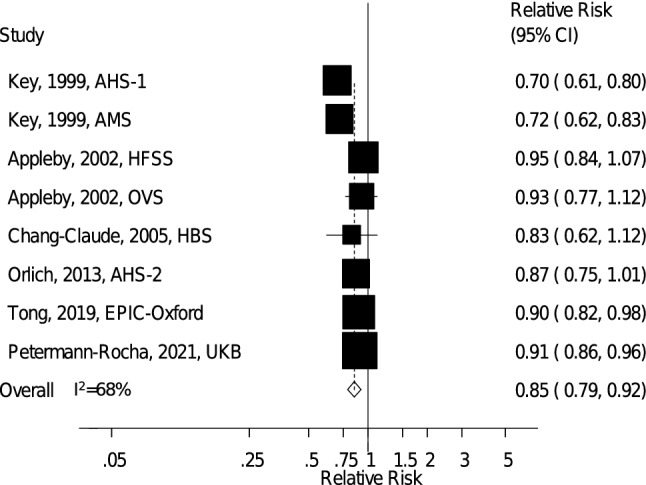
Vegetarian vs. nonvegetarian diets and the risk of cardiovascular disease. *AHS-1* Adventist Health Study 1, *AHS-2* Adventist Health Study 2, *AMS* Adventist Mortality Study, *EPIC-Oxford* European Prospective Investigation into Cancer and Nutrition – Oxford, *HBS* Heidelberg Study, *HFSS* Health Food Shoppers Study, *OVS* Oxford Vegetarian Study, *UKB* UK Biobank

Eight prospective cohort studies (six publications) [20–23, 26, 27] with 621,282 participants and 115,392 CVD cases were included in the analysis of vegetarian diets and CVD. The summary RR for vegetarians compared to nonvegetarians was 0.85 (95% CI: 0.79–0.92, *I*^2^ = 68%, p_heterogeneity_ = 0.003) (Fig. 2), showing reduced CVD risk for vegetarians. There was no indication of publication bias with Egger's test (*p* = 0.28), Begg's test (*p* = 0.39), or by inspection of the funnel plot (*Supplementary Fig. 4*, Online Resource 4). The summary RRs (95% CI) ranged from 0.84 (0.77–0.91, *I*^2^ = 70%) when excluding the Health Food Shoppers Study to 0.88 (0.83–0.94, *I*^2^ = 42%) when excluding the Adventist Health Study 1 (*Supplementary Fig. 5,* Online Resource 4).

**Fig. 3:**
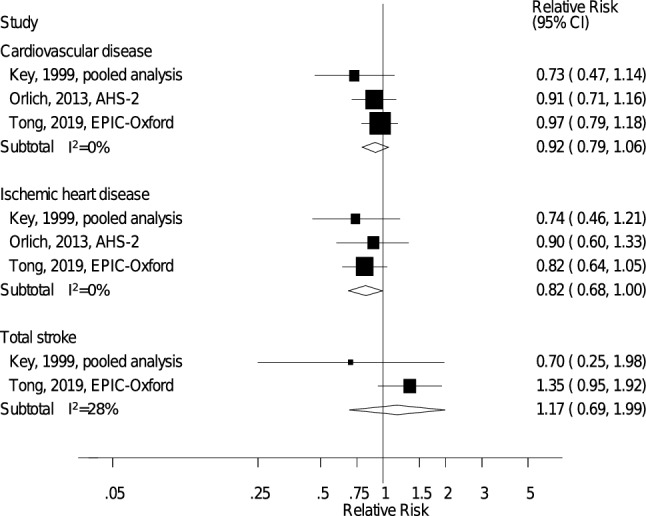
Vegan vs. nonvegetarian diets and the risk of cardiovascular disease, ischemic heart disease and total stroke. The summary relative risk estimate (RR) from the pooled analysis by Key et al. 1999 were based on data from 4 studies: Adventist Health Study 1, Adventist Mortality Study, Heidelberg Study, Oxford Vegetarian Study. *AHS-1* Adventist Health Study 1, *AHS-2* Adventist Health Study 2, *AMS* Adventist Mortality Study, *EPIC-Oxford* European Prospective Investigation into Cancer and Nutrition – Oxford, *HBS* Heidelberg Study, *OVS* Oxford Vegetarian Study

Six studies (three publications, three risk estimates) [20, 21, 23] including 197,668 participants and 8052 CVD cases were included in the analysis of vegan diets and the risk of CVD, and the summary RR (95% CI) was 0.92 (0.79–1.06, *I*^2^ = 0%, p_heterogeneity_ = 0.52) (Fig. 3), suggesting no clear association with risk of CVD for vegans.

### Ischemic heart disease

**Fig. 4:**
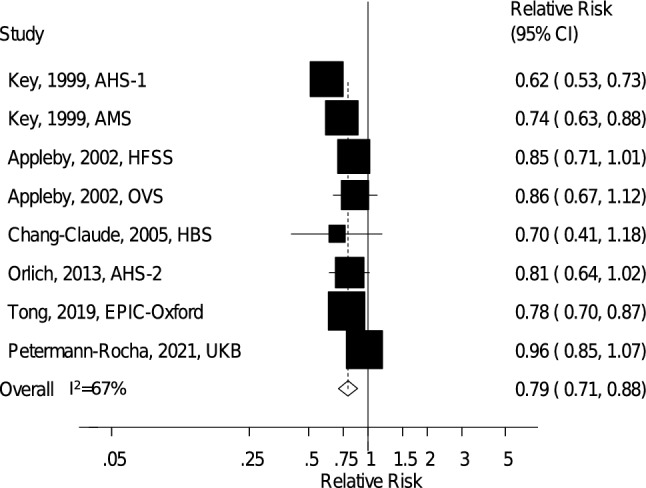
Vegetarian vs. nonvegetarian diets and the risk of ischemic heart disease. *AHS-1* Adventist Health Study 1, *AHS-2* Adventist Health Study 2, *AMS* Adventist Mortality Study, *EPIC-Oxford* European Prospective Investigation into Cancer and Nutrition – Oxford, *HBS* Heidelberg Study, *HFSS* Health Food Shoppers Study, *OVS* Oxford Vegetarian Study, *UKB* UK Biobank

Eight prospective cohort studies (six publications) [20–23, 26, 27] including 621,282 participants and 30,377 IHD cases were included in the analysis of vegetarian diets and IHD. The summary RR for vegetarians compared to nonvegetarians was 0.79 (95% CI: 0.71–0.88, *I*^2^ = 67%, p_heterogeneity_ = 0.003) (Fig. 4), showing reduced IHD risk for vegetarians. There was no indication of publication bias with Egger's test (*p* = 0.61), Begg's test (*p* = 0.90), or by inspection of the funnel plot (*Supplementary Fig. 6*, Online Resource 4). The summary RR (95% CI) ranged from 0.76 (0.70–0.83, *I*^2^ = 36%) when excluding UK Biobank to 0.83 (0.76–0.91, *I*^2^ = 38%) when excluding Adventist Health Study 1 (*Supplementary Fig. 7*, Online Resource 4).

Six studies (three publications, three risk estimates) [20, 21, 23] including 197,668 participants and 5456 IHD cases were included in the analysis of vegan diets and IHD, and the summary RR (95% CI) was 0.82 (0.68–1.00, *I*^2^ = 0%, p_heterogeneity_ = 0.83) (Fig. 3), suggesting reduced IHD risk for vegans.

### Stroke

**Fig. 5:**
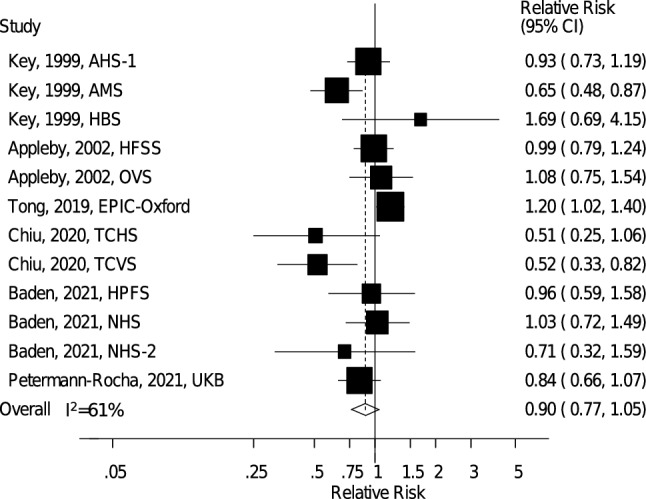
Vegetarian vs. nonvegetarian diets and the risk of total stroke. *AHS-1* Adventist Health Study 1, *AMS* Adventist Mortality Study, *EPIC-Oxford* European Prospective Investigation into Cancer and Nutrition – Oxford, *HBS* Heidelberg Study, *HFSS* Health Food Shoppers Study, *HPFS* Health Professionals Follow-up Study, *NHS1* Nurses’ Health Study 1, *NHS2* Nurses’ Health Study 2, *OVS* Oxford Vegetarian Study, *TCHS* Tzu Chi Health Study, *TCVS* Tzu Chi Vegetarian Study, *UKB* UK Biobank

Twelve prospective cohort studies (six publications) [20, 22–26] including 770,867 participants and 14,419 stroke cases were included in the analysis of vegetarian diets and total stroke. The summary RR for vegetarians compared to nonvegetarians was 0.90 (95% CI: 0.77–1.05, *I*^2^ = 61%, p_heterogeneity_ = 0.003) (Fig. 5), suggesting no clear association with total stroke for vegetarians. There was no indication of publication bias with Egger's test (*p* = 0.15), Begg's test (*p* = 0.63), or by inspection of the funnel plot (*Supplementary Fig. 8,* Online Resource 4). The summary RR (95% CI) ranged from 0.86 (0.74–0.99, *I*^2^ = 41%) when excluding EPIC-Oxford to 0.94 (0.81–1.08, *I*^2^ = 52%) when excluding Tzu Chi Vegetarian Study (*Supplementary Fig. 9*, Online Resource 4).

**Fig. 6:**
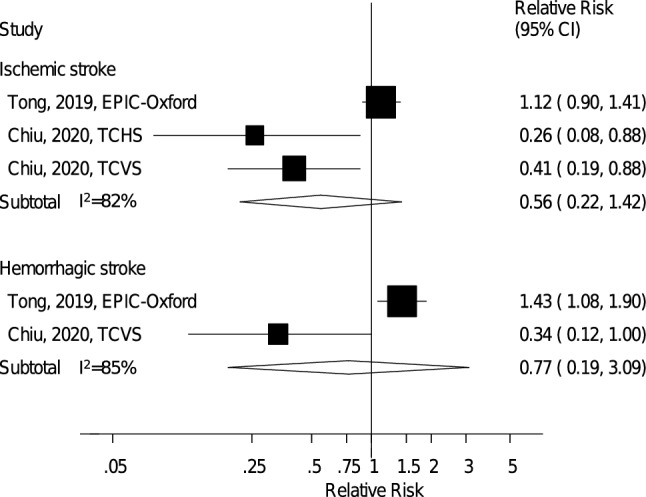
Vegetarian vs. nonvegetarian diets and the risk of ischemic and hemorrhagic stroke. *EPIC-Oxford* European Prospective Investigation into Cancer and Nutrition – Oxford, *TCHS* Tzu Chi Health Study, *TCVS* Tzu Chi Vegetarian Study

When subtypes of stroke were analysed, the summary RR for vegetarians vs. nonvegetarians was 0.56 (95% CI: 0.22–1.42, *I*^2^ = 82%, p_heterogeneity_ = 0.004, *n* = 3) for ischemic stroke (Fig. 6) and 0.77 (95% CI: 0.19–3.09, *I*^2^ = 85%, p_heterogeneity_ = 0.01, *n* = 2) for hemorrhagic stroke (Fig. 6), suggesting no clear association with either outcome.

Five studies (two publications, two risk estimates) [20, 23] including 109,938 participants and ≥ 39 cases were included in the analysis of vegan diets and the risk of total stroke, and the summary RR (95% CI) was 1.17 (0.69–1.99, *I*^2^ = 28%, P_heterogeneity_ = 0.24) (Fig. 3), suggesting no clear association with total stroke for vegans.

### Subgroup and sensitivity analyses

*Supplementary Tables 11* and *12* show results from all subgroup analyses. Although there was high heterogeneity in our main analysis as measured by *I*^2^, this was for CVD and IHD mainly driven by differences in the strength of the association, rather than due to differences in the direction of the association, as all studies reported risk estimates in the direction of an inverse association. For stroke, the results were less consistent as there was heterogeneity also with regard to the direction of the association.

For CVD and IHD, the inverse associations persisted in subgroup analyses stratified by sex, duration of follow-up, exclusion of early follow-up, outcome subtype, exclusion of prevalent disease at baseline, geographic location, number of cases, risk of bias and adjustment for confounding factors. In the analysis of CVD, there was little evidence of heterogeneity between subgroups, with the exception of the subgroup analysis stratified by geographic location, which showed a stronger association in North American studies than in European studies (Supplementary Table 11). In the analysis of IHD, there was also little evidence of heterogeneity between subgroups, with the exception of the subgroup analysis stratified by whether early follow-up was included or not, which showed a weaker association when early follow-up was excluded compared to when it was included (Supplementary Table 12). When comparing the associations for vegetarian diets and IHD among studies that provided risk estimates both adjusted and not adjusted for BMI, the association was slightly stronger when not adjusted for BMI (18% vs. 22% reduction in risk), suggesting that approximately 1/5 of the association potentially could be mediated by BMI (Supplementary Table 12). The null results for stroke persisted in most subgroup analyses; however, there was heterogeneity between subgroups when analyses were stratified by duration of follow-up and geographic location with inverse associations among studies with shorter vs. longer follow-up and among two Asian (Taiwanese) studies, but not among European or American studies (Supplementary Table 12).

To assess the impact of more rigorous adjustment on the summary estimate we conducted a sensitivity analysis using the more adjusted risk estimates (adjusted for alcohol, education, exercise, and BMI in addition to age, sex and smoking status) from the pooled analysis of Key et al. [20], and replaced the results from the overlapping individual studies (AMS, AHS-1, OVS, HBS) with the results from this pooled analysis (more adjusted results from each individual study were not provided in the publication so we used the pooled estimate). The summary RR when incorporating the more adjusted estimate from the pooled analysis in our meta-analysis was 0.81 (95% CI: 0.72–0.92, *I*^2^ = 71%, p_heterogeneity_ = 0.009) while the summary estimate using the less adjusted model was 0.79 (95% CI: 0.68–0.93, *I*^2^ = 84%, p_heterogeneity_ < 0.0001), suggesting little difference in the overall findings.

E-values for the association between vegetarian diets and CVD and IHD were 1.64 (lower CI: 1.40) and 1.86 (lower CI: 1.49), and e-value for vegan diets and IHD was 1.74 (lower CI: 1.00).

### Grading of evidence

Using WCRF criteria for judging the evidence, we considered the overall evidence to indicate a probable protective causal association between vegetarian diets and reduced risk of CVD and IHD, and for stroke, the evidence was deemed limited-no conclusion (*Supplementary Table 13* and *14*, Online Resource 4) [36, 37]. We considered the evidence on vegan diets and reduced risk of IHD to be limited-suggestive, and for CVD and stroke to be limited-no conclusion. This was mainly due to the limited number of studies and lack of precision for the association with IHD (but similar effect size as for vegetarians), and weaker and less clear associations for CVD and stroke (Supplementary Table 13, 14, Online Resource 4). Detailed justifications for the judgements on vegetarian diets and CVD, IHD and stroke are outlined in Supplementary Table 13 (Online Resource 4) and include, clear inverse associations based on data from eight cohort studies [20–23, 26, 27] that were robust in most subgroup and sensitivity analyses, no evidence of publication bias, and observed heterogeneity that was driven more by differences in the strength of the associations rather than differences in the direction of the associations. There is also supporting evidence from randomized trials that vegetarian diets reduce cardiovascular risk factors, including total and LDL cholesterol [51], systolic and diastolic blood pressure [12] and weight gain [52], and there is consistent evidence that vegetarian diets reduce the risk of type 2 diabetes from cohort studies [53–55] (see discussion for details). There is also strong evidence that consumption of several food groups, which often differ between vegetarians and nonvegetarians (e.g., red meat, processed meat, fruits, vegetables, whole grains, nuts, legumes) are associated with risk of CVD and IHD [56–63] in a manner that is consistent with the results observed for vegetarians (see discussion for details).

## Discussion

The present systematic review and meta-analysis showed a 15% and a 21% reduction in the relative risk of CVD and IHD, respectively, for vegetarians compared to nonvegetarians, but no clear association was observed for total stroke or subtypes of stroke. In addition, an 18% reduction in the relative risk of IHD was observed among vegans when compared to nonvegetarians, although this association was imprecise. No clear association was observed between vegan diets and CVD or stroke; however, the number of studies was limited. Although there was high heterogeneity in the main analyses for CVD and IHD, this was largely explained by differences in the strength of the associations as all studies reported risk estimates in the direction of an inverse association. For CVD, the association was stronger among North American studies than among the European studies. For stroke, studies were less consistent, and an inverse association was observed only among Asian studies and not in European or American studies. There was no indication of publication bias in the three main analyses. The findings regarding IHD are consistent with previous meta-analyses [10, 64–67]; however, to our knowledge this is the first meta-analysis to report a clear reduction in CVD risk overall as well, while previous meta-analyses found no association for CVD [10, 65–67]. This difference is likely due to the larger sample size and greater statistical power in the current analysis. Our finding of no association for stroke is consistent with previous meta-analyses on vegetarian diets and total stroke or cerebrovascular disease [10, 64–67]. However, considering the results of the influence analysis, we cannot entirely rule out a weak to moderate inverse association, but further larger studies are needed to clarify this.

Our analysis has several limitations as well as strengths. Although the meta-analysis was not registered with a pre-defined analysis plan or study protocol, the analysis used a similar format to previous meta-analyses [61–63] and efforts were made to ensure transparency of the work. Although the title and abstract screening was only performed by one author (JSD), the second part (full-text assessment) was performed by two authors (JSD, DA).

Heterogeneity was an apparent issue across all outcomes. However, heterogeneity is expected for a number of reasons, including differences in the (1) detail of the dietary assessment methods used, (2) geographic location and background food choices and dietary patterns [21], (3) confounders that were adjusted for in the statistical analyses, (4) sample size and duration of follow-up, and (5) stability of the diet over time in different studies. Most of the results for CVD and IHD were similar across subgroup analyses. In contrast, results for stroke were less clear and somewhat inconsistent, and there was heterogeneity between subgroups in the subgroup analyses stratified by geographic location with an inverse association in Asian studies, but no clear association was observed in North America or Europe. Whether these differences in results are due to differences in dietary habits between the Asian and the US and European studies, or whether it is simply a play of chance is unclear. Further studies are therefore needed to clarify this association and to explain the potential geographic variations in the results.

Vegetarians are often more health-conscious than nonvegetarians, and given the observational design of the included studies, confounding from other lifestyle factors could be an issue. However, the results persisted across multiple subgroup analyses of studies that adjusted for age, education, alcohol, smoking, BMI and physical activity, and there was little indication of heterogeneity between these subgroups. In a pooled analysis, further adjustment for alcohol, education, exercise and BMI in addition to adjustment for age, sex, and smoking status made little impact on the association between vegetarian diets and IHD [20], and when we used the more adjusted results from this pooled analysis there was also little change in the summary estimates, suggesting little confounding from these factors. Although residual confounding could be an issue, the inverse association between vegetarian diets and IHD persisted across strata of smoking and the presence of other risk factors in the EPIC-Oxford study, and inverse associations were also observed in the Adventist Health and Mortality Studies, populations consisting largely of non-smokers and non-alcohol drinkers, which might suggest an independent effect of a vegetarian diet on risk of IHD and CVD [20, 21, 23]. The estimated E-values suggest that any unadjusted confounders would have to be relatively strongly (RR = 1.64–1.86) associated with both vegetarian and vegan diets and with risk of CVD and IHD to fully explain away these associations. The results also persisted across strata of risk of bias and there was no between subgroup heterogeneity detected with meta-regression analyses. Although publication bias can affect meta-analyses of published studies, we found no indication of publication bias with the statistical tests used or by inspection of the funnel plots.

The definitions of vegetarian and nonvegetarian diets were not entirely uniform across studies, and it is possible that this could have affected the results; however, we do not expect a substantial impact of this on the overall results. In most studies, vegetarian status was defined based on meat and fish consumption reported on food frequency questionnaires, while in the Health Food Shoppers Study [26] participants were asked whether they identified as vegetarians or nonvegetarians. Nevertheless, exclusion of this study did not materially alter any of the observed associations, suggesting little impact of this study on the overall conclusions. Meat consumption has been reported to be markedly lower among the nonvegetarians in the Adventist Health Study 2 [68, 69], EPIC-Oxford [70], and the UK Biobank [71] when compared to the general population [72, 73]. If differences in meat consumption account for some of the difference in cardiovascular risk between vegetarians and nonvegetarians, this could potentially lead to conservative estimates of the true associations compared to if a more representative comparison group had been available.

As exposure assessment in most cohorts was only conducted at baseline (except in EPIC-Oxford, Health Professionals Follow-up Study, Nurses’ Health Study 1, and Nurses’ Health Study 2 [23, 24]), participants may have changed their diet during the course of follow-up. This could lead to misclassification of dietary habits, which given the prospective design of the studies, would likely be non-differential and might bias the summary estimates toward the null. In the EPIC-Oxford study, there was little difference in the hazard ratios by whether repeated measures or only baseline data were used to analyse the association between vegetarian diets and IHD [23]. However, other studies have reported considerable differences in the association between red and processed meat intake and CVD mortality when comparing repeated measures vs. only baseline measures [59]. The stability of vegetarian status or meat consumption over time could differ between studies, but further studies are needed to address this question.

The current analysis was not able to assess the association between quality of vegetarian (or vegan) diets and CVD, IHD or stroke risk, as there were no studies that have investigated this directly to date. Other studies that have assessed the association between plant-based dietary indices and CVD risk have reported inverse associations between plant-based dietary indices overall as well as for healthy plant-based dietary indices (characterized by high intake of whole plant foods) and CVD risk, while unhealthy plant-based dietary indices (characterized by high intake of sugar-sweetened beverages, French fries, chips, cookies, and other fast foods) have been associated with increased CVD risk [74], suggesting the importance of emphasizing whole plant foods.

Strengths of our meta-analysis include the following: (1) the detailed search strategy; (2) rigorous and comprehensive risk of bias assessments that more adequately assess internal validity of the included studies (ROBINS-I) [30, 31, 33, 34], with results implemented in synthesis through subgroup analyses; (3) increased sample size and statistical power which allowed detection of moderate associations between vegetarian diets and both CVD and IHD and (4) the detailed subgroup and sensitivity analyses, which supported the robustness of the findings.

The current findings are consistent with other epidemiological and experimental studies which have found that vegetarians have a lower BMI [10] and reduced weight gain [52, 75], lower serum total and LDL-cholesterol [51], lower blood pressure or prevalence of hypertension [12–14] and lower risk of type 2 diabetes [16] than nonvegetarians, all of which are important cardiovascular risk factors. Meta-analyses of randomized trials reported a 0.32-mmol/l (12.2 mg/dl) reduction in LDL-cholesterol [9] and a 4.8-mmHg reduction in systolic blood pressure with consumption of vegetarian vs. nonvegetarian diets [12]. Such a difference in LDL-cholesterol and systolic blood pressure would predict a 10% and 11% lower relative risk of IHD mortality, respectively, based on data from a pooled analysis of 61 prospective studies [76, 77]. If LDL-cholesterol and systolic blood pressure are assumed to act additively, these differences could largely explain the 21% reduction in the relative risk of IHD we observed among vegetarians vs. nonvegetarians. Differences in adiposity and type 2 diabetes risk could also contribute towards a lower IHD risk; however, some of the difference in LDL-cholesterol and systolic blood pressure, as well as a sizeable part of the reduction in risk of type 2 diabetes among vegetarians versus nonvegetarians, is likely driven by differences in BMI. In the current analysis, adjustment for BMI attenuated the association between vegetarian diets and IHD by approximately one-fifth, suggesting a modest part of the difference in IHD risk might be mediated by differences in BMI.

Our results are also consistent with studies on food groups and risk of CVD and IHD. Several cohort studies have shown an increased risk not only of both CVD and IHD [56–60], but also for stroke associated with consumption of red and processed meat [78]. Red and processed meat are major sources of dietary saturated fat and cholesterol, which are known to increase serum cholesterol [79] and could thereby increase the risk of IHD. Experimental studies in mice have also shown that red meat increases atherosclerosis by increasing the production of trimethylamine-N-oxide (TMAO) through a gut-dependent pathway [80]. In fact, vegetarians exhibit lower levels of TMAO, suggesting a link between meat consumption and CVD risk [80]. In addition, processed meats are a major source of salt, which could increase CVD risk through increased blood pressure [81], and both red and processed meat intake have been associated with increased weight gain [82] and type 2 diabetes [83], which could increase CVD risk. However, other dietary differences could also contribute toward reduced CVD risk among vegetarians. Compared to meat-eaters, vegetarians tend to have a higher intake of fruit and vegetables, whole grains, nuts, and legumes [70], and such food groups have generally been shown to reduce the risk of CVD, IHD and stroke [61–63] and to have benefits on cardiovascular risk factors such as blood pressure [84–86], serum cholesterol [87–89], bodyweight [82] and risk of type 2 diabetes [90, 91].

The present meta-analysis suggests a vegetarian diet offers important health benefits by reducing the risk of both CVD and IHD, although not stroke. These are findings with important public health implications given that CVDs still are the leading causes of death and disease globally and suggest that adoption of plant-based dietary patterns such as vegetarian diets can be useful for reducing the CVD burden. These findings support a stronger emphasis on vegetarian dietary patterns in public health recommendations as a measure for CVD prevention.

Future research should focus on additional large-scale and high-quality studies as they are needed to clarify results for stroke and stroke subtypes, as well as to provide results stratified by other risk factors and results with adequate adjustment for confounding factors to better rule out potential residual confounding. Further studies from other geographic regions are also needed. Detailed and repeated dietary assessments may be important to take into account dietary changes during follow-up. Future studies should focus on recruiting more vegans as there were few studies with sufficient numbers to detect a clear association among the vegans.

In conclusion, a 15% reduction in the relative risk of CVD and a 21% reduction in the relative risk of IHD was observed for vegetarians compared to nonvegetarians, but no clear association was observed for total stroke or stroke subtypes. There was an 18% reduction in the relative risk of IHD among vegans, but the association lacked precision and no clear association was observed for CVD or stroke; however, there were few studies in the analyses of vegans. These findings are consistent with existing guidelines recommending plant-based dietary patterns for CVD prevention but suggest more emphasis may be put on vegetarian diets. Further studies are needed to clarify the association between vegetarian diets and stroke risk, as well as the association between vegan diets and CVD, IHD and stroke.

## Supplementary Information

Below is the link to the electronic supplementary material.

Supplementary file1 (PDF 59 KB)

Supplementary file2 (PDF 16 KB)

﻿Supplementary file3 (PDF 255 KB)

Supplementary file4 (PDF 1269 KB)

## Data Availability

Data, material and analytical code will be made available upon reasonable request. Statistical analysis was performed with Stata SE version 15 (StataCorp, TX, USA).

## Abbreviations

AHS-1: Adventist Health Study 1
AHS-2: Adventist Health Study 2
AMS: Adventist Mortality Study
CVD: Cardiovascular disease
EPIC-Oxford: European Prospective Investigation into Cancer and Nutrition—Oxford
HBS: Heidelberg Study
HFSS: Health Food Shoppers Study
HPFS: Health Professionals Follow-up Study
IHD: Ischemic heart disease
NHS1: Nurses’ Health Study 1
NHS2: Nurses’ Health Study 2
OVS: Oxford Vegetarian Study
PRISMA: Preferred Reporting Items for Systematic Reviews and Meta-Analyses
RoB: Risk of Bias
ROBINS-I: Risk Of Bias In Non-randomized Studies of Interventions
TCHS: Tzu Chi Health Study
TCVS: Tzu Chi Vegetarian Study
TMAO: Trimethylamine-N-oxide
UKB: UK Biobank
