# Supplementary material for: Vegetarian and vegan diets and the risk of cardiovascular disease, ischemic heart disease and stroke: a systematic review and meta-analysis of prospective cohort studies
Source: Eur J Nutr. 2022 Aug 27;62(1):51–69. doi: 10.1007/s00394-022-02942-8 (PMC9899747; doi:10.1007/s00394-022-02942-8)
Supplement: Supplementary file 2 — Supplementary file2 (PDF 16 KB) [file 394_2022_2942_MOESM2_ESM.pdf]

Online Resource 2

Supplementary material to: Vegetarian and vegan diets and the risk of cardiovascular disease, ischemic heart disease and stroke: a systematic review and meta-analysis of prospective cohort studies.

Authors: Jarle Sæby Dybvik[1], Mette Svendsen[2,3] Dagfinn Aune[3,4,5,6]

Author affiliations

[1] Institute of Clinical Medicine, Faculty of Medicine, University of Oslo, Oslo, Norway

[2] Department of Nutrition Sciences, Institute of Basic Medical Sciences, University of Oslo, Oslo, Norway

[3] Department of Endocrinology, Morbid Obesity and Preventive Medicine, Oslo University Hospital, Oslo, Norway

[4] Department of Epidemiology and Biostatistics, School of Public Health, Imperial College London, London, United Kingdom

[5] Department of Nutrition, Oslo New University College, Oslo, Norway

[6] Unit of Cardiovascular and Nutritional Epidemiology, Institute of Environmental Medicine, Karolinska Institutet, Stockholm, Sweden

Correspondence to: Jarle Sæby Dybvik, Institute of Clinical Medicine, Faculty of Medicine, University of Oslo, Oslo, Norway. E-mail address: j.s.dybvik@gmail.com. Phone number: 0047 99162306

Supplementary Table 3. PubMed and Ovid Embase search strategy

PubMed	((cardiovascular diseases [Mesh] OR cardiovascular disease OR heart diseases [Mesh] OR heart disease OR ischemic heart disease OR myocardial ischemia [Mesh] OR myocardial ischaemia OR coronary artery disease [Mesh] OR coronary disease [Mesh] OR coronary heart disease OR acute coronary syndrome [Mesh] OR angina pectoris [Mesh] OR myocardial infarction [Mesh] OR cerebrovascular disorders [Mesh] OR cerebrovascular disease OR cerebrovascular accident OR brain attack OR carotid artery diseases [Mesh] OR carotid artery disease OR cerebral small vessel diseases [Mesh] OR cerebral small vessel disease OR intracranial artery disease OR cerebral arterial diseases [Mesh] OR cerebral artery disease OR intracranial arteriosclerosis [Mesh] OR intracranial embolism and thrombosis [Mesh] OR intracranial hemorrhages [Mesh] OR intracranial hemorrhage OR intracranial haemorrhage OR cerebral hemorrhage [Mesh] OR cerebral haemorrhage OR intracerebral hemorrhage OR intracerebral haemorrhage OR subarachnoid hemorrhage [Mesh] OR subarachnoid haemorrhage OR stroke [Mesh] OR brain infarction [Mesh] OR cerebral infarction [Mesh] OR cerebral ischemia OR cerebral ischaemia OR brain ischemia [Mesh] OR brain ischaemia OR brain hemorrhage OR brain haemorrhage)<br><br>AND<br><br>(vegetar* OR vegetarians [Mesh] OR diet, vegetarian [Mesh] OR vegetarian diet OR vegan* OR vegans [Mesh] OR diet, vegan [Mesh] OR vegan diet OR

	adventist OR adventists OR lacto-ovo vegetarian OR lacto-ovo vegetarians OR lacto-vegetarian diet))
Ovid Embase	((exp cardiovascular disease [Emtree] OR exp heart disease [Emtree] OR exp ischemic heart disease [Emtree] OR exp coronary artery disease [Emtree] OR exp acute coronary syndrome [Emtree] OR exp angina pectoris [Emtree] OR exp heart infarction [Emtree] OR exp heart muscle ischemia [Emtree] OR cardiovascular disease.tw. OR heart disease.tw. OR ischemic heart disease.tw. OR coronary heart disease.tw. OR myocardial ischemia.tw. OR acute coronary syndrome.tw. OR angina pectoris.tw. OR myocardial infarction.tw. OR exp cerebrovascular disease [Emtree] OR exp brain hematoma [Emtree] OR exp brain hemorrhage [Emtree] OR exp brain infarction [Emtree] OR exp brain ischemia [Emtree] OR exp carotid artery disease [Emtree] OR exp cerebral artery disease [Emtree] OR exp cerebrovascular accident [Emtree] OR exp occlusive cerebrovascular disease [Emtree] OR exp subarachnoid hemorrhage [Emtree] OR exp brain embolism [Emtree] OR cerebrovascular disease.tw. OR cerebrovascular disorders.tw. OR brain ischemia.tw. OR cerebral ischemia.tw. OR stroke.tw. OR brain attack.tw. OR brain infarction.tw. OR cerebral infarction.tw. OR brain hemorrhage.tw. OR cerebral hemorrhage.tw. OR brain haemorrhage.tw. OR cerebral haemorrhage.tw. OR intracranial hemorrhage.tw. OR intracranial haemorrhage.tw. OR intracerebral hemorrhage.tw. OR intracerebral haemorrhage.tw. OR subarachnoid hemorrhage.tw. OR subarachnoid haemorrhage.tw. OR cerebral embolism.tw. OR cerebral thrombosis.tw.)<br><br>AND

	(exp vegetarian diet [Emtree] OR exp lactoovovegetarian diet [Emtree] OR lactovegetarian diet [Emtree] OR exp vegan diet [Emtree] OR exp vegetarian [Emtree] OR lactoovovegetarian [Emtree] OR lactovegetarian [Emtree] OR exp ovovegetarian diet/ OR vegetarian.tw. OR vegetarians.tw. OR vegetarianism.tw. OR vegan.tw. OR vegans.tw. OR veganism.tw. OR exp Seventh-day Adventist [Emtree] OR exp Adventist [Emtree] OR adventist.tw. OR adventists.tw. OR adventism.tw.))
