# Supplementary material for: Vegetarian and vegan diets and the risk of cardiovascular disease, ischemic heart disease and stroke: a systematic review and meta-analysis of prospective cohort studies
Source: Eur J Nutr. 2022 Aug 27;62(1):51–69. doi: 10.1007/s00394-022-02942-8 (PMC9899747; doi:10.1007/s00394-022-02942-8)
Supplement: Supplementary file 3 — ﻿Supplementary file3 (PDF 255 KB) [file 394_2022_2942_MOESM3_ESM.pdf]

Online Resource 3

Supplementary material to: Vegetarian and vegan diets and the risk of cardiovascular disease, ischemic heart disease and stroke: a systematic review and meta-analysis of prospective cohort studies.

Authors: Jarle Sæby Dybvik[1], Mette Svendsen[2,3] Dagfinn Aune[3,4,5,6]

Author affiliations

[1] Institute of Clinical Medicine, Faculty of Medicine, University of Oslo, Oslo, Norway

[2] Department of Nutrition Sciences, Institute of Basic Medical Sciences, University of Oslo, Oslo, Norway

[3] Department of Endocrinology, Morbid Obesity and Preventive Medicine, Oslo University Hospital, Oslo, Norway

[4] Department of Epidemiology and Biostatistics, School of Public Health, Imperial College London, London, United Kingdom

[5] Department of Nutrition, Oslo New University College, Oslo, Norway

[6] Unit of Cardiovascular and Nutritional Epidemiology, Institute of Environmental Medicine, Karolinska Institutet, Stockholm, Sweden

Correspondence to: Jarle Sæby Dybvik, Institute of Clinical Medicine, Faculty of Medicine, University of Oslo, Oslo, Norway. E-mail address: j.s.dybvik@gmail.com. Phone number: 0047 99162306

Supplementary Table 4. Citations excluded after full-text assessment

**Exclusion criteria**	**Reference number**
Case-control studies	[1,2]
Cohort profiles	[3,4]
Conference papers / abstracts	[5-20]
Cross-sectional studies	[21-26]
Duplicates	[27-39]
Letters / editorials / commentaries	[40-74]
Meta-analyses	[75-81]
No risk estimates / confidence intervals / not useable results	[82-87]
Not relevant exposure	[88-187]
Not relevant exposure / outcome	[188-196]
Not relevant outcome	[197-216]
Retracted article	[217]
Reviews	[218-319]
Studies in subjects with diabetes	[320]
Studies in subjects with IHD or stroke	[321-344]
Study protocol	[345]

Reference list of citations excluded after full-text assessment

1. Bhattacharjee M, Dalal PM, Vairale J, Shenoy A (2015) In hospital prospective stroke registry-risk factor survey. Int J Stroke 10:235. doi:http://dx.doi.org/10.1111/ijs.12479
2. Pais P, Pogue J, Gerstein H, Zachariah E, Savitha D, Jayprakash S, Nayak PR, Yusuf S (1996) Risk factors for acute myocardial infarction in Indians: a case-control study. Lancet (London, England) 348 (9024):358-363. doi:10.1016/s0140-6736(96)02507-x
3. Butler TL, Fraser GE, Beeson WL, Knutsen SF, Herring RP, Chan J, Sabate J, Montgomery S, Haddad E, Preston-Martin S, Bennett H, Jaceldo-Siegl K (2008) Cohort profile: The Adventist Health Study-2 (AHS-2). Int J Epidemiol 37 (2):260-265. doi:http://dx.doi.org/10.1093/ije/dym165
4. Cade JE, Burley VJ, Greenwood DC, Group UKWsCSS (2004) The UK Women's Cohort Study: comparison of vegetarians, fish-eaters and meat-eaters. Public Health Nutr 7 (7):871-878. doi:10.1079/phn2004620
5. Mejia MA, Sanchez A, Sanchez J, Runte E (2016) A vegan diet rich in fats of plant origin may reverse Coronary artery disease. FASEB Journal Conference: Experimental Biology 30 (Meeting Abstracts)
6. Mangat A, Grewal D, Singh R, Jyotsna R, Kaur P, Pandian JD (2011) Dietary patterns in stroke patients in northwest India. Cerebrovasc Dis 32:27. doi:http://dx.doi.org/10.1159/000333802
7. Heskey C (2017) The contribution of plant-based dietary patterns to health and disease. West Indian Med J 66 (Supplement 3):31-32
8. Zyriax BC (2019) Longterm consequences of diet styles (vegetarian, vegan, low-carb ...). Maturitas 124:113. doi:http://dx.doi.org/10.1016/j.maturitas.2019.04.012
9. Sonja Smiljic V (2019) Lifestyles as risk for cardiovascular diseases in medical students. Eur J Heart Fail 21 (Supplement 1):145. doi:http://dx.doi.org/10.1002/ejhf.1488
10. Dinu M, Pagliai G, Casini A, Sofi F (2016) Vegetarian, vegan diets and multiple health outcomes: A systematic review with meta-analysis of observational studies. Eur Heart J 37 (suppl.1):191-598. doi:http://dx.doi.org/10.1093/eurheartj/ehw432
11. Appleby PN, Key TJ (2016) The long-term health of vegetarians and vegans. The Proceedings of the Nutrition Society 75 (3):287-293. doi:10.1017/S0029665115004334
12. Chhajer B, Singh V, Kumari G (2016) Development of a holistic model to prevent and reverse coronary artery disease: SAAOL (science and art of living) safety circle. Cardiology (Switzerland) 134 (Supplement 1):309. doi:http://dx.doi.org/10.1159/000447505
13. Orlich MJ, Fraser GE (2014) Vegetarian diets in the Adventist Health Study 2: a review of initial published findings. The American journal of clinical nutrition 100 Suppl 1 (1):353S-358S. doi:10.3945/ajcn.113.071233
14. Willett WC (1999) Convergence of philosophy and science: the third international congress on vegetarian nutrition. The American journal of clinical nutrition 70 (3 Suppl):434S-438S. doi:10.1093/ajcn/70.3.434s
15. Willett W (2003) Lessons from dietary studies in Adventists and questions for the future. The American journal of clinical nutrition 78 (3 Suppl):539S-543S. doi:10.1093/ajcn/78.3.539S
16. Knutsen SF (1994) Lifestyle and the use of health services. Am J Clin Nutr 59 (5 SUPPL.):1171S-1175S. doi:http://dx.doi.org/10.1093/ajcn/59.5.1171S
17. Crowe FL, Appleby PN, Travis RC, Key TJ (2012) Risk of ischaemic heart disease among British vegetarians and non-vegetarians: Results from the EPIC-Oxford cohort study. Proceedings of the Nutrition Society Conference: Summer Meeting of the Nutrition Society Hosted by the Irish Section 71 (OCE2):E203. doi:http://dx.doi.org/10.1017/S0029665112002601

18. Obersby D, Chappell DC, Tsiami AA (2012) Are vegetarians susceptible to primary cardiovascular disease, due to a lack or absence of dietary animal produce? Proc Nutr Soc 71 (OCE3):E241. doi:http://dx.doi.org/10.1017/S0029665112003321
19. O'Mullan A (2019) Can a wholefood plant-based diet affect healthy ageing? Age and Ageing Conference: 67th Annual and Scientific Meeting of the Irish Gerontological Society, Innovation, Advances and Excellence in Ageing Ireland 48 (Suppl 3):iii17–iii65. doi:http://dx.doi.org/10.1093/ageing/afz103.38
20. Oliveira ERA, Velten APC, Cade NV, De Oliveira CS (2013) Comparison of seventh day adventist mortality with a general population in the Espirito Santo state, Brazil. Eur J Epidemiol 28 (1):S204-S205. doi:http://dx.doi.org/10.1007/s10654-013-9820-0
21. Ponzio E, Mazzarini G, Gasperi G, Bottoni MC, Vallorani S (2015) The Vegetarian Habit in Italy: Prevalence and Characteristics of Consumers. Ecol Food Nutr 54 (4):370-379. doi:10.1080/03670244.2014.1001981
22. Yang S-Y, Li X-J, Zhang W, Liu C-Q, Zhang H-J, Lin J-R, Yan B, Yu Y-X, Shi X-L, Li C-D, Li W-H (2012) Chinese lacto-vegetarian diet exerts favorable effects on metabolic parameters, intima-media thickness, and cardiovascular risks in healthy men. Nutrition in clinical practice : official publication of the American Society for Parenteral and Enteral Nutrition 27 (3):392-398. doi:10.1177/0884533611436173
23. Löwik MR, Schrijver J, Odink J, van den Berg H, Wedel M (1990) Long-term effects of a vegetarian diet on the nutritional status of elderly people (Dutch Nutrition Surveillance System). J Am Coll Nutr 9 (6):600-609. doi:10.1080/07315724.1990.10720415
24. Kwok TK, Woo J, Ho S, Sham A (2000) Vegetarianism and ischemic heart disease in older Chinese women. J Am Coll Nutr 19 (5):622-627. doi:10.1080/07315724.2000.10718960
25. Gupta R (1996) Lifestyle risk factors and coronary heart disease prevalence in Indian men. The Journal of the Association of Physicians of India 44 (10):689-693
26. Kapoor D, Iqbal R, Singh K, Jaacks LM, Shivashankar R, Sudha V, Anjana RM, Kadir M, Mohan V, Ali MK, Narayan KM, Tandon N, Prabhakaran D, Merchant AT (2018) Association of dietary patterns and dietary diversity with cardiometabolic disease risk factors among adults in South Asia: The CARRS study. Asia Pac J Clin Nutr 27 (6):1332-1343. doi:http://dx.doi.org/10.6133/apjcn.201811_27%286%29.0021
27. Key TJ, Fraser GE, Thorogood M, Appleby PN, Beral V, Reeves G, Burr ML, Chang-Claude J, Frentzel-Beyme R, Kuzma JW, Mann J, McPherson K (1998) Mortality in vegetarians and non-vegetarians: a collaborative analysis of 8300 deaths among 76,000 men and women in five prospective studies. Public Health Nutr 1 (1):33-41. doi:10.1079/phn19980006
28. Frentzel-Beyme R, Claude J, Eilber U (1988) Mortality among German vegetarians: first results after five years of follow-up. Nutr Cancer 11 (2):117-126. doi:10.1080/01635588809513978
29. Chang-Claude J, Frentzel-Beyme R (1993) Dietary and lifestyle determinants of mortality among German vegetarians. Int J Epidemiol 22 (2):228-236. doi:10.1093/ije/22.2.228
30. Mann JI, Appleby PN, Key TJ, Thorogood M (1997) Dietary determinants of ischaemic heart disease in health conscious individuals. Heart (British Cardiac Society) 78 (5):450-455. doi:10.1136/hrt.78.5.450
31. Key TJ, Appleby PN, Davey GK, Allen NE, Spencer EA, Travis RC (2003) Mortality in British vegetarians: review and preliminary results from EPIC-Oxford. The American journal of clinical nutrition 78 (3 Suppl):533S-538S. doi:10.1093/ajcn/78.3.533S
32. Beeson WL, Mills PK, Phillips RL, Andress M, Fraser GE (1989) Chronic disease among Seventh-day Adventists, a low-risk group. Rationale, methodology, and description of

the population. Cancer 64 (3):570-581. doi:10.1002/1097-0142(19890801)64:3<570::aid-cncr2820640303>3.0.co;2-4
33. Crowe FL, Appleby PN, Travis RC, Key TJ (2013) Risk of hospitalization or death from ischemic heart disease among British vegetarians and nonvegetarians: results from the EPIC-Oxford cohort study. The American journal of clinical nutrition 97 (3):597-603. doi:10.3945/ajcn.112.044073
34. Key TJ, Thorogood M, Appleby PN, Burr ML (1996) Dietary habits and mortality in 11,000 vegetarians and health conscious people: results of a 17 year follow up. BMJ (Clinical research ed) 313 (7060):775-779. doi:10.1136/bmj.313.7060.775
35. Burr ML, Butland BK (1988) Heart disease in British vegetarians. The American journal of clinical nutrition 48 (3 Suppl):830-832. doi:10.1093/ajcn/48.3.830
36. Burr ML, Sweetnam PM (1982) Vegetarianism, dietary fiber, and mortality. The American journal of clinical nutrition 36 (5):873-877. doi:10.1093/ajcn/36.5.873
37. Key TJ, Appleby PN, Spencer EA, Travis RC, Roddam AW, Allen NE (2009) Mortality in British vegetarians: results from the European Prospective Investigation into Cancer and Nutrition (EPIC-Oxford). The American journal of clinical nutrition 89 (5):1613S-1619S. doi:10.3945/ajcn.2009.26736L
38. Thorogood M, Mann J, Appleby P, McPherson K (1994) Risk of death from cancer and ischaemic heart disease in meat and non-meat eaters. BMJ (Clinical research ed) 308 (6945):1667-1670. doi:10.1136/bmj.308.6945.1667
39. Phillips RL, Lemon FR, Beeson WL, Kuzma JW (1978) Coronary heart disease mortality among Seventh-Day Adventists with differing dietary habits: a preliminary report. The American journal of clinical nutrition 31 (10 Suppl):S191-S198. doi:10.1093/ajcn/31.10.S191
40. Mahase E (2019) Vegetarian and pescatarian diets are linked to lower risk of ischaemic heart disease, study finds. BMJ (Clinical research ed) 366: l5397. doi:10.1136/bmj.l5397
41. Sievenpiper JL, Lavie CJ (2018) Dietary prevention of cardiovascular diseases. Prog Cardiovasc Dis 61 (1):1-2. doi:http://dx.doi.org/10.1016/j.pcad.2018.05.001
42. Stiefelhagen P (2013) [Meat free diet increases longevity in men]. [German] (Fleischverzicht lasst Manner langer leben.). MMW Fortschritte der Medizin 155 (12):7
43. Hauner H (2015) Vegetarian/vegan diet - reasonable or dangerous? (Für wen sinnvoll, für wen gefährlich?). MMW Fortschritte der Medizin 157 (10):41-43. doi:10.1007/s15006-015-3134-y
44. Shiwani AH, Aziz A, Shiwani MH (2007) Shall we become vegetarian to minimize the risk of coronary heart disease? JPMA The Journal of the Pakistan Medical Association 57 (1):46-48
45. Esselstyn Jr CB (2007) Letters from the global literature: Unresolved issues and responses [4]. MedGenMed Medscape General Medicine 9 (3):57
46. Lamisse F (2013) Vegetarian diet. [French] (L'alimentation vegetarienne.). Medecine des Maladies Metaboliques 7 (2):109-113. doi:http://dx.doi.org/10.1016/S1957-2557%2813%2970506-6
47. Sargsyan VD, Safaryan AS, Kamyshova TV (2020) Ornish diet. Whole plant-based diet for cardiovascular disease prevention. [Russian]. Profilakticheskaya Meditsina 23 (6):114-119. doi:http://dx.doi.org/10.17116/profmed202023061114
48. Brom B (2009) Integrative approach to management of heart disease is heart disease reversible? South African Family Practice 51 (3):196-197. doi:http://dx.doi.org/10.1080/20786204.2009.10873846
49. Dauchet L, Jung YJ (2019) Association between vegetarian diets and chronic diseases: An epidemiological approach (Association entre regimes vegetariens et pathologies

chroniques : une approche epidemiologique.). Cahiers de Nutrition et de Dietetique 54 (6):318-325. doi:http://dx.doi.org/10.1016/j.cnd.2019.07.004
50. Kwok CS, Mamas MA, Loke YK (2015) Vegetarian diets and Adventists. Int J Cardiol 190:383-383. doi:10.1016/j.ijcard.2015.03.392
51. Spiller GA, Bruce B (1998) Vegan diets and cardiovascular health. J Am Coll Nutr 17 (5):407-408. doi:10.1080/07315724.1998.10718786
52. Williams KA, Sr., Patel H (2017) Healthy Plant-Based Diet: What Does it Really Mean? J Am Coll Cardiol 70 (4):423-425. doi:10.1016/j.jacc.2017.06.006
53. Ros E (2018) Editorial commentary: Plant-based diets: More than meets the eye. Trends Cardiovasc Med 28 (7):442-444. doi:http://dx.doi.org/10.1016/j.tcm.2018.04.008
54. Spence JD, Tangney C (2020) Lower risk of stroke with a vegetarian diet. Neurology 94 (11):463-464. doi:10.1212/wnl.0000000000009092
55. Hickner J (2014) Can a plant-based diet really reverse CAD? J Fam Pract 63 (9):490
56. Silverstein DA (2014) Are these CAD study findings too good to be true? The Journal of family practice 63 (9):492-493
57. Mann J (2009) Vegetarian diets. BMJ (Online) 339 (7720):525. doi:http://dx.doi.org/10.1136/bmj.b2507
58. Lindbloom EJ (2009) Long-term benefits of a vegetarian diet. Am Fam Physician 79 (7):541-542
59. Fraser G (2003) Commentary: Protection from stroke by eating animal foods? Surely not! Int J Epidemiol 32 (4):543-545. doi:10.1093/ije/dyg217
60. Ellis FR, Sanders TA (1977) Angina and vegan diet. Am Heart J 93 (6):803-805. doi:10.1016/s0002-8703(77)80080-x
61. Blankenhorn DH (1985) Two new diet-heart studies. The New England journal of medicine 312 (13):851-853. doi:10.1056/NEJM198503283121309
62. Sullivan JL (1983) Vegetarianism, ischemic heart disease, and iron. The American journal of clinical nutrition 37 (5):882-883. doi:10.1093/ajcn/37.5.882
63. Ornish D (2009) Mostly plants. The American journal of cardiology 104 (7):957-958. doi:10.1016/j.amjcard.2009.05.031
64. Fields H, Millstine D, Agrwal N (2014) Just plants? Impact of a vegetarian diet on mortality. J Womens Health (Larchmt) 23 (11):987-988. doi:10.1089/jwh.2014.4972
65. Fenton T, Gillis C (2019) Plant-based diets do not prevent most chronic diseases. Crit Rev Food Sci Nutr 59 (7):1044-1045. doi:10.1080/10408398.2017.1389692
66. Estruch R, Sacanella E, Ros E (2021) Should we all go pesco-vegetarian? Eur Heart J 42 (12):1144-1146. doi:10.1093/eurheartj/ehaa1088
67. Ellis FR, Sanders T (1976) Letter: Angina and vegetarian diet. Lancet (London, England) 1 (7970):1190-1190. doi:10.1016/s0140-6736(76)91589-0
68. Siguel E, MacBeath BR, Lerman RH, Gould KL, Ornish D (1996) Very low-fat diets for coronary heart disease: Perhaps, but which one? J Am Med Assoc 275 (18):1402-1403. doi:http://dx.doi.org/10.1001/jama.275.18.1402
69. Taylor J (2013) Vegetarian diet cuts risk of heart disease by one-third. Eur Heart J 34 (21):1531-1531
70. Rajaram S, Sabate J (2009) American Journal of Clinical Nutrition: Preface. Am J Clin Nutr 89 (5):1541S-1542S. doi:http://dx.doi.org/10.3945/ajcn.2009.26736A
71. Vandenbroucke JP (1994) Should you eat meat, or are you confounded by methodological debate? BMJ (Clinical research ed) 308 (6945):1671-1671. doi:10.1136/bmj.308.6945.1671
72. Da Graca S (2019) Revue médicale suisse (Risque accru d'AVC chez les vegetariens et vegans ?.). Rev Med Suisse 15 (675):2308

73. Ha V, de Souza RJ (2015) "Fleshing Out" the Benefits of Adopting a Vegetarian Diet. Journal of the American Heart Association 4 (10):e002654-e002654. doi:10.1161/JAHA.115.002654
74. Lawrence MA, McNaughton SA (2019) Vegetarian diets and health. BMJ (Clinical research ed) 366:l5272. doi:10.1136/bmj.l5272
75. Chareonrungrueangchai K, Wongkawinwoot K, Anothaisintawee T, Reutrakul S (2020) Dietary Factors and Risks of Cardiovascular Diseases: An Umbrella Review. Nutrients 12 (4): 1088. doi:10.3390/nu12041088
76. Jafari S, Hezaveh E, Jalilpiran Y, Jayedi A, Wong A, Safaiyan A, Barzegar A (2021) Plant-based diets and risk of disease mortality: a systematic review and meta-analysis of cohort studies. Crit Rev Food Sci Nutr:1-13. doi:10.1080/10408398.2021.1918628
77. Glenn AJ, Viguiliouk E, Seider M, Boucher BA, Khan TA, Blanco Mejia S, Jenkins DJA, Kahleová H, Rahelić D, Salas-Salvadó J, Kendall CWC, Sievenpiper JL (2019) Relation of Vegetarian Dietary Patterns With Major Cardiovascular Outcomes: A Systematic Review and Meta-Analysis of Prospective Cohort Studies. Front Nutr 6:80. doi:10.3389/fnut.2019.00080
78. Huang T, Yang B, Zheng J, Li G, Wahlqvist ML, Li D (2012) Cardiovascular disease mortality and cancer incidence in vegetarians: a meta-analysis and systematic review. Ann Nutr Metab 60 (4):233-240. doi:10.1159/000337301
79. Kwok CS, Umar S, Myint PK, Mamas MA, Loke YK (2014) Vegetarian diet, Seventh Day Adventists and risk of cardiovascular mortality: a systematic review and meta-analysis. Int J Cardiol 176 (3):680-686. doi:10.1016/j.ijcard.2014.07.080
80. Oussalah A, Levy J, Berthezène C, Alpers DH, Guéant JL (2020) Health outcomes associated with vegetarian diets: An umbrella review of systematic reviews and meta-analyses. Clin Nutr 39 (11):3283-3307. doi:10.1016/j.clnu.2020.02.037
81. Dinu M, Abbate R, Gensini GF, Casini A, Sofi F (2017) Vegetarian, vegan diets and multiple health outcomes: A systematic review with meta-analysis of observational studies. Crit Rev Food Sci Nutr 57 (17):3640-3649. doi:10.1080/10408398.2016.1138447
82. Fraser GE (2005) A comparison of first event coronary heart disease rates in two contrasting California populations. J Nutr Health Aging 9 (1):53-58
83. Kinlen LJ, Hermon C, Smith PG (1983) A proportionate study of cancer mortality among members of a vegetarian society. Br J Cancer 48 (3):355-361. doi:10.1038/bjc.1983.200
84. Chang-Claude J, Frentzel-Beyme R, Eilber U (1992) Mortality pattern of German vegetarians after 11 years of follow-up. Epidemiology (Cambridge, Mass) 3 (5):395-401. doi:10.1097/00001648-199209000-00003
85. Snowdon DA (1988) Animal product consumption and mortality because of all causes combined, coronary heart disease, stroke, diabetes, and cancer in Seventh-day Adventists. The American journal of clinical nutrition 48 (3 Suppl):739-748. doi:10.1093/ajcn/48.3.739
86. Chen C, Chaudhary A, Mathys A (2019) Dietary change scenarios and implications for environmental, nutrition, human health and economic dimensions of food sustainability. Nutrients 11 (4):856. doi:http://dx.doi.org/10.3390/nu11040856
87. Fraser GE, Shavlik DJ (1997) Risk factors for all-cause and coronary heart disease mortality in the oldest-old. The Adventist Health Study. Arch Intern Med 157 (19):2249-2258
88. Alshahrani SM, Fraser GE, Sabaté J, Knutsen R, Shavlik D, Mashchak A, Lloren JI, Orlich MJ (2019) Red and Processed Meat and Mortality in a Low Meat Intake Population. Nutrients 11 (3):622. doi:10.3390/nu11030622

89. Aston LM, Smith JN, Powles JW (2012) Impact of a reduced red and processed meat dietary pattern on disease risks and greenhouse gas emissions in the UK: a modelling study. BMJ Open 2 (5):e001072. doi:10.1136/bmjopen-2012-001072
90. Baden MY, Liu G, Satija A, Li Y, Sun Q, Fung TT, Rimm EB, Willett WC, Hu FB, Bhupathiraju SN (2019) Association of changes in plant-based diet indices with total and cause-specific mortality [abstract]. Circulation Conference: American Heart Association's Epidemiology and Prevention/Lifestyle and Cardiometabolic Health 139 (Supplement 1):AMP38. doi:http://dx.doi.org/10.1161/circ.139.suppl_1.MP38
91. Baden MY, Liu G, Satija A, Li Y, Sun Q, Fung TT, Rimm EB, Willett WC, Hu FB, Bhupathiraju SN (2019) Changes in Plant-Based Diet Quality and Total and Cause-Specific Mortality. Circulation 140 (12): 979-991. doi:10.1161/CIRCULATIONAHA.119.041014
92. Beckman JA, Shibao CA (2020) Trimethylamine-N-Oxide, More Red Meat for the Vascular Scientists. Hypertension 76 (1):40-41. doi:http://dx.doi.org/10.1161/HYPERTENSIONAHA.120.14857
93. Beilin LJ (1992) Strategies and difficulties in dietary intervention in myocardial infarction patients. Clinical and experimental hypertension Part A, Theory and practice 14 (1-2):213-221. doi:10.3109/10641969209036183
94. Berkel J, de Waard F (1983) Mortality pattern and life expectancy of Seventh-Day Adventists in the Netherlands. Int J Epidemiol 12 (4):455-459. doi:10.1093/ije/12.4.455
95. Böni R (2016) What is the Optimal Nutrition for Humans? (Was ist die optimale Ernährung für den Menschen?). Praxis 105 (9):517-521. doi:10.1024/1661-8157/a002348
96. Campbell TC (2017) A plant-based diet and animal protein: Questioning dietary fat and considering animal protein as the main cause of heart disease. Journal of Geriatric Cardiology 14 (5):331-337. doi:http://dx.doi.org/10.11909/j.issn.1671-5411.2017.05.011
97. Cena H, Calder PC (2020) Defining a healthy diet: Evidence for the role of contemporary dietary patterns in health and disease. Nutrients 12 (2):334. doi:http://dx.doi.org/10.3390/nu12020334
98. Christiansen NS, Schmidt AW, Johansen C, Ross L, Hvidt NC, Thygesen LC (2015) Risk for cardiovascular disease among Seventh-day Adventists and Baptists in Denmark, 1977-2009. Int J Cardiol 185: 133-135. doi:10.1016/j.ijcard.2015.03.131
99. Da Rosa EM, Scatola RP, Possa R (2008) Cardiovascular risk in vegetarians and omnivores: A comparative study. [Portuguese, English] (Risco cardiovascular em vegetarianos e onivoros: Um estudo comparativo.). Arq Bras Cardiol 91 (4):287. doi:http://dx.doi.org/10.1590/S0066-782X2008001600013
100. Farad-Bensenouci S, Lamisse F (1997) Vegetarian diet, hypertension and coronary heart disease. [French] (Alimentation vegetarienne et maladies cardiovasculaires.). Cahiers de Nutrition et de Dietetique 32 (4):261-266
101. Fønnebø V (1985) The Tromsø Heart Study: coronary risk factors in Seventh-Day Adventists. Am J Epidemiol 122 (5):789-793. doi:10.1093/oxfordjournals.aje.a114162
102. Fønnebø V (1992) Mortality in Norwegian Seventh-Day Adventists 1962-1986. J Clin Epidemiol 45 (2):157-167. doi:10.1016/0895-4356(92)90008-b
103. Fønnebø V (1994) The healthy Seventh-Day Adventist lifestyle: what is the Norwegian experience? The American journal of clinical nutrition 59 (5 Suppl):1124S-1129S. doi:10.1093/ajcn/59.5.1124S
104. Foscolou A, Critselis E, Tyrovolas S, Chrysohoou C, Rallidis L, Matalas AL, Sidossis LS, Panagiotakos D (2020) Plant and animal protein consumption, cardiometabolic risk and healthy aging: Attica and medis epidemiological studies. Atherosclerosis 315:e100-e101. doi:http://dx.doi.org/10.1016/j.atherosclerosis.2020.10.310

105. Fraser GE, Sabaté J, Beeson WL (1993) The application of results of some studies of California Seventh-Day Adventists to the general population. Arch Intern Med 153 (4):533-534
106. Fraser GE, Sabaté J, Beeson WL, Strahan TM (1992) A possible protective effect of nut consumption on risk of coronary heart disease. The Adventist Health Study. Arch Intern Med 152 (7):1416-1424
107. Fraser GE, Strahan TM, Sabaté J, Beeson WL, Kissinger D (1992) Effects of traditional coronary risk factors on rates of incident coronary events in a low-risk population. The Adventist Health Study. Circulation 86 (2):406-413. doi:10.1161/01.cir.86.2.406
108. Fresán U, Martínez-González MA, Sabaté J, Bes-Rastrollo M (2019) Global sustainability (health, environment and monetary costs) of three dietary patterns: results from a Spanish cohort (the SUN project). BMJ Open 9 (2):e021541-e021541. doi:10.1136/bmjopen-2018-021541
109. Ha B (2019) The Power of Plants: Is a Whole-Foods, Plant-Based Diet the Answer to Health, Health Care, and Physician Wellness? Perm J 23: 19-003. doi:10.7812/tpp/19.003
110. Halvorsen BA, Vellar OD, Svendsen B (1985) Cancer and cardiovascular mortality rates among Seventh-day adventists. Are reduced rates effect of better lifestyle?. [Norwegian]. Tidsskr Nor Laegeforen 105 (24):1620-1625+1630
111. Halvorsen BA, Vellar OD, Svendsen B (1985) Reduced mortality of cancer and cardiovascular diseases among Seventh Day Adventists. Health benefits of better life style? (Lavere dødelighet av kreft og hjerte- og karsykdommer hos adventister. Helsegevinster ved bedre livsstil?). Tidsskrift for den Norske laegeforening : tidsskrift for praktisk medicin, ny raekke 105 (24):1620-1625
112. Heianza Y, Zhou T, Sun D, Hu FB, Manson JE, Qi L (2020) Genetic susceptibility, plant-based dietary patterns, and risk of cardiovascular disease. Am J Clin Nutr 112 (1):220-228. doi:http://dx.doi.org/10.1093/ajcn/nqaa107
113. Heianza Y, Zhou T, Sun D, Hu FB, Qi L (2021) Healthful plant-based dietary patterns, genetic risk of obesity, and cardiovascular risk in the UK biobank study. Clin Nutr 40 (7):4694-4701. doi:http://dx.doi.org/10.1016/j.clnu.2021.06.018
114. Heianza Y, Zhou T, Sun D, Qi L (2019) Genetic predisposition to obesity and healthful plant-based diet in risks of hypertension and cardiovascular disease: Gene-diet interaction analyses in the UK biobank. Circulation Conference: American Heart Association's Epidemiology and Prevention/Lifestyle and Cardiometabolic Health 139 (Supplement 1):AMP05. doi:http://dx.doi.org/10.1161/circ.139.suppl_1.MP05
115. Heidemann C, Schulze MB, Franco OH, Van Dam RM, Mantzoros CS, Hu FB (2008) Dietary patterns and risk of mortality from cardiovascular disease, cancer, and all causes in a prospective cohort of women. Circulation 118 (3):230-237. doi:http://dx.doi.org/10.1161/CIRCULATIONAHA.108.771881
116. Hirayama T (1985) Mortality in Japanese with life-styles similar to Seventh-Day Adventists: strategy for risk reduction by life-style modification. National Cancer Institute monograph 69:143-153
117. Hoffmann K, Zyriax B-C, Boeing H, Windler E (2004) A dietary pattern derived to explain biomarker variation is strongly associated with the risk of coronary artery disease. The American journal of clinical nutrition 80 (3):633-640. doi:10.1093/ajcn/80.3.633
118. Karagodin VP, Sukhorukov VN, Orekhov AN, Yet SF, Sobenin IA (2020) Atherosclerosis prevention: the role of special diets and functional food. Front Biosci (Elite Ed) 12:95-101. doi:10.2741/e859
119. Kim H, Caulfield LE, Garcia-Larsen V, Steffen LM, Coresh J, Rebholz CM (2019) Plant-Based Diets Are Associated With a Lower Risk of Incident Cardiovascular Disease, Cardiovascular Disease Mortality, and All-Cause Mortality in a General Population of

Middle-Aged Adults. Journal of the American Heart Association 8 (16): e012865. doi:http://dx.doi.org/10.1161/JAHA.119.012865
120. Kim H, Caulfield LE, Garcia-Larsen V, Steffen LM, Coresh J, Rebholz CM (2019) Plant-based diets are associated with a lower risk of cardiovascular disease, cardiovascular disease mortality, and all-cause mortality. Circulation Conference: American Heart Association's Epidemiology and Prevention/Lifestyle and Cardiometabolic Health 139 (Supplement 1):216. doi:http://dx.doi.org/10.1161/circ.139.suppl_1.P216
121. Kim H, Caulfield LE, Garcia-Larsen V, Steffen LM, Coresh J, Rebholz CM (2019) Plant-Based Diets Are Associated With a Lower Risk of Incident Cardiovascular Disease, Cardiovascular Disease Mortality, and All-Cause Mortality in a General Population of Middle-Aged Adults. J Am Heart Assoc 8 (16):e012865. doi:10.1161/jaha.119.012865
122. Kim H, Caulfield LE, Rebholz CM (2018) Healthy Plant-Based Diets Are Associated with Lower Risk of All-Cause Mortality in US Adults. The Journal of nutrition 148 (4):624-631. doi:10.1093/jn/nxy019
123. Kim K (2019) Semi-vegetarians' body fat indices, metabolic risks and cardiovascular disease in Koreans. Obesity Facts 12 (Supplement 1):153. doi:http://dx.doi.org/10.1159/000489691
124. Kugelberg E (2013) Nutrition: Red meat consumption leads to a microbiota-dependent risk of cardiovascular disease. Nature Reviews Endocrinology 9 (7):378. doi:http://dx.doi.org/10.1038/nrendo.2013.90
125. Lamisse F (2006) Is it necessary to be a 7th day Adventist for a good health?. [French] (Faut-il etre Adventiste du 7^e jour pour etre en bonne sante?.). Cahiers de Nutrition et de Dietetique 41 (6):347-351
126. Lassale C, Beulens J, Van Der Schouw Y, Roswall N, Weiderpass E, Romaguera D, Riboli E, Tzoulaki I (2015) A pro-vegetarian food pattern and cardiovascular mortality in the epic study. Circulation Conference: American Heart Association's Epidemiology and Prevention/Lifestyle and Cardiometabolic Health 131 (SUPPL. 1):A16
127. Lerman RH (2010) The macrobiotic diet in chronic disease. Nutrition in clinical practice : official publication of the American Society for Parenteral and Enteral Nutrition 25 (6):621-626. doi:10.1177/0884533610385704
128. Lichtenstein AH, Van Horn L (1998) Very low fat diets. Circulation 98 (9):935-939. doi:10.1161/01.cir.98.9.935
129. Lockheart MSK, Steffen LM, Rebnord HM, Fimreite RL, Ringstad J, Thelle DS, Pedersen JI, Jacobs DR, Jr. (2007) Dietary patterns, food groups and myocardial infarction: a case-control study. The British journal of nutrition 98 (2):380-387. doi:10.1017/S0007114507701654
130. Mangat I (2009) Do vegetarians have to eat fish for optimal cardiovascular protection? The American journal of clinical nutrition 89 (5):1597S-1601S. doi:10.3945/ajcn.2009.26736I
131. Martínez-González MA, Sánchez-Tainta A, Corella D, Salas-Salvadó J, Ros E, Arós F, Gómez-Gracia E, Fiol M, Lamuela-Raventós RM, Schröder H, Lapetra J, Serra-Majem L, Pinto X, Ruiz-Gutierrez V, Estruch R, Group P (2014) A provegetarian food pattern and reduction in total mortality in the Prevención con Dieta Mediterránea (PREDIMED) study. The American journal of clinical nutrition 100 Suppl 1:320S-328S. doi:10.3945/ajcn.113.071431
132. McCarty MF (2004) A taurine-supplemented vegan diet may blunt the contribution of neutrophil activation to acute coronary events. Med Hypotheses 63 (3):419-425. doi:10.1016/j.mehy.2004.03.040

133. Mendoza-Vasconez AS, Landry MJ, Crimarco A, Bladier C, Gardner CD (2021) Sustainable Diets for Cardiovascular Disease Prevention and Management. Current Atherosclerosis Reports 23 (7):31. doi:http://dx.doi.org/10.1007/s11883-021-00929-0
134. Michgelsen J, Boateng D, Meeks KAC, Beune E, Addo J, Bahendeka S, Stronks K, Agyemang C (2021) Association between practising religion and cardiovascular disease risk among Ghanaian non-migrants and migrants in Europe: The rodam study. Int J Environ Res Public Health 18 (5):1-14. doi:http://dx.doi.org/10.3390/ijerph18052451
135. Modi GK (2010) Masala. Natl Med J India 23 (4):255
136. Montgomery S, Herring P, Yancey A, Beeson L, Butler T, Knutsen S, Sabate J, Chan J, Preston-Martin S, Fraser G (2007) Comparing self-reported disease outcomes, diet, and lifestyles in a national cohort of black and white Seventh-day Adventists. Prev Chronic Dis 4 (3):A62-A62
137. Morin É, Michaud-Létourneau I, Couturier Y, Roy M (2019) A whole-food, plant-based nutrition program: Evaluation of cardiovascular outcomes and exploration of food choices determinants. Nutrition 66:54-61. doi:10.1016/j.nut.2019.03.020
138. Murphy FG, Blumenthal DS, Dickson-Smith J, Peay RP (1990) The mortality profile of black Seventh-Day Adventists residing in metropolitan Atlanta: a pilot study. Am J Public Health 80 (8): 984–985. doi:10.2105/ajph.80.8.984
139. Naghipour S, Cox AJ, Peart JN, Du Toit EF, Headrick JP (2021) Trimethylamine N-oxide: heart of the microbiota-CVD nexus? Nutr Res Rev 34 (1):125-146. doi:10.1017/s0954422420000177
140. Navarro JA, Caramelli B (2010) Vegetarians from latin america. The American journal of cardiology 105 (6):902-902. doi:10.1016/j.amjcard.2009.10.031
141. Ness AR, Powles JW (2014) The role of diet, fruit and vegetables and antioxidants in the Aetiology of stroke. European Journal of Preventive Cardiology 6 (4):229-234. doi:http://dx.doi.org/10.1177/204748739900600407
142. Ness AR, Powles JW, Morgan JF, Key TJA, Appleby PN (1997) Dietary habits and mortality in vegetarians and health conscious people (multiple letters) [9]. Br Med J 314 (7074):148-149
143. Niveditha AS, Premavathy D (2019) Prevalence of heart disease among non-vegetarian individuals. Drug Invention Today 12 (6):1250-1253
144. Oliveira ERA, Cade NV, Velten APC, Silva GAE, Faerstein E (2016) Comparative study of cardiovascular and cancer mortality of Adventists and non-Adventists from Espírito Santo State, in the period from 2003 to 2009 (Estudo comparativo da mortalidade cardiovascular e por neoplasia de Adventistas e Não Adventistas do Estado do Espírito Santo, no período de 2003 a 2009). Rev Bras Epidemiol 19 (1):112-121. doi:10.1590/1980-5497201600010010
145. Palmer BF, Clegg DJ (2019) Cardiovascular Benefits of a Diet Enriched in Fruits and Vegetables. Am J Nephrol 49 (6):435-437. doi:http://dx.doi.org/10.1159/000500044
146. Pawlak R (2015) Is vitamin B12 deficiency a risk factor for cardiovascular disease in vegetarians? Am J Prev Med 48 (6):e11-e26. doi:10.1016/j.amepre.2015.02.009
147. Phillips RL, Kuzma JW, Beeson WL, Lotz T (1980) Influence of selection versus lifestyle on risk of fatal cancer and cardiovascular disease among Seventh-day Adventists. Am J Epidemiol 112 (2):296-314. doi:10.1093/oxfordjournals.aje.a112996
148. Rao V, Al-Weshahy A (2008) Plant-based diets and control of lipids and coronary heart disease risk. Current atherosclerosis reports 10 (6):478-485. doi:10.1007/s11883-008-0075-2
149. Raposo S, Satija A, Forman J, Li S, Mukamal K, Lof M, Manson J, Hu F, Rimm E (2019) Diet quality and incidence of coronary heart disease and coronary revascularization among US women and men with hypertension. Circulation Conference: American Heart

Association's Epidemiology and Prevention/Lifestyle and Cardiometabolic Health 139 (Suppl. 1):221. doi:http://dx.doi.org/10.1161/circ.139.suppl_1.P221
150. Rathod KS, Velmurugan S, Ahluwalia A (2016) A 'green' diet-based approach to cardiovascular health? Is inorganic nitrate the answer? Mol Nutr Food Res 60 (1): 185-202. doi:10.1002/mnfr.201500313
151. Rohrmann S, Overvad K, Bueno-de-Mesquita HB, Jakobsen MU, Egeberg R, Tjønneland A, Nailler L, Boutron-Ruault M-C, Clavel-Chapelon F, Krogh V, Palli D, Panico S, Tumino R, Ricceri F, Bergmann MM, Boeing H, Li K, Kaaks R, Khaw K-T, Wareham NJ, Crowe FL, Key TJ, Naska A, Trichopoulou A, Trichopoulos D, Leenders M, Peeters PHM, Engeset D, Parr CL, Skeie G, Jakszyn P, Sánchez M-J, Huerta JM, Redondo ML, Barricarte A, Amiano P, Drake I, Sonestedt E, Hallmans G, Johansson I, Fedirko V, Romieux I, Ferrari P, Norat T, Vergnaud AC, Riboli E, Linseisen J (2013) Meat consumption and mortality--results from the European Prospective Investigation into Cancer and Nutrition. BMC Med 11 (1):1-12. doi:10.1186/1741-7015-11-63
152. Rugulies R, Jager A, Benesch L, Siegrist J (1996) Comprehensive lifestyle changes among patients with CHD and the integration of inpatient and outpatient cardiac rehabilitation: First results of a secondary prevention intervention trial. [German] (Komprehensive Lebensstilanderung bei Koronarkranken und die Integration stationarer und ambulanter Rehabilitation: Erste Ergebnisse einer sekundarpraventiven Interventionsstudie.). Zeitschrift fur Gesundheitswissenschaften 4 (3):234-247
153. Sabaté J (1993) Does nut consumption protect against ischaemic heart disease? Eur J Clin Nutr 47 Suppl 1:S71-S75
154. Sanchez A, Mejia A, Sanchez J, Runte E, Brown-Fraser S, Bivens RL (2019) Diets with customary levels of fat from plant origin may reverse coronary artery disease. Med Hypotheses 122:103-105. doi:10.1016/j.mehy.2018.10.027
155. Satija A, Bhupathiraju SN, Spiegelman D, Chiuve SE, Manson JE, Willett W, Rexrode KM, Rimm EB, Hu FB (2017) Healthful and Unhealthful Plant-Based Diets and the Risk of Coronary Heart Disease in U.S. Adults. J Am Coll Cardiol 70 (4):411-422. doi:10.1016/j.jacc.2017.05.047
156. Satija A, Bhupathiraju SN, Spiegelman D, Chiuve SE, Manson JE, Willett WC, Rexrode KM, Rimm EB, Hu FB (2017) Plant-based diets and the risk of coronary heart disease in US adults. FASEB Journal Conference: Experimental Biology 31 (1 Supplement 1)
157. Satija A, Hu FB (2018) Plant-based diets and cardiovascular health. Trends Cardiovasc Med 28 (7):437-441. doi:10.1016/j.tcm.2018.02.004
158. Satija A, Malik VS, Willett WC, Hu FB (2017) Meta-analysis of red meat intake and cardiovascular risk factors: Methodologic limitations. Am J Clin Nutr 105 (6):1567-1568. doi:http://dx.doi.org/10.3945/ajcn.117.153692
159. Schulze MB, Martinez-Gonzalez MA, Fung TT, Lichtenstein AH, Forouhi NG (2018) Food based dietary patterns and chronic disease prevention. BMJ (Online) 361 (no pagination):k2396. doi:http://dx.doi.org/10.1136/bmj.k2396
160. Shan Z, Li Y, Baden MY, Bhupathiraju SN, Wang DD, Sun Q, Rexrode KM, Rimm EB, Qi L, Willett WC, Manson JE, Qi Q, Hu FB (2020) Association between healthy eating patterns and risk of cardiovascular disease. JAMA Internal Medicine 180 (8):1090-1100. doi:http://dx.doi.org/10.1001/jamainternmed.2020.2176
161. Shan Z, Li Y, Bhupathiraju SN, Wang D, Rexrode KM, Rimm E, Qi L, Willett W, Manson JE, Qi Q, Hu FB (2020) Healthy eating patterns and risk of cardiovascular disease: Results from three large prospective cohort studies. Circulation Conference: American Heart Association's Epidemiology and Prevention/Lifestyle and Cardiometabolic Health 141 (SUPPL 1):A37. doi:http://dx.doi.org/10.1161/circ.141.suppl-1.37

162. Shiue I, Arima H, Hankey GJ, Anderson CS (2011) Dietary intake of key nutrients and subarachnoid hemorrhage: a population-based case-control study in Australasia. Cerebrovascular diseases (Basel, Switzerland) 31 (5):464-470. doi:10.1159/000324386
163. Singh RB, Visen P, Sharma D, Sharma S, Mondol R, Sharma JP, Sharma M, Tokunaga M, Takahashi T, Mishra S, Sharma A, Jain M, Marinho F, Pal R (2014) Study of functional foods consumption patterns among decedents dying due to various causes of death. Open Nutraceuticals Journal 8 (1):16-28
164. Spence JD (2013) Intensive risk factor control in stroke prevention. F1000Prime Reports 5 (no pagination). doi:http://dx.doi.org/10.12703/P5-42
165. Spence JD (2015) Letter by Spence Regarding Article, "diet and Stroke: Recent Evidence Supporting a Mediterranean-Style Diet and Food in the Primary Prevention of Stroke". Stroke 46 (9):e221. doi:http://dx.doi.org/10.1161/STROKEAHA.115.010800
166. Spence JD (2017) Nutrition in Stroke Prevention. Semin Neurol 37 (3):259-266. doi:http://dx.doi.org/10.1055/s-0037-1603470
167. Spence JD (2018) Diet for stroke prevention. Stroke Vasc Neurol 3 (2):44-50. doi:10.1136/svn-2017-000130
168. Sterling SR, Bowen S-A (2019) The Potential for Plant-Based Diets to Promote Health Among Blacks Living in the United States. Nutrients 11 (12):2915. doi:10.3390/nu11122915
169. Ströhle A, Waldmann A, Wolters M, Hahn A (2006) Vegetarian nutrition: preventive potential and possible risks. Part 2: animal foods and recommendations (Vegetarische Ernährung: Präventives Potenzial und mögliche Risiken : Teil 2: Lebensmittel tierischer Herkunft und Empfehlungen). Wien Klin Wochenschr 118 (23-24):728-737. doi:10.1007/s00508-006-0716-9
170. Ströhle A, Waldmann A, Wolters M, Hahn A (2006) Vegetarian nutrition: Preventive potential and possible risks. Part 1: Plant foods (Vegetarische Ernährung: Präventives Potenzial und mögliche Risiken. Teil 1: Lebensmittel pflanzlicher Herkunft). Wien Klin Wochenschr 118 (19-20):580-593. doi:10.1007/s00508-006-0706-y
171. Sweeney M (2004) Effects of very low-fat diets on anginal symptoms. Med Hypotheses 63 (3):553-553. doi:10.1016/j.mehy.2004.05.001
172. Tantrarongroj K, Nelson ER (1990) Incidence of first acute myocardial infarction in the Bangkok Adventist Hospital (1958-1985). Journal of the Medical Association of Thailand = Chotmaihet thangphaet 73 (1):29-34
173. Tharrey M, Mariotti F, Mashchak A, Barbillon P, Delattre M, Fraser GE (2018) Patterns of plant and animal protein intake are strongly associated with cardiovascular mortality: the Adventist Health Study-2 cohort. Int J Epidemiol 47 (5):1603-1612. doi:10.1093/ije/dyy030
174. Tuso PJ, Ismail MH, Ha BP, Bartolotto C (2013) Nutritional update for physicians: plant-based diets. Perm J 17 (2):61-66. doi:10.7812/TPP/12-085
175. Ullmann D, Phillips RL, Beeson WL, Dewey HG, Brin BN, Kuzma JW, Mathews CP, Hirst AE (1991) Cause-specific mortality among physicians with differing life-styles. JAMA 265 (18):2352-2359
176. Waaler HT, Hjort PF (1981) Longevity among Norwegian Adventists 1960-1977: a message of life style and health? (Høyere levealder hos norske adventister 1960-1977: et budskap om livsstil og helse?). Tidsskrift for den Norske laegeforening : tidsskrift for praktisk medicin, ny raekke 101 (11):623-627
177. Waaler Th H, Hjort PF (1981) Low mortality among Norwegian Seventh-Day Adventists 1960-1977: A message on lifestyle and health? [Norwegian]. Tidsskr Nor Laegeforen 101 (11):623-627+653

178. Wandera SO, Kwagala B, Ntozi J (2015) Prevalence and risk factors for self-reported non-communicable diseases among older Ugandans: a cross-sectional study. Glob Health Action 8:27923-27923. doi:10.3402/gha.v8.27923
179. Webster IW, Rawson GK (1979) Health status of Seventh-Day Adventists. The Medical journal of Australia 1 (10):417-420
180. Wierzbicki AS, Swaminathan R (2000) Coronary heart disease in Indian Asians. Lancet (London, England) 355 (9216):1727-1728. doi:10.1016/S0140-6736(05)73136-6
181. Williams KA (2017) Introduction to the "A plant-based diet and cardiovascular disease" special issue. Journal of Geriatric Cardiology 14 (5):316. doi:http://dx.doi.org/10.11909/j.issn.1671-5411.2017.05.001
182. Withnell A (2003) The natural cure of coronary heart disease. Nutr Health 17 (1):55-60. doi:http://dx.doi.org/10.1177/026010600301700106
183. Wong MMH, Louie JCY (2021) A priori dietary patterns and cardiovascular disease incidence in adult population-based studies: a review of recent evidence. Crit Rev Food Sci Nutr:1-16. doi:http://dx.doi.org/10.1080/10408398.2021.1897517
184. Wynder EL, Lemon FR (1958) Cancer, coronary artery disease and smoking: a preliminary report on differences in incidence between Seventh-day Adventists and others. Calif Med 89 (4):267-272
185. Wynder EL, Lemon FR, Bross IJ (1959) Cancer and coronary artery disease among Seventh-Day Adventists. Cancer 12:1016-1028. doi:10.1002/1097-0142(195909/10)12:5<1016::aid-cncr2820120523>3.0.co;2-2
186. Yusuf Z, Alamelu J, Lim M, Dlamini N (2015) Vitamin B12 deficiency-a poorly recognised and treatable childhood stroke risk factor. Stroke Conference: American Heart Association/American Stroke Association 46 (SUPPL. 1)
187. Zhou YF, Song XY, Wu J, Chen GC, Neelakantan N, van Dam RM, Feng L, Yuan JM, Pan A, Koh WP (2021) Association Between Dietary Patterns in Midlife and Healthy Ageing in Chinese Adults: The Singapore Chinese Health Study. J Am Med Dir Assoc 22 (6):1279-1286. doi:http://dx.doi.org/10.1016/j.jamda.2020.09.045
188. Ferraro RA, Fischer NM, Xun H, Michos ED (2020) Nutrition and physical activity recommendations from the United States and European cardiovascular guidelines: a comparative review. Curr Opin Cardiol 35 (5):508-516. doi:10.1097/hco.0000000000000763
189. Fraser GE (1999) Diet as primordial prevention in Seventh-Day Adventists. Prev Med 29 (6 II):S18-S23. doi:http://dx.doi.org/10.1006/pmed.1998.0415
190. Fraser GE (1999) Associations between diet and cancer, ischemic heart disease, and all-cause mortality in non-Hispanic white California Seventh-day Adventists. The American journal of clinical nutrition 70 (3 Suppl):532S-538S. doi:10.1093/ajcn/70.3.532s
191. Jenkins DJA, Kendall CWC, Marchie A, Jenkins AL, Connelly PW, Jones PJH, Vuksan V (2003) The Garden of Eden - Plant based diets, the genetic drive to conserve cholesterol and its implications for heart disease in the 21st century. Comparative Biochemistry and Physiology - A Molecular and Integrative Physiology 136 (1):141-151. doi:http://dx.doi.org/10.1016/S1095-6433%2802%2900345-8
192. Lecerf J-M (2011) Meat and human health: excess and errors (Viande et santé humaine: excès et défauts). Bull Acad Natl Med 195 (8):1801-1812
193. Navarro JCA, Prado SMC, Cárdenas PA, Santos RD, Caramelli B (2010) Pre-historic eating patterns in Latin America and protective effects of plant-based diets on cardiovascular risk factors. Clinics (Sao Paulo) 65 (10):1049-1054. doi:10.1590/s1807-59322010001000022
194. Neuhouser ML (2019) The importance of healthy dietary patterns in chronic disease prevention. Nutr Res 70:3-6. doi:http://dx.doi.org/10.1016/j.nutres.2018.06.002

195. Patel H, Chandra S, Alexander S, Soble J, Williams KA, Sr. (2017) Plant-Based Nutrition: An Essential Component of Cardiovascular Disease Prevention and Management. Curr Cardiol Rep 19 (10):104-104. doi:10.1007/s11886-017-0909-z
196. Samuel A, Hyman S (2004) Plants: Diet and health. Nutr Bull 29 (2):156-159
197. Meta-analysis sows doubt. Vegetarians live healthier lives but is it the food? (2014) (Metaanalyse sät Zweifel. Vegetarier leben gesünder aber liegt es am Essen?). MMW Fortschritte der Medizin 156 Spec no 2:7-7
198. Alrabadi NI (2012) The effect of lifestyle food on chronic diseases: a comparison between vegetarians and non-vegetarians in Jordan. Global journal of health science 5 (1):65-69. doi:10.5539/gjhs.v5n1p65
199. Anderson CAM (2018) Dietary Patterns to Reduce Weight and Optimize Cardiovascular Health: Persuasive Evidence for Promoting Multiple, Healthful Approaches. Circulation 137 (11):1114-1116. doi:10.1161/CIRCULATIONAHA.117.031429
200. Barnard ND, Scherwitz LW, Ornish D (1992) Adherence and acceptability of a low-fat, vegetarian diet among patients with cardiac disease. J Cardiopulm Rehabil 12 (6):423-431. doi:http://dx.doi.org/10.1097/00008483-199211000-00009
201. Caspermeyer J (2016) Are We What We Eat? Evidence of a Vegetarian Diet Permanently Shaping the Human Genome to Change Individual Risk of Cancer and Heart Disease. Mol Biol Evol 33 (7):1887-1888. doi:10.1093/molbev/msw076
202. Fraser GE, Orlich MJ, Jaceldo-Siegl K (2015) Studies of chronic disease in Seventh-day Adventists. Int J Cardiol 184:573-573. doi:10.1016/j.ijcard.2015.03.015
203. Fresan U, Craig WJ, Martinez-Gonzalez MA, Bes-Rastrollo M (2020) Nutritional quality and health effects of low environmental impact diets: The "seguimiento universidad de navarra" (sun) Cohort. Nutrients 12 (8):1-19. doi:http://dx.doi.org/10.3390/nu12082385
204. Fugar S, Fughhi IA, McDaniel R, Gates S, Sawyer S, Mason T, Williams KA (2019) Impact of a plant-based nutrition intervention on the 10-year ascvd risk in African Americans. Circulation Conference: American Heart Association Scientific Sessions, AHA 140 (Suppl 1):A16318. doi:http://dx.doi.org/10.1161/circ.140.suppl_1.16318
205. Harvey T, Smillie L, Ikin A, Farshid A (2020) 753 The Effect of a Plant-Based Diet on Cardiovascular Risk Factors in Patients With Ischaemic Heart Disease. Heart Lung and Circulation 29 (Supplement 2):S375. doi:http://dx.doi.org/10.1016/j.hlc.2020.09.760
206. Jakše B, Jakše B, Godnov U, Pinter S (2021) Nutritional, Cardiovascular Health and Lifestyle Status of 'Health Conscious' Adult Vegans and Non-Vegans from Slovenia: A Cross-Sectional Self-Reported Survey. Int J Environ Res Public Health 18 (11):5968. doi:10.3390/ijerph18115968
207. Kim HS, Rotundo L, Deutsch J, Kothari N, Ahlawat S, Demyen M, Pyrsopoulos N (2016) Obesity and cardiovascular disease risk in vegetarians in the United States: A population-based study. Am J Gastroenterol 111 (Supplement 1):S433. doi:http://dx.doi.org/10.1038/ajg.2016.360
208. Liao C-M, Lin C-M (2018) Life Course Effects of Socioeconomic and Lifestyle Factors on Metabolic Syndrome and 10-Year Risk of Cardiovascular Disease: A Longitudinal Study in Taiwan Adults. Int J Environ Res Public Health 15 (10):2178. doi:10.3390/ijerph15102178
209. Lopes T, Zemlin AE, Erasmus RT, Faber M, Kengne AP (2020) Assessment of the association of plant-based diets with cardiovascular disease risk profile in Africa: a systematic review and meta-analysis protocol. BMJ Open 10 (6): e036792. doi:10.1136/bmjopen-2020-036792
210. Rees K, Al-Khudairy L, Takeda A, Stranges S (2021) Vegan dietary pattern for the primary and secondary prevention of cardiovascular diseases. Cochrane Database Syst Rev 2 (2):Cd013501. doi:10.1002/14651858.CD013501.pub2

211. Rosa EMd, Scatola RP, Possa R (2008) Cardiovascular risk in vegetarians and omnivores: a comparative study. Arq Bras Cardiol 91 (4):263-288. doi:10.1590/s0066-782x2008001600013
212. Roy S, Gupta A, Chawla K, Tsigoulis M, Verma S, Gupta MK (2011) Prevalence of risk factors and occult coronary disease in South Asians referred to a specialized risk assessment clinic. Can J Cardiol 27 (5):S68-S69. doi:http://dx.doi.org/10.1016/j.cjca.2011.07.033
213. Sebastiani G, Herranz Barbero A, Borrás-Novell C, Alsina Casanova M, Aldecoa-Bilbao V, Andreu-Fernández V, Pascual Tutusaus M, Ferrero Martínez S, Gómez Roig MD, García-Algar O (2019) The Effects of Vegetarian and Vegan Diet during Pregnancy on the Health of Mothers and Offspring. Nutrients 11 (3):557. doi:10.3390/nu11030557
214. Singh PN, Sabate J, Fraser GE (2003) Does low meat consumption increase life expectancy in humans? Am J Clin Nutr 78 (3 SUPPL.):526S-532S
215. Skorek P, Glibowski P, Banach K (2019) Nutrition of vegetarians in Poland – a review of research. Rocz Panstw Zakl Hig 70 (3):217-223. doi:10.32394/rpzh.2019.0072
216. Van Winckel M, Vande Velde S, De Bruyne R, Van Biervliet S (2011) Clinical practice: vegetarian infant and child nutrition. Eur J Pediatr 170 (12):1489-1494. doi:10.1007/s00431-011-1547-x
217. Cullum-Dugan D, Pawlak R (2015) Position of the academy of nutrition and dietetics: vegetarian diets. J Acad Nutr Diet 115 (5):801-810. doi:10.1016/j.jand.2015.02.033
218. Adams M, Sabaté J (2019) Sexual Dimorphism in Cardiovascular Disease Risk and Risk Factors Among Vegetarians: an Exploration of the Potential Mechanisms. Current atherosclerosis reports 21 (9):35-35. doi:10.1007/s11883-019-0796-4
219. American Dietetic A, Dietitians of C (2003) Position of the American Dietetic Association and Dietitians of Canada: Vegetarian diets. J Am Diet Assoc 103 (6):748-765. doi:10.1053/jada.2003.50142
220. American Dietetic A, Dietitians of C (2003) Position of the American Dietetic Association and Dietitians of Canada: vegetarian diets. Canadian journal of dietetic practice and research : a publication of Dietitians of Canada = Revue canadienne de la pratique et de la recherche en dietetique : une publication des Dietetistes du Canada 64 (2):62-81. doi:10.3148/64.2.2003.62
221. Appleby PN, Thorogood M, Mann JI, Key TJ (1999) The Oxford Vegetarian Study: an overview. The American journal of clinical nutrition 70 (3 Suppl):525S-531S. doi:10.1093/ajcn/70.3.525s
222. Attaye I, Pinto-Sietsma S-J, Herrema H, Nieuwdorp M (2020) A Crucial Role for Diet in the Relationship Between Gut Microbiota and Cardiometabolic Disease. Annu Rev Med 71:149-161. doi:10.1146/annurev-med-062218-023720
223. Barnard ND, Goldman DM, Loomis JF, Kahleova H, Levin SM, Neabore S, Batts TC (2019) Plant-Based Diets for Cardiovascular Safety and Performance in Endurance Sports. Nutrients 11 (1):130. doi:10.3390/nu11010130
224. Battaglia Richi E, Baumer B, Conrad B, Darioli R, Schmid A, Keller U (2015) Health Risks Associated with Meat Consumption: A Review of Epidemiological Studies. International journal for vitamin and nutrition research Internationale Zeitschrift fur Vitamin- und Ernahrungsforschung Journal international de vitaminologie et de nutrition 85 (1-2):70-78. doi:10.1024/0300-9831/a000224
225. Burr M (1990) Vegetarianism and health. The Practitioner 234 (1481):62-64
226. Burr ML (1990) Vegetarianism, fish eating and jogging. J R Soc Health 110 (4):144-145. doi:10.1177/146642409011000413
227. Burr ML (1992) Particular diets and cardiovascular risk. Bibl Nutr Dieta 49:102-110. doi:10.1159/000421439

228. Campbell T (2017) A plant-based diet and stroke. J Geriatr Cardiol 14 (5):321-326. doi:10.11909/j.issn.1671-5411.2017.05.010
229. Caprara G (2018) Diet and longevity: The effects of traditional eating habits on human lifespan extension. Med J Nutrition Metab 11 (3):261-264. doi:http://dx.doi.org/10.3233/MNM-180225
230. Craig WJ (2009) Health effects of vegan diets. The American journal of clinical nutrition 89 (5):1627S-1633S. doi:10.3945/ajcn.2009.26736N
231. Craig WJ (2010) Nutrition concerns and health effects of vegetarian diets. Nutrition in clinical practice : official publication of the American Society for Parenteral and Enteral Nutrition 25 (6):613-620. doi:10.1177/0884533610385707
232. Craig WJ, Mangels AR, American Dietetic A (2009) Position of the American Dietetic Association: vegetarian diets. J Am Diet Assoc 109 (7):1266-1282. doi:10.1016/j.jada.2009.05.027
233. Dagnelie PC (2003) Nutrition and health--potential health benefits and risks of vegetarianism and limited consumption of meat in the Netherlands (Voeding en gezondheid--potentiële gezondheidsvoordelen en risico's van vegetarisme en beperkte vleesconsumptie in Nederland). Ned Tijdschr Geneeskd 147 (27):1308-1313
234. Deedwania P, Singh V (2005) Coronary artery disease in south Asians: Evolving strategies for treatment and prevention. Indian Heart J 57 (6):617-631
235. Desmond MA, Sobiecki J, Fewtrell M, Wells JCK (2018) Plant-based diets for children as a means of improving adult cardiometabolic health. Nutr Rev 76 (4):260-273. doi:10.1093/nutrit/nux079
236. Dinu M, Pagliai G, Sofi F (2017) A Heart-Healthy Diet: Recent Insights and Practical Recommendations. Curr Cardiol Rep 19 (10):95. doi:10.1007/s11886-017-0908-0
237. do Rosario VA, Fernandes R, Trindade EBSdM (2016) Vegetarian diets and gut microbiota: important shifts in markers of metabolism and cardiovascular disease. Nutr Rev 74 (7):444-454. doi:10.1093/nutrit/nuw012
238. Dominique Ashen M (2013) Vegetarian diets in cardiovascular prevention. Curr Treat Options Cardiovasc Med 15 (6):735-745. doi:10.1007/s11936-013-0266-1
239. Dwyer JT (1988) Health aspects of vegetarian diets. The American journal of clinical nutrition 48 (3 Suppl):712-738. doi:10.1093/ajcn/48.3.712
240. Enas EA, Mehta J (1995) Malignant coronary artery disease in young Asian Indians: thoughts on pathogenesis, prevention, and therapy. Coronary Artery Disease in Asian Indians (CADI) Study. Clin Cardiol 18 (3):131-135. doi:10.1002/clc.4960180305
241. Esselstyn CB (2017) A plant-based diet and coronary artery disease: a mandate for effective therapy. J Geriatr Cardiol 14 (5):317-320. doi:10.11909/j.issn.1671-5411.2017.05.004
242. Fischer NM, Pallazola VA, Xun H, Cainzos-Achirica M, Michos ED (2020) The evolution of the heart-healthy diet for vascular health: A walk through time. Vasc Med 25 (2):184-193. doi:10.1177/1358863x19901287
243. Fraser GE (1988) Determinants of ischemic heart disease in Seventh-day Adventists: a review. The American journal of clinical nutrition 48 (3 Suppl):833-836. doi:10.1093/ajcn/48.3.833
244. Fraser GE (1994) Diet and coronary heart disease: beyond dietary fats and low-density-lipoprotein cholesterol. The American journal of clinical nutrition 59 (5 Suppl):1117S-1123S. doi:10.1093/ajcn/59.5.1117S
245. Fraser GE (1999) Diet as primordial prevention in Seventh-Day Adventists. Prev Med 29 (6 Pt 2):S18-S23. doi:10.1006/pmed.1998.0415

246. Fraser GE (2009) Vegetarian diets: what do we know of their effects on common chronic diseases? The American journal of clinical nutrition 89 (5):1607S-1612S. doi:10.3945/ajcn.2009.26736K
247. Freeman AM, Morris PB, Barnard N, Esselstyn CB, Ros E, Agatston A, Devries S, O'Keefe J, Miller M, Ornish D, Williams K, Kris-Etherton P (2017) Trending Cardiovascular Nutrition Controversies. J Am Coll Cardiol 69 (9):1172-1187. doi:http://dx.doi.org/10.1016/j.jacc.2016.10.086
248. Gardner CD (2001) The role of plant-based diets in the treatment and prevention of coronary artery disease. Coron Artery Dis 12 (7):553-559. doi:10.1097/00019501-200111000-00005
249. Ginter E (2008) Vegetarian diets, chronic diseases and longevity. Bratisl Lek Listy 109 (10):463-466
250. Glueck CJ, Connor WE (1978) Diet-coronary heart disease relationships reconnoitered. The American journal of clinical nutrition 31 (5):727-737. doi:10.1093/ajcn/31.5.727
251. Gomez-Delgado F, Katsiki N, Lopez-Miranda J, Perez-Martinez P (2021) Dietary habits, lipoprotein metabolism and cardiovascular disease: From individual foods to dietary patterns. Crit Rev Food Sci Nutr 61 (10):1651-1669. doi:10.1080/10408398.2020.1764487
252. Hankey GJ (2012) Nutrition and the risk of stroke. The Lancet Neurology 11 (1):66-81. doi:http://dx.doi.org/10.1016/S1474-4422%2811%2970265-4
253. Hardinge MG, Crooks H (1963) Non-Flesh Dietaries. J Am Diet Assoc 43 (6):550-558
254. Havala S, Dwyer J (1993) Position of the American Dietetic Association: vegetarian diets. J Am Diet Assoc 93 (11):1317-1319. doi:10.1016/0002-8223(93)91966-t
255. Hemler EC, Hu FB (2019) Plant-Based Diets for Personal, Population, and Planetary Health. Advances in nutrition (Bethesda, Md) 10 (4 Supplementement):S275-S283. doi:http://dx.doi.org/10.1093/advances/nmy117
256. Hemler EC, Hu FB (2019) Plant-Based Diets for Cardiovascular Disease Prevention: All Plant Foods Are Not Created Equal. Current atherosclerosis reports 21 (18). doi:10.1007/s11883-019-0779-5
257. Hemler EC, Hu FB (2019) Plant-Based Diets for Personal, Population, and Planetary Health. Adv Nutr 10 (Suppl_4):S275-s283. doi:10.1093/advances/nmy117
258. Hever J, Cronise RJ (2017) Plant-based nutrition for healthcare professionals: implementing diet as a primary modality in the prevention and treatment of chronic disease. J Geriatr Cardiol 14 (5):355-368. doi:10.11909/j.issn.1671-5411.2017.05.012
259. Houston M (2018) The role of noninvasive cardiovascular testing, applied clinical nutrition and nutritional supplements in the prevention and treatment of coronary heart disease. Ther Adv Cardiovasc Dis 12 (3):85-108. doi:http://dx.doi.org/10.1177/1753944717743920
260. Houston M, Minich D, Sinatra ST, Kahn JK, Guarneri M (2018) Recent Science and Clinical Application of Nutrition to Coronary Heart Disease. J Am Coll Nutr 37 (3):169-187. doi:http://dx.doi.org/10.1080/07315724.2017.1381053
261. Joulie C (2021) Risks and benefits of vegetarianism and veganism (Risques et benefices du vegetarisme et du vegetalisme.). Actual Pharm 60 (602):23-27. doi:http://dx.doi.org/10.1016/j.actpha.2020.11.006
262. Kahleova H, Levin S, Barnard N (2017) Cardio-Metabolic Benefits of Plant-Based Diets. Nutrients 9 (8): 848. doi:10.3390/nu9080848
263. Kahleova H, Levin S, Barnard ND (2018) Vegetarian Dietary Patterns and Cardiovascular Disease. Prog Cardiovasc Dis 61 (1):54-61. doi:10.1016/j.pcad.2018.05.002

264. Kahleova H, Salas-Salvadó J, Rahelić D, Kendall CW, Rembert E, Sievenpiper JL (2019) Dietary Patterns and Cardiometabolic Outcomes in Diabetes: A Summary of Systematic Reviews and Meta-Analyses. Nutrients 11 (9):2209. doi:10.3390/nu11092209
265. Kaiser J, van Daalen KR, Thayyil A, Cocco M, Caputo D, Oliver-Williams C (2021) A Systematic Review of the Association Between Vegan Diets and Risk of Cardiovascular Disease. J Nutr 151 (6):1539-1552. doi:10.1093/jn/nxab037
266. Kendler BS (1997) Recent nutritional approaches to the prevention and therapy of cardiovascular disease. Prog Cardiovasc Nurs 12 (3):3-23
267. Key TJ, Appleby PN, Rosell MS (2006) Health effects of vegetarian and vegan diets. Proc Nutr Soc 65 (1):35-41. doi:http://dx.doi.org/10.1079/PNS2005481
268. Key TJ, Davey GK, Appleby PN (1999) Health benefits of a vegetarian diet. The Proceedings of the Nutrition Society 58 (2):271-275. doi:10.1017/s0029665199000373
269. Kim H, Rebholz CM (2021) Metabolomic Biomarkers of Healthy Dietary Patterns and Cardiovascular Outcomes. Curr Atheroscler Rep 23 (6):26. doi:10.1007/s11883-021-00921-8
270. Kimokoti RW, Millen BE (2016) Nutrition for the Prevention of Chronic Diseases. Med Clin North Am 100 (6):1185-1198. doi:http://dx.doi.org/10.1016/j.mcna.2016.06.003
271. Krajcovicova-Kudlackova M (2005) Health benefits of plant food. Klinicka Biochemie a Metabolismus 13 (4):168-171
272. Krajcovicová-Kudlácková M, Simoncic R, Béderová A (1997) Risks and advantages of the vegetarian diet (Riziká a prednosti vegetariánskej výživy). Cas Lek Cesk 136 (23):715-719
273. Krishnamoorthy KM (1999) Diet and coronary artery disease. Indian Heart J 51 (3):268-274
274. Lanou AJ, Svenson B (2010) Vegetarian Dietary Patterns as a Means to Achieve Reduction in Cardiovascular Disease and Diabetes Risk Factors. Curr Cardiovasc Risk Rep 4 (1):48-56. doi:http://dx.doi.org/10.1007/s12170-009-0075-y
275. Le LT, Sabaté J (2014) Beyond meatless, the health effects of vegan diets: findings from the Adventist cohorts. Nutrients 6 (6):2131-2147. doi:10.3390/nu6062131
276. Leitzmann C (2005) Vegetarian diets: what are the advantages? Forum Nutr 57:147-156. doi:10.1159/000083787
277. Li D (2014) Effect of the vegetarian diet on non-communicable diseases. J Sci Food Agric 94 (2):169-173. doi:10.1002/jsfa.6362
278. Liday C, Kirkpatrick C (2019) Optimal Dietary Strategies for Prevention of Atherosclerotic Cardiovascular Disease in Diabetes: Evidence and Recommendations. Curr Cardiol Rep 21 (11). doi:http://dx.doi.org/10.1007/s11886-019-1232-7
279. Lin CL (1997) Vegetarianism. Tzu Chi Medical Journal 9 (2):81-89
280. Lin CL (2021) Stroke and diets-A review. Tzu Chi Medical Journal 33 (3):238-242. doi:http://dx.doi.org/10.4103/tcmj.tcmj_168_20
281. McCarty MF (1999) Vegan proteins may reduce risk of cancer, obesity, and cardiovascular disease by promoting increased glucagon activity. Med Hypotheses 53 (6):459-485. doi:10.1054/mehy.1999.0784
282. McCarty MF (2003) IGF-I activity may be a key determinant of stroke risk--a cautionary lesson for vegans. Med Hypotheses 61 (3):323-334. doi:10.1016/s0306-9877(02)00241-4
283. McCarty MF (2004) A shift in myocardial substrate, improved endothelial function, and diminished sympathetic activity may contribute to the anti-anginal impact of very-low-fat diets. Med Hypotheses 62 (1):62-71. doi:10.1016/s0306-9877(03)00232-9
284. McEvoy CT, Temple N, Woodside JV (2012) Vegetarian diets, low-meat diets and health: a review. Public Health Nutr 15 (12):2287-2294. doi:10.1017/S1368980012000936

285. Medina-Remón A, Kirwan R, Lamuela-Raventós RM, Estruch R (2018) Dietary patterns and the risk of obesity, type 2 diabetes mellitus, cardiovascular diseases, asthma, and neurodegenerative diseases. Crit Rev Food Sci Nutr 58 (2):262-296. doi:10.1080/10408398.2016.1158690
286. Melina V, Craig W, Levin S (2016) Position of the Academy of Nutrition and Dietetics: Vegetarian Diets. J Acad Nutr Diet 116 (12):1970-1980. doi:10.1016/j.jand.2016.09.025
287. Migliaccio S, Brasacchio C, Pivari F, Salzano C, Barrea L, Muscogiuri G, Savastano S, Colao A (2020) What is the best diet for cardiovascular wellness? A comparison of different nutritional models. Int J Obes Suppl 10 (1):50-61. doi:10.1038/s41367-020-0018-0
288. Norman K, Klaus S (2020) Veganism, aging and longevity: new insight into old concepts. Curr Opin Clin Nutr Metab Care 23 (2):145-150. doi:10.1097/MCO.0000000000000625
289. O'Keefe JH, Torres-Acosta N, O'Keefe EL, Saeed IM, Lavie CJ, Smith SE, Ros E (2020) A Pesco-Mediterranean Diet With Intermittent Fasting: JACC Review Topic of the Week. J Am Coll Cardiol 76 (12):1484-1493. doi:10.1016/j.jacc.2020.07.049
290. Pallazola VA, Davis DM, Whelton SP, Cardoso R, Latina JM, Michos ED, Sarkar S, Blumenthal RS, Arnett DK, Stone NJ, Welty FK (2019) A Clinician's Guide to Healthy Eating for Cardiovascular Disease Prevention. Mayo Clin Proc Innov Qual Outcomes 3 (3):251-267. doi:10.1016/j.mayocpiqo.2019.05.001
291. Panagiotakos DB, Notara V, Kouvari M, Pitsavos C (2016) The Mediterranean and other Dietary Patterns in Secondary Cardiovascular Disease Prevention: A Review. Curr Vasc Pharmacol 14 (5):442-451. doi:10.2174/1570161114999160719104731
292. Penkov A (2005) Vegetarian diet in human health. Acta Medica Bulgarica 32 (1):22-34
293. Phillips F (2005) Vegetarian nutrition. Nutr Bull 30 (2):132-167. doi:http://dx.doi.org/10.1111/j.1467-3010.2005.00467.x
294. Pilis W, Stec K, Zych M, Pilis A (2014) Health benefits and risk associated with adopting a vegetarian diet. Rocz Panstw Zakl Hig 65 (1):9-14
295. Pratt C (2010) Alternative prevention and treatment of cardiovascular disease Part 1. Primary Care - Clinics in Office Practice 37 (2):325-337. doi:http://dx.doi.org/10.1016/j.pop.2010.02.009
296. Rajaram S, Sabaté J (2000) Health benefits of a vegetarian diet. Nutrition (Burbank, Los Angeles County, Calif) 16 (7-8):531-533. doi:10.1016/s0899-9007(00)00305-1
297. Ravera A, Carubelli V, Sciatti E, Bonadei I, Gorga E, Cani D, Vizzardi E, Metra M, Lombardi C (2016) Nutrition and cardiovascular disease: Finding the perfect recipe for cardiovascular health. Nutrients 8 (6):363. doi:http://dx.doi.org/10.3390/nu8060363
298. Richi EB, Baumer B, Conrad B, Darioli R, Schmid A, Keller U (2015) Health risks associated with meat consumption: A review of epidemiological studies. Int J Vitam Nutr Res 85 (1-2):70-78. doi:http://dx.doi.org/10.1024/0300-9831/a000224
299. Rocha JP, Laster J, Parag B, Shah NU (2019) Multiple Health Benefits and Minimal Risks Associated with Vegetarian Diets. Current nutrition reports 8 (4):374-381. doi:10.1007/s13668-019-00298-w
300. Sabaté J (1999) Nut consumption, vegetarian diets, ischemic heart disease risk, and all-cause mortality: evidence from epidemiologic studies. The American journal of clinical nutrition 70 (3 Suppl):500S-503S. doi:10.1093/ajcn/70.3.500s
301. Sabate J (2003) The contribution of vegetarian diets to health and disease: A paradigm shift? Am J Clin Nutr 78 (3 SUPPL.):502S-507S
302. Sabaté J (2003) The contribution of vegetarian diets to human health. Forum Nutr 56:218-220

303. Segasothy M, Phillips PA (1999) Vegetarian diet: panacea for modern lifestyle diseases? QJM : monthly journal of the Association of Physicians 92 (9):531-544. doi:10.1093/qjmed/92.9.531
304. Segovia-Siapco G, Sabaté J (2018) Health and sustainability outcomes of vegetarian dietary patterns: a revisit of the EPIC-Oxford and the Adventist Health Study-2 cohorts. Eur J Clin Nutr 72 (Suppl 1):60-70. doi:10.1038/s41430-018-0310-z
305. Singh PN, Arthur KN, Orlich MJ, James W, Purty A, Job JS, Rajaram S, Sabaté J (2014) Global epidemiology of obesity, vegetarian dietary patterns, and noncommunicable disease in Asian Indians. The American journal of clinical nutrition 100 Suppl 1 (1):359S-364S. doi:10.3945/ajcn.113.071571
306. Spence JD, Azarpazhooh MR, Larsson SC, Bogiatzi C, Hankey GJ (2020) Stroke Prevention in Older Adults: Recent Advances. Stroke 51 (12):3770-3777. doi:10.1161/strokeaha.120.031707
307. Sticher MA, Smith CB, Davidson S (2010) Reducing heart disease through the vegetarian diet using primary prevention. J Am Acad Nurse Pract 22 (3):134-139. doi:10.1111/j.1745-7599.2009.00483.x
308. Strohle A, Hahn A (2016) Health outcomes of vegetarian nutrition - An update. [German] (Vor- und Nachteile vegetarischer Ernahrungsformen - Aktueller wissenschaftlicher Kenntnisstand.). Ther Umsch 73 (11):659-672. doi:http://dx.doi.org/10.1024/0040-5930/a000848
309. Strohle A, Loser C, Behrendt I, Leitzmann C, Hahn A (2016) Alternative Diets: Part 1: Overview and Vegetarian Diets. [German] (Alternative Ernahrungsformen: Teil 1: Allgemeine Aspekte und vegetarische Kostformen.). Aktuelle Ernahrungsmedizin 41 (1):47-65. doi:http://dx.doi.org/10.1055/s-0041-111459
310. Ströhle A, Löser C, Behrendt I, Leitzmann C, Hahn A (2018) Alternative Ernährungsformen: Allgemeine Aspekte und vegetarische Kostformen. (Alternative diets: general aspects and vegetarian diets) [German]. Die Rehabilitation 57 (1):55-70. doi:10.1055/s-0043-124048
311. Thorogood M (1994) Vegetarianism, coronary disease risk factors and coronary heart disease. Curr Opin Lipidol 5 (1):17-21. doi:10.1097/00041433-199402000-00004
312. Tindall AM, Petersen KS, Kris-Etherton PM (2018) Dietary Patterns Affect the Gut Microbiome-The Link to Risk of Cardiometabolic Diseases. The Journal of nutrition 148 (9):1402-1407. doi:10.1093/jn/nxy141
313. Trautwein EA, McKay S (2020) The Role of Specific Components of a Plant-Based Diet in Management of Dyslipidemia and the Impact on Cardiovascular Risk. Nutrients 12 (9):2671. doi:10.3390/nu12092671
314. Willcox DC, Scapagnini G, Willcox BJ (2014) Healthy aging diets other than the Mediterranean: A focus on the Okinawan diet. Mech Ageing Dev 136:148-162. doi:http://dx.doi.org/10.1016/j.mad.2014.01.002
315. Wong JMW (2014) Gut microbiota and cardiometabolic outcomes: influence of dietary patterns and their associated components. The American journal of clinical nutrition 100 Suppl 1:369S-377S. doi:10.3945/ajcn.113.071639
316. Wong M-W, Yi C-H, Liu T-T, Lei W-Y, Hung J-S, Lin C-L, Lin S-Z, Chen C-L (2018) Impact of vegan diets on gut microbiota: An update on the clinical implications. Ci Ji Yi Xue Za Zhi 30 (4):200-203. doi:10.4103/tcmj.tcmj_21_18
317. Woo KS, Kwok TCY, Celermajer DS (2014) Vegan diet, subnormal vitamin B-12 status and cardiovascular health. Nutrients 6 (8):3259-3273. doi:10.3390/nu6083259
318. Zampelas A, Magriplis E (2020) Dietary patterns and risk of cardiovascular diseases: a review of the evidence. The Proceedings of the Nutrition Society 79 (1):68-75. doi:10.1017/S0029665119000946

319. Zarraga IGE, Schwarz ER (2006) Impact of dietary patterns and interventions on cardiovascular health. Circulation 114 (9):961-973. doi:http://dx.doi.org/10.1161/CIRCULATIONAHA.105.603910
320. Archundia Herrera MC, Subhan FB, Chan CB (2017) Dietary Patterns and Cardiovascular Disease Risk in People with Type 2 Diabetes. Current obesity reports 6 (4):405-413. doi:10.1007/s13679-017-0284-5
321. Arntzenius AC (1986) Diet, lipoproteins and the progression of coronary atherosclerosis. The Leiden Intervention Trial. Drugs 31 Suppl 1:61-65. doi:10.2165/00003495-198600311-00011
322. Arntzenius AC, Kromhout D, Barth JD, Reiber JH, Bruschke AV, Buis B, van Gent CM, Kempen-Voogd N, Strikwerda S, van der Velde EA (1985) Diet, lipoproteins, and the progression of coronary atherosclerosis. The Leiden Intervention Trial. The New England journal of medicine 312 (13):805-811. doi:10.1056/NEJM198503283121301
323. de Lorgeril M, Salen P, Monjaud I, Delaye J (1997) The 'diet heart' hypothesis in secondary prevention of coronary heart disease. Eur Heart J 18 (1):13-18. doi:10.1093/oxfordjournals.eurheartj.a015094
324. Djekic D, Carlsson F, Landberg R, Sarnqvist C, Cao Y, Tremaroli V, Backhed F, Frobert O (2019) VEgetaRian Diet in patients with Ischemic heart disease (VERDI): An open-label, randomized, prospective, cross-over study. Eur Heart J 40 (Supplement 1):3819. doi:http://dx.doi.org/10.1093/eurheartj/ehz746.0813
325. Djekic D, Shi L, Brolin H, Carlsson F, Särnqvist C, Savolainen O, Cao Y, Bäckhed F, Tremaroli V, Landberg R, Frøbert O (2020) Effects of a Vegetarian Diet on Cardiometabolic Risk Factors, Gut Microbiota, and Plasma Metabolome in Subjects With Ischemic Heart Disease: A Randomized, Crossover Study. J Am Heart Assoc 9 (18):e016518. doi:10.1161/jaha.120.016518
326. Djekic D, Shi L, Calais F, Carlsson F, Landberg R, Hyötyläinen T, Frøbert O (2020) Effects of a Lacto-Ovo-Vegetarian Diet on the Plasma Lipidome and Its Association with Atherosclerotic Burden in Patients with Coronary Artery Disease-A Randomized, Open-Label, Cross-over Study. Nutrients 12 (11): 3586. doi:10.3390/nu12113586
327. Dogra S, Getz A, Woolf K, Newman JD, Xia Y, Slater J, Shah B (2020) Long-term Dietary and Weight Changes Following a Short-term Dietary Intervention Study: 4-year Follow-up of the Evade Cad Trial. Circulation Conference: American Heart Association Scientific Sessions, AHA 142 (Suppl 3):A15565. doi:http://dx.doi.org/10.1161/circ.142.suppl_3.15565
328. Esselstyn CB, Jr. (1999) Updating a 12-year experience with arrest and reversal therapy for coronary heart disease (an overdue requiem for palliative cardiology). The American journal of cardiology 84 (3):339-A338. doi:10.1016/s0002-9149(99)00290-8
329. Esselstyn CB, Jr., Gendy G, Doyle J, Golubic M, Roizen MF (2014) A way to reverse CAD? The Journal of family practice 63 (7):356-364b
330. Gould KL, Ornish D, Scherwitz L, Brown S, Edens RP, Hess MJ, Mullani N, Bolomey L, Dobbs F, Armstrong WT, et al. (1995) Changes in myocardial perfusion abnormalities by positron emission tomography after long-term, intense risk factor modification. JAMA 274 (11):894-901. doi:10.1001/jama.1995.03530110056036
331. Gupta P, Arora S, Bansal A, Qamar A, Kurien S, Palleda G, Batra V, Mahajan S, Hendrickson M, Jain G, Safal S, Khosla AA, Vaduganathan M, Bhatt D, Gupta M (2021) Risk Factors and Outcomes of Young Adults Who Experience Myocardial Infarction: Insights from the Norin-Stemi Registry. J Am Coll Cardiol 77 (18 Supplement 1):110. doi:http://dx.doi.org/10.1016/S0735-1097%2821%2901469-8
332. Gupta SK, Sawhney RC, Rai L, Chavan VD, Dani S, Arora RC, Selvamurthy W, Chopra HK, Nanda NC (2011) Regression of coronary atherosclerosis through healthy

lifestyle in coronary artery disease patients--Mount Abu Open Heart Trial. Indian Heart J 63 (5):461-469
333. Ivanov AN, Medkova IL, Mosiagina LI (2002) Vegetarian diet in treating elderly patients with ischemic heart disease (clinico-hemodynamic, biochemical, and hemorheological effects) (Vegetarianskaia dieta v lechenii bol'nykh ishemicheskoĭ bolezn'iu serdtsa pozhilogo vozrasta (kliniko-gemodinamicheskie biokhimicheskie i gemoreologicheskie éffekty)). Vopr Pitan 71 (3):11-14
334. Jhatakia KU (1973) Profile of coronary artery disease in vegetarian community. Indian Heart J 25 (2):94-99
335. Kolenda KD, Muller MJ (2005) How effective is dietary therapy for secondary prevention in patients with coronary heart disease?. [German] (Wie wirksam ist eine ernahrungstherapie bei patienten mit koronarer herzkrankheit in der sekundarpravention?.). Tagliche Praxis 46 (2):381-392
336. listed Na (1997) Ornish lifestyle modification program continues to produce impressive outcomes for CHD. Healthc Demand Dis Manag 3 (4):59-61
337. Marshall DA, Walizer EM, Vernalis MN (2009) Achievement of heart health characteristics through participation in an intensive lifestyle change program (Coronary Artery Disease Reversal Study). J Cardiopulm Rehabil Prev 29 (2):84-96. doi:10.1097/HCR.0b013e31819a00b2
338. Medkova IL, Mosiakina LI, Pavlova VE, Zharkov AP, Bugaev VA, Koryshev VI (1997) Balanced vegetarian diet in combined rehabilitation of patients suffering from ischemic heart disease (Opyt ispol'zovaniia sbalansirovannoi vegetarianskoi diety v kompleksnoi reabilitatsii bol'nykh izhemicheskoi bolezn'iu serdtsa). Klin Med (Mosk) 75 (1):28-31
339. Medkova IL, Pustynnikova VE, Mosiakina LI, Ieromuzo AA (1997) The results of exposure to an antisclerotic vegetarian diet enriched with soy-based products on patients in the secondary prevention of ischemic heart disease (Rezul'taty vozdeĭstviia protivosklerotičheskoĭ vegetarianskoĭ diety, obogashchennoĭ produktami pererabotki soi, na bol'nykh pri vtorichnoĭ profilaktike ishemicheskoĭ bolezni serdtsa). Ter Arkh 69 (9):52-55
340. Misquitta R, Ergas I, Edwards L, Rahbar J, Kushi L (2020) Preliminary evaluation of the health achieved through lifestyle transformation (HALT) program, a kaiser permanente lifestyle intervention. J Altern Complement Med 26 (11):A9. doi:http://dx.doi.org/10.1089/acm.2020.29085.abstracts
341. Neelamegam V, Veeramani SR, Balasubramanian S, Selvarani G, Ramesh R, Saravanan M, Nagasundar G, Saravanan RR, Sathish Kumar S, Hemanath TR, Sivakumar GS (2019) Clinical and Angiographic Profile of Premenopausal women with STEMI. Indian Heart J 71 (Supplement 1):S81. doi:http://dx.doi.org/10.1016/j.ihj.2019.11.185
342. Ornish D, Brown SE, Scherwitz LW, Billings JH, Armstrong WT, Ports TA, McLanahan SM, Kirkeeide RL, Brand RJ, Gould KL (1990) Can lifestyle changes reverse coronary heart disease? The Lifestyle Heart Trial. Lancet (London, England) 336 (8708):129-133. doi:10.1016/0140-6736(90)91656-u
343. Ornish D, Scherwitz LW, Billings JH, Brown SE, Gould KL, Merritt TA, Sparler S, Armstrong WT, Ports TA, Kirkeeide RL, Hogeboom C, Brand RJ (1998) Intensive lifestyle changes for reversal of coronary heart disease. JAMA 280 (23):2001-2007. doi:10.1001/jama.280.23.2001
344. Shah B, Ganguzza L, Slater J, Newman JD, Allen N, Fisher E, Larigakis J, Ujueta F, Gianos E, Guo Y, Woolf K (2017) The Effect of a Vegan versus AHA DiEt in Coronary Artery Disease (EVADE CAD) trial: study design and rationale. Contemporary clinical trials communications 8:90-98. doi:10.1016/j.conctc.2017.09.003

345. Rees K, Al-Khudairy L, Takeda A, Stranges S (2019) Vegan dietary pattern for the primary and secondary prevention of cardiovascular diseases. Cochrane Database Syst Rev (12):CD013501. doi:http://dx.doi.org/10.1002/14651858.CD013501
