# Supplementary material for: Vegetarian and vegan diets and the risk of cardiovascular disease, ischemic heart disease and stroke: a systematic review and meta-analysis of prospective cohort studies
Source: Eur J Nutr. 2022 Aug 27;62(1):51–69. doi: 10.1007/s00394-022-02942-8 (PMC9899747; doi:10.1007/s00394-022-02942-8)
Supplement: Supplementary file 4 — Supplementary file4 (PDF 1269 KB) [file 394_2022_2942_MOESM4_ESM.pdf]

Online Resource 4

Supplementary material to: Vegetarian and vegan diets and the risk of cardiovascular disease, ischemic heart disease and stroke: a systematic review and meta-analysis of prospective cohort studies.

Authors: Jarle Sæby Dybvik[1], Mette Svendsen[2,3] Dagfinn Aune[3,4,5,6]

Author affiliations

[1] Institute of Clinical Medicine, Faculty of Medicine, University of Oslo, Oslo, Norway

[2] Department of Nutrition Sciences, Institute of Basic Medical Sciences, University of Oslo, Oslo, Norway

[3] Department of Endocrinology, Morbid Obesity and Preventive Medicine, Oslo University Hospital, Oslo, Norway

[4] Department of Epidemiology and Biostatistics, School of Public Health, Imperial College London, London, United Kingdom

[5] Department of Nutrition, Oslo New University College, Oslo, Norway

[6] Unit of Cardiovascular and Nutritional Epidemiology, Institute of Environmental Medicine, Karolinska Institutet, Stockholm, Sweden

Correspondence to: Jarle Sæby Dybvik, Institute of Clinical Medicine, Faculty of Medicine, University of Oslo, Oslo, Norway. E-mail address: j.s.dybvik@gmail.com. Phone number: 0047 99162306

Supplementary Table 5. Signalling questions and judgement criteria for risk of bias assessment on each domain overall with ROBINS-I [1]

**Domain**	**Signalling questions**	**Criteria for domain-level judgement**
1. Bias due to confounding	Is there potential for baseline confounding of the effect of exposure?<br><br>Did the authors use a multivariable-adjusted analysis that controlled for all important confounding domains?	Low risk of bias[2]:<br>No bias is expected due to confounding.<br><br>Moderate risk of bias[3]:<br>Residual confounding is expected in observational studies:<br>All known important confounding domains (sociodemographic [age, sex] and lifestyle [alcohol, smoking, physical activity] domain) have been appropriately measured and controlled for with a multivariate adjusted model (e.g., Poisson regression, cox hazards regression model)<br><br>Serious risk of bias[4]:<br>At least one important domain (sociodemographic [age, sex] and lifestyle [alcohol, smoking, physical activity] domain) was not appropriately measured and controlled for.
2. Bias in selection of participants into the study	Was selection of participants into the study (or into the analysis) based on variables measured after start of the exposure?<br><br>Were the post-exposure variables that influenced selection associated with exposure *and* outcome (or a cause of the outcome)?<br><br>Do start of follow-up and start of exposure coincide for most participants?	Low risk of bias[2]:<br>(i) All participants who would have been eligible for the target trial were included in the study ***and*** (ii) The authors conducted a sensitivity analysis excluding CVD, IHD, or stroke cases which occurred <2 years after start of the study and the results did not change.<br><br>Moderate risk of bias[3]:<br>(i) Selection into the study (or analysis) *may* have been related to exposure and outcome (e.g., no exclusion of participants with a history of CVD) and the authors used appropriate methods to correct for selection bias and results changed<br>***or*** (ii) Selection into the study (or analysis) *may* have been related to exposure and outcome (e.g., no exclusion of participants with a history of CVD) and the authors conducted no sensitivity analysis excluding CVD, IHD, or stroke cases which occurred <2 years after the start.<br><br>Serious risk of bias[4]:<br>Selection into the study *was* related to exposure and outcome (e.g., only participants with

	Were adjustment techniques used that are likely to correct for the presence of selection biases?	chest pain/angina or CVD risk factors were included in the analysis); and this could not be corrected for in the analyses; or the start of follow-up and start of exposure do not coincide.
3. Bias in classification the exposure	Were diet groups (exposure and comparator) well defined and assessment methods robust?<br><br>Could classification of diet groups have been affected by knowledge of the outcome or risk of the outcome?	Low risk of bias[2]:<br>(i) The diet groups (exposure and comparator) are well defined<br>***and*** (ii) we do not expect misclassification due to the assessment method<br><br>Moderate risk of bias[3]:<br>(i) The diet groups (exposure and comparator) are well defined<br>***and*** (ii) dietary habits were assessed using a validated tool (FFQ, 24-hour recall) or by structured interview.<br><br>Serious risk of bias[4]:<br>(i) The diet groups (exposure and comparator) are not well defined<br>***or*** (ii) dietary habits was assessed with unvalidated tools.
4. Bias due to departures from intended exposure	Is there concern that changes in diet groups might have occurred among participants (e.g., changed diet group)?<br><br>Were there changes in important covariates that were unbalanced across diet groups?<br><br>Were adjustment techniques used that are likely to correct for these issues?	Low risk of bias[2]:<br>Repeated measurements of diet groups and covariates during follow-up are available and there were no deviations in terms of diet adherence (complete or partial changes in dietary patterns) and the authors conducted analysis based on these repeated measurements.<br><br>Moderate risk of bias[3]:<br>(i) Repeated measurements of diets groups and covariates during follow-up are available and there were deviations in terms of diet adherence (complete or partial changes in dietary patterns), but their impact on the outcome is expected to be slight.<br><br>**or** (iii) repeated measurements but any dietary changes are only expected to impact the outcome slightly

5. Bias due to missing data	Were participants excluded from analysis due to…:<br>1.Missing outcome data?<br>2.Missing exposure data?<br>3.Missing covariate data?<br><br>Are the proportion and reasons for missing data similar across diet groups?<br><br>Were appropriate statistical methods used to account for missing data?	Low risk of bias[2]:<br>(i) Data was reasonably complete (e.g., <10% loss to follow-up and <11% missing dietary and covariate data)<br>***or*** (ii) we do not suspect 'differential missingness' (def. proportions and reasons for missing data differing across groups)<br>***or*** (iii) the analysis addressed missing data and is likely to have removed any risk of bias.<br><br>Moderate risk of bias[3]:<br>(i) The data was less than reasonably complete (e.g., > 10% loss to follow-up and >11% missing dietary and covariate data)<br>**and** there is a possibility of 'differential missingness' (def. proportions and reasons for missing data differing across groups)<br>***and*** (ii) The analysis is unlikely to have removed the risk of bias arising from the missing data.
6. Bias in measurement of outcomes	Could the outcome measure (or assessor) have been influenced by knowledge of diet groups?<br><br>Were the methods of outcome assessment comparable across diet groups?<br><br>Were any systematic errors in outcome measurement related to exposure status?	Low risk of bias[2]:<br>(i) The methods of outcome assessment were comparable across diet groups<br>***and*** (ii) the outcome measure (or assessor) was unlikely to be influenced by knowledge of the participants diet group (i.e., is objective or likely unaware)<br>***and*** (iii) any error in measuring the outcome is unrelated to diet groups.<br><br>Moderate risk of bias[3]:<br>(i) The methods of outcome assessment were comparable across diet groups<br>***and*** (ii) the outcome measure (or assessor) is only minimally influenced by knowledge of the participants diet groups<br>***and*** (iii) Any error in measuring the outcome is only minimally related to diet groups.
7. Bias in selection of the	Is the reported effect estimate likely to be	Low risk of bias[2]:<br>There is clear evidence (usually through examination of a pre-registered protocol or

reported results	selected, on the basis of the results, from...:<br>1) multiple outcome measurements within the outcome domain?<br><br>2) multiple analyses of the exposure-outcome relationship?<br><br>3) different subgroups?	statistical analysis plan) that all reported results correspond to all intended outcomes, analyses, and sub-cohorts.<br><br>Moderate risk of bias[3]:<br>(i) The outcome measurements and analyses are consistent with an a priori plan or are clearly defined and both internally and externally consistent<br>***and*** (ii) There is no indication of selection of the reported analysis from among multiple analyses<br>***and*** (iii) There is no indication of selection of the cohort or subgroups for analysis and reporting based on the results.

From: Sterne JAC, Higgins JPT, Elbers RG, Reeves BC, and the development group for ROBINS-I Risk Of Bias In Non-randomized Studies of Interventions (ROBINS-I): detailed guidance. Available from: http://www.riskofbias.info [Updated Oct 12, 2016. Accessed Feb 26, 2022].

[1]The criteria for judging the risk of bias for each domain overall were modified in several domains to better reflect the basis for each judgement assigned when assessing risk of bias with ROBINS-I.

[2] Low risk of bias: the study is comparable to a well-performed randomized trial in this domain

[3] Moderate risk of bias: the study is sound for a non-randomized study in this domain but is not comparable to a well-performed randomized trial

[4] Serious risk of bias: the study has some important problems in this domain

Supplementary Table 6. Criteria for risk of bias assessment for each study overall using ROBINS-I [1]

**Overall study judgement**	**Criteria**
Low risk of bias:<br>The study is comparable to a well-performed randomized trial	The study is judged to be at low risk of bias for all domains
Moderate risk of bias:<br>The study appears to provide sound evidence for a non-randomized study but cannot be considered comparable to a well-performed randomized trial	The study is judged to be at low or moderate risk of bias for all domains
Serious risk of bias:<br>The study has some important problems	The study is judged to be at serious risk of bias in at least one domain, but not at critical risk of bias in any domain
Critical risk of bias:<br>The study is too problematic in this domain to provide any useful evidence on the effects of exposure	The study is judged to be at critical risk of bias in at least one domain
No information:<br>No information on which to base a judgement about risk of bias	There is no clear indication the study is at serious or critical risk of bias *and* there is a lack of information in one or more key domains of bias (*a judgement is required for this*)

From: Sterne JAC, Higgins JPT, Elbers RG, Reeves BC, and the development group for ROBINS-I Risk Of Bias In Non-randomized Studies of Interventions (ROBINS-I): detailed guidance. Available from: http://www.riskofbias.info [Updated Oct 12, 2016. Accessed Feb 26, 2022].

[1]The criteria for judging the risk of bias for each domain overall were modified in some domains to better reflect the basis for each judgement assigned when assessing risk of bias with ROBINS-I.

Supplementary Table 7. World Cancer Research Fund (WCRF) grading criteria

Grading	Criteria
Convincing	A convincing relationship should be robust enough to be highly unlikely to be modified in the foreseeable future as new evidence accumulates. All of the following are generally required:<br>- Evidence from more than one study type<br>- Evidence from at least two independent cohort studies<br>- No substantial unexplained heterogeneity within or between study types or in different populations relating to the presence or absence of an association, or direction of effect<br>- Good quality studies to exclude with confidence the possibility that the observed association results from random or systematic error, including confounding, measurement error, and selection bias<br>- Presence of a plausible biological gradient in the association. Such a gradient need not be linear or even in the same direction across different levels of exposure, so long as this can be explained plausibly<br>- Strong and plausible experimental evidence, either from human studies or relevant animal models, that typical human exposures can lead to relevant outcomes
Probable	All of the following are generally required:<br>- Evidence from at least two independent cohort studies, or at least five case-control studies

	- No substantial unexplained heterogeneity within or between study types or in different populations relating to the presence or absence of an association, or direction of effect<br>- Good quality studies to exclude with confidence the possibility that the observed association results from random or systematic error, including confounding, measurement error, and selection bias<br>- Evidence for biological plausibility
Limited - suggestive	All of the following are generally required:<br>- Evidence from at least two independent cohort studies, or at least five case-control studies<br>- The direction of effect is generally consistent though some unexplained heterogeneity may be present<br>- Evidence for biological plausibility
Limited - no conclusion	Evidence is so limited that no firm conclusion can be made, but this does not mean that there is evidence of no relationship. The evidence might be graded "limited - no conclusion" for several reasons:<br>- limited number of studies<br>- inconsistency of direction of effect<br>- poor quality of studies (e.g., lack of adjustment for known confounders)<br>- or any combination of these factors
Substantial effect on risk unlikely	All of the following are generally required:<br>- Evidence from more than one study type<br>- Evidence from at least two independent cohort studies

	- Summary estimate of effect close to 1.0 for comparison of high versus low exposure categories<br>- No substantial unexplained heterogeneity within or between study types or in different populations<br>- Good quality studies to exclude with confidence the possibility that the absence of association results from random or systematic error, including inadequate power, imprecision or error in exposure measurement, inadequate range of exposure, confounding, and selection bias<br>- Absence of a demonstrable biological gradient (dose response)<br>- Absence of strong and plausible experimental evidence, either from human studies or relevant animal models, that typical human exposures lead to relevant outcomes

From: World Cancer Research Fund/American Institute for Cancer Research (2018) Continuous Update Project Expert Report 2018. Judging the evidence. Available at: dietandcancerreport.org.

Specific upgrading factors:

1) Presence of a plausible biological gradient (dose response) in the association. Such a gradient need not be linear or even in the same direction across the different levels of exposure, so long as this can be explained plausibly.

2) A particularly large summary effect size (an odds ratio or relative risk of 2.0 or more, depending on the unit of exposure) after appropriate control for confounders.

3) Evidence from randomised trials in humans.

4) Evidence from appropriately controlled experiments demonstrating one or more plausible and specific mechanisms actually operating in humans.

5) Robust and reproducible evidence from experimental studies in appropriate animal models showing that typical human exposures can lead to relevant health outcomes.

Supplementary Table 8. General considerations applied for ROBINS risk of bias judgements of studies included in main analysis [1]

**Bias due to confounding**: Although the ROBINS-I states that there should be information regarding the validity and reliability of the measurement of the confounders that were adjusted for, this is almost never reported on in most research articles. We therefore considered it sufficient that there was adjustment for confounding variables for this domain to be fulfilled, without any mention of the validity and reliability on the measurement of the confounders.<br><br>We considered bias due to confounding if an unmeasured confounder was associated with both the exposure and the outcome. As residual confounding is expected in observational studies due to the inability to control for unknown confounders, no studies could be judged 'low risk of bias' in this domain. 'Moderate risk of bias' was assigned to nine studies (AHS-2, EPIC-Oxford, HBS, HPFS, NHS1, NHS2, TCHS, TCVS, UKB [six publications]) [1-6] which adjusted for all important confounding domains (sociodemographic [age, sex] and lifestyle [alcohol, smoking, physical activity] factors). 'Serious risk of bias' was assigned to four studies (AMS, AHS-1, HFSS, OVS [two publications]) [7,8] because one or more important confounding domains (sociodemographic [age, sex] and lifestyle [alcohol, smoking, physical activity] factors) was not appropriately controlled for.
**2. Bias in selection of participants into the study:** This domain was concerned with the selection of participants into the study (or analysis) based on participant characteristics observed after start of the exposure (before study entry) and bias was considered possible only if the selection could be influenced by both the exposure and outcome or a cause of the outcome. Note that this domain is not concerned with issues of generalisability (or external validity). In all the studies, the participants were selected based on their dietary preference (vegetarian, vegan or nonvegetarian), but we assessed whether the studies had excluded participants with a prior history of cardiovascular disease (CVD) and whether the authors had performed an appropriate sensitivity analysis after excluding the initial follow-up time (to control for reverse causality).<br><br>In two studies (EPIC-Oxford, UKB [publications]) [5,6], all participants that were eligible for the target trial were included in the analysis (by excluding cases with a history of CVD from analysis) and the authors conducted a sensitivity analysis that excluded the initial follow-up to assess for reverse causality. Eleven studies (AMS, AHS-1, AHS-2, HBS, HFSS, OVS, HPFS, NHS1, NHS2, TCHS, TCVS [six publications]) [1-4,7,9] were judged 'moderate' risk of bias as the selection of participants might have been related to the exposure and outcome either due to lack of 1) exclusion of subjects with prevalent CVD or 2) the lack of a sensitivity analysis that excluded the initial follow-up time (and could therefore not assess for reverse causality). No studies were judged serious risk of bias in this domain as no studies were conducted exclusively in patient groups (e.g, including only subjects with chest pain/angina).

**3. Bias in classification exposures:** We evaluated the methods used for assessment of dietary habits in each study as this is a crucial step in avoiding misclassification of subjects to diet groups. The lowest bias judgement given in this domain was 'moderate risk of bias' and this was assigned to nine studies (AHS-1, AHS-2, EPIC-Oxford, HPFS, NHS1, NHS2, TCHS, TCVS, UKB [six publications]) [1,3-6,8] that reported the use of a validated food frequency questionnaire (FFQ). As this method relies on the participants ability to estimate their food habits the previous year, there is always a potential for misclassifying subjects with this method. Therefore, an assignment of 'low risk of bias' was not deemed possible for any study in this domain. It was also important that study authors had a clear definition of the exposure (vegetarian or vegan diets) and the comparator (nonvegetarian diets) groups. Four studies (AMS, HBS, HFSS, OVS) [three publications]) [2,7,8] were judged to have 'serious risk of bias' as they did not report information about a validation process, although they did apply an FFQ.
**4. Bias due to departures from intended exposures:**<br>We did not make the distinction between co-exposures and confounders as this is less relevant in observational studies (co-exposures are usually considered confounders) [10]. Changes in important confounders (e.g., smoking, alcohol consumption and physical activity) and diet adherence could be assessed directly if studies measured variables with repeated FFQs/questionnaires. Deviations from the initial (or baseline) diet group (vegetarian, vegan or nonvegetarian) were defined as either complete changes (from one diet group to another) or partial changes (eating less or more healthy diets without changing diet groups) in dietary habits. If studies did not apply a repeated FFQ, we considered whether the proportions of participants in each diet group gave reason to suspect that potential dietary changes could impact on the risk estimates and if so the direction and magnitude of this effect.<br><br>Four studies (EPIC-Oxford, HPFS, NHS1, NHS2 [two publications]) [1,6], conducted analyses based on repeated FFQs, therefore we judged these studies to be at 'low risk of bias'.<br><br>For nine studies (AMS, AHS-1, AHS-2, HBS, HFSS, OVS, TCHS, TCVS, UKB [six publications]) [2-5,7,8], there were no reports of a repeated FFQ, however, dietary changes that could potentially affect the risk estimates were considered to most likely only bias results towards the null so that all these studies were judged to have 'moderate risk of bias'.
**5. Bias due to missing data:** Exclusion of participants with missing data was common to all studies, and bias was considered plausible to a varying degree if the proportions and reasons for missing data were considered un-evenly spread across the diet groups, referred to as 'differential missingness'. Missing data could be due to loss to follow-up or due to missing dietary and covariate data. We considered all studies to be at 'low risk of bias' with regard to loss to follow-up, and for nine of the studies this was reported in the included publications (AHS-2, EPIC-Oxford, HBS, HPFS, NHS1, NHS2, TCHS, TCVS, UKB [six publications]) [1-6] as outcome data was reasonably complete (e.g., loss to follow-up < 10 %) and 'differential missingness' was not suspected. For four studies (AHS-1, AMS, HFSS, OVS [two publications]) [7,8] there was no information on missing data in the included publications, but other publications (Fraser 1999, Snowdon 1984,

Key 1996) [9,11,12] from three of the same studies (AHS-1, AMS, HFSS) reported high completeness of follow-up and we considered this was also likely to apply to the OVS [7] considering the similar setting to the other UK cohorts (EPIC-Oxford, HFSS, UKB) [5-7] included. Five studies (HBS, AHS-2, EPIC-Oxford, TCVS, UKB [five publications]) [2-6] reported the number of participants excluded due to missing data on diet and confounders to be relatively low (<11 %). No information was available on this in the remaining studies (AHS-1, AMS, HFSS, OVS, HPFS, NHS1, NHS2, TCHS [four publications]) [1,3,7,8], but based on those studies that provided this information we considered it likely that this was low and therefore judged studies to have a low risk of bias on this point for the remaining studies as well.
**6. Bias in measurement of outcomes:** We assessed the risk of bias related to the method of outcome ascertainment reported in each study. The possibility of bias due to ‘differential measurement errors’ was considered small as 10 studies (AHS-1, AHS-2, AMS, EPIC-Oxford, HBS, HFSS, OVS, TCHS, TCVS, UKB [six publications]) [3-8] relied on objective measurement methods (e.g., record linkage) that could not have been influenced by knowledge of the diet-group (exposure, comparator), and these studies were therefore judged ‘low risk of bias’. However, the possibility of bias due to non-differential measurement errors (no knowledge of diet-group) could never be fully excluded, and three studies (HPFS, NHS1, NHS2) [one publication] [1] were judged ‘moderate risk of bias’, as some of the outcome data relied on the participants own reporting of incident stroke events.
**7. Bias in selection of the reported results:** This domain was concerned with the selective reporting of fully reported results (and not bias due to non-reporting). A pre-requisite for judging a study ‘low risk of bias’ was the reporting of a prospectively registered analysis plan (study protocol) to allow for assessment of coherency with the published paper, however, this is usually not reported in observational studies and was not reported in any of the studies included. For this reason, we assessed whether the outcomes reported on in the results section were consistent with those described in the statistical methods section. Risk of bias was evaluated on three levels: (i) selective reporting of an outcome measurement from multiple measurements of an outcome domain, (ii) selective reporting of a specific analysis when multiple analyses was performed (e.g., analysis adjusting for confounders vs. unadjusted analysis) and last (iii) selective reporting of results for a specific subset of the study sample. Bias was not recognized on any of these levels. No studies were judged ‘low risk of bias’ as they did not report information about a pre-registered study protocol with a clear analysis plan, but there were no incoherencies between the analyses described in the methods section and the results reported so all thirteen studies (AHS-1, AHS-2, AMS, EPIC-Oxford, HBS, HFSS, HPFS, NHS1, NHS2, OVS, TCHS, TCVS, UKB [eight publications]) [1-8] were judged to have ‘moderate risk of bias’.

Abbreviations: AHS-1 Adventist Health Study 1; AHS-2 Adventist Health Study 2; AMS Adventist Mortality Study; CVD cardiovascular disease; EPIC-Oxford European Prospective Investigation into Cancer and Nutrition - Oxford; HBS Heidelberg Study; HFSS Health Food

Shoppers Study; HPFS Health Professionals Follow-up Study; NHS1 Nurses' Health Study 1; NHS2 Nurses' Health Study 2; OVS Oxford Vegetarian Study; TCHS Tzu Chi Health Study; TCVS Tzu Chi Vegetarian Study; UKB UK Biobank Study

[1] We used risk estimates from two different publications for the Heidelberg Study in our main analysis depending on the outcome analysed, however, only the paper by Chang-Claude et al 2005 [2] is part of the count for the number of studies referenced in the domain-level ROBINS risk of bias judgements in this table.

Supplementary Table 9. **Judgements on the risk of bias assessment with modified ROBINS-I tool for seven domains and overall, for studies used in main and subgroup analysis[1]**

Study	Outcomes assessed[2]	1.Bias due to confounding	2.Bias in selection of participants into the study	3.Bias in classification of exposures	4. Bias due to departures from intended exposures	5.Bias due to missing data	6.Bias in measurement of outcomes	7.Bias in selection of the reported results	Overall judgement
Key 1999, AMS [8]	CVD, IHD, CBVD	Serious	Moderate	Serious	Moderate[3]	Low	Low	Moderate	Serious
Key 1999, AHS-1 [8]	CVD, IHD, CBVD	Serious	Moderate	Moderate	Moderate[3]	Low	Low	Moderate	Serious
Key 1999, HBS [8]	CBVD	Serious	Moderate	Serious	Moderate[3]	Low	Low	Moderate	Serious
Key 1999, pooled analysis (AMS, AHS-1, HBS, OVS) [8]	CVD, IHD, CBVD	Serious	Moderate	Serious	Moderate[3]	Low	Low	Moderate	Serious
Appleby 2002, OVS [7]	Circulatory disease, IHD, CBVD	Serious	Moderate	Serious	Moderate	Low	Low	Moderate	Serious
Appleby 2002, HFSS [7]	Circulatory disease, IHD, CBVD	Serious	Moderate	Serious	Moderate	Low	Low	Moderate	Serious
Chang-Claude, 2005, HBS [2]	Circulatory disease, IHD	Moderate	Moderate	Serious	Moderate	Low	Low	Moderate	Serious
Orlich 2013, AHS-2 [4]	CVD, IHD	Moderate	Moderate	Moderate	Moderate[3]	Low	Low	Moderate	Moderate
Tong 2019, EPIC-Oxford [6]	CVD, IHD, total stroke	Moderate	Low	Moderate	Low	Low	Low	Moderate	Moderate
Chiu 2020, TCHS [3]	Total stroke, ischemic stroke	Moderate	Moderate	Moderate	Moderate[3]	Low	Low	Moderate	Moderate
Chiu 2020, TCVS [3]	Total stroke, ischemic stroke, hemorrhagic stroke	Moderate	Moderate	Moderate	Moderate[3]	Low	Low	Moderate	Moderate
Petermann-Rocha 2021, UKB [5]	CVD, IHD, total stroke	Moderate	Low	Moderate	Moderate[3]	Low	Low	Moderate	Moderate
Baden 2021, NHS1 [1]	Total stroke	Moderate	Moderate	Moderate	Low	Low	Moderate	Moderate	Moderate
Baden 2021, NHS2 [1]	Total stroke	Moderate	Moderate	Moderate	Low	Low	Moderate	Moderate	Moderate
Baden 2021, HPFS [1]	Total stroke	Moderate	Moderate	Moderate	Low	Low	Moderate	Moderate	Moderate

Abbreviations: AHS-1 Adventist Health Study 1; AHS-2 Adventist Health Study 2; AMS Adventist Mortality Study; CBVD cerebrovascular disease; CVD cardiovascular disease; EPIC-Oxford European Prospective Investigation into Cancer and Nutrition - Oxford; HBS Heidelberg Study; HFSS Health Food Shoppers Study; HPFS Health Professionals Follow-up Study; IHD ischemic heart disease; NHS1 Nurses' Health Study 1; NHS2 Nurses' Health Study 2; OVS Oxford Vegetarian Study; TCHS Tzu Chi Health Study; TCVS Tzu Chi Vegetarian Study; UKB UK Biobank Study

[1] The ROBINS-I tool was modified in accordance with a similarly adapted version (preliminary ROBINS-E) of the instrument to overcome the issue that the tool was not originally developed for studies of exposure.

[2] The different outcomes listed have been assessed for risk of bias individually in each domain but have been grouped together as the authors did not find any difference in risk of bias between these outcomes.

[3] Studies that did not use a repeated FFQ, but any potential dietary changes that could have occurred during follow-up were considered to most likely only bias results toward the null.

Supplementary Table 10. **Judgements on risk of bias assessment with the ROBINS-I tool for seven domains and overall, for three additional publications used in subgroup analysis**

**Study**	**Outcomes assessed [1]**	**1.Bias due to confounding**	**2.Bias in selection of participants into the study**	**3.Bias in classification of exposures**	**4. Bias due to departures from intended exposures**	**5.Bias due to missing data**	**6.Bias in measurement of outcomes**	**7.Bias in selection of the reported results**	**Overall judgement**
Snowden 1984, AMS [12]	IHD	Serious	Moderate	Serious	Moderate[2]	Low	Low	Moderate	Serious
Fraser 1995, AHS-1 [13]	IHD	Serious	Moderate	Moderate	Moderate[2]	Low	Low	Moderate	Serious
Appleby 2016, pooled analysis (OVS, EPIC-Oxford) [14]	Circulatory disease, IHD, CBVD	Moderate	Moderate	Moderate	Low	Low	Low	Moderate	Moderate

Abbreviations: AHS-1 Adventist Health Study 1; AMS Adventist Mortality Study

[1] The different outcomes listed have been assessed for risk of bias individually in each domain but have been grouped together as the authors did not find any difference in risk of bias between these outcomes.

[2] Studies that did not use a repeated FFQ, but any potential dietary changes that could have occurred during follow-up were considered to most likely bias results toward the null.

References of cohort publications referred to in supplementary tables 8, 9 and 10

1. Baden MY, Shan Z, Wang F, Li Y, Manson JE, Rimm EB, Willett WC, Hu FB, Rexrode KM (2021) Quality of Plant-Based Diet and Risk of Total, Ischemic, and Hemorrhagic Stroke. Neurology 96 (15):e1940-e1953. doi:https://doi.org/10.1212/WNL.0000000000011713

2. Chang-Claude J, Hermann S, Eilber U, Steindorf K (2005) Lifestyle determinants and mortality in German vegetarians and health-conscious persons: results of a 21-year follow-up. Cancer Epidemiol Biomarkers Prev 14 (4):963-968. doi:https://doi.org/10.1158/1055-9965.EPI-04-0696

3. Chiu THT, Chang H-R, Wang L-Y, Chang C-C, Lin M-N, Lin C-L (2020) Vegetarian diet and incidence of total, ischemic, and hemorrhagic stroke in 2 cohorts in Taiwan. Neurology 94 (11):e1112-e1121. doi:https://doi.org/10.1212/WNL.0000000000009093

4. Orlich MJ, Singh PN, Sabaté J, Jaceldo-Siegl K, Fan J, Knutsen S, Beeson WL, Fraser GE (2013) Vegetarian dietary patterns and mortality in Adventist Health Study 2. JAMA Intern Med 173 (13):1230-1238. doi:10.1001/jamainternmed.2013.6473

5. Petermann-Rocha F, Parra-Soto S, Gray S, Anderson J, Welsh P, Gill J, Sattar N, Ho FK, Celis-Morales C, Pell JP (2021) Vegetarians, fish, poultry, and meat-eaters: who has higher risk of cardiovascular disease incidence and mortality? A prospective study from UK Biobank. Eur Heart J 42 (12):1136-1143. doi:https://doi.org/10.1093/eurheartj/ehaa939

6. Tong TYN, Appleby PN, Bradbury KE, Perez-Cornago A, Travis RC, Clarke R, Key TJ (2019) Risks of ischaemic heart disease and stroke in meat eaters, fish eaters, and vegetarians over 18 years of follow-up: Results from the prospective EPIC-Oxford study. BMJ 366:14897. doi:http://dx.doi.org/10.1136/bmj.l4897

7. Appleby PN, Key TJ, Thorogood M, Burr ML, Mann J (2002) Mortality in British vegetarians. Public Health Nutr 5 (1):29-36. doi:https://doi.org/10.1079/PHN2001248

8. Key TJ, Fraser GE, Thorogood M, Appleby PN, Beral V, Reeves G, Burr ML, Chang-Claude J, Frentzel-Beyme R, Kuzma JW, Mann J, McPherson K (1999) Mortality in vegetarians and nonvegetarians: detailed findings from a collaborative analysis of 5 prospective studies. Am J Clin Nutr 70 (3 Suppl):516S-524S. doi:https://doi.org/10.1093/ajcn/70.3.516s

9. Key TJ, Thorogood M, Appleby PN, Burr ML (1996) Dietary habits and mortality in 11 000 vegetarians and health conscious people: results of a 17 year follow up. BMJ 313 (7060):775-779. doi:https://doi.org/10.1136/bmj.313.7060.775

10. Bero L, Chartres N, Diong J, Fabbri A, Ghersi D, Lam J, Lau A, McDonald S, Mintzes B, Sutton P, Turton JL, Woodruff TJ (2018) The risk of bias in observational studies of exposures (ROBINS-E) tool: concerns arising from application to observational studies of exposures. Syst Rev 7 (1):242. doi:10.1186/s13643-018-0915-2

11. Fraser GE (1999) Associations between diet and cancer, ischemic heart disease, and all-cause mortality in non-Hispanic white California Seventh-day Adventists. Am J Clin Nutr 70 (3 Suppl):532S-538S. doi:https://doi.org/10.1093/ajcn/70.3.532s

12. Snowdon DA, Phillips RL, Fraser GE (1984) Meat consumption and fatal ischemia heart disease. Prev Med 13 (5):490-500. doi:http://dx.doi.org/10.1016/0091-7435%2884%2990017-3

13. Fraser GE, Lindsted KD, Beeson WL (1995) Effect of risk factor values on lifetime risk of and age at first coronary event. The Adventist Health Study. Am J Epidemiol 142 (7):746-758. doi:https://doi.org/10.1093/oxfordjournals.aje.a117706

14. Appleby PN, Crowe FL, Bradbury KE, Travis RC, Key TJ (2016) Mortality in vegetarians and comparable nonvegetarians in the United Kingdom. Am J Clin Nutr 103 (1):218-230. doi:https://doi.org/10.3945/ajcn.115.119461

Supplementary Table 11. Subgroup analysis of vegetarian vs. nonvegetarian diets and risk of cardiovascular disease

	Vegetarianism and cardiovascular disease				
	n	Relative risk (95% CI)	I^2 (%)	P_h[1]	P_h[2]
**All studies**	8	0.85 (0.79 - 0.92)	68	0.003	
**Sex**					
Men	3	0.83 (0.74 - 0.92)	42	0.18	0.09[3A]
Women	3	0.94 (0.89 - 0.99)	0	0.81	
Men, women	3	0.93 (0.85 - 1.03)	0	0.71	0.08[3B]
**Follow-up years**					
<10 years	3	0.84 (0.73 - 0.96)	77	0.013	0.79
≥10 years	5	0.86 (0.76 - 0.97)	69	0.011	
**Early follow-up years**					
Included	4	0.77 (0.68 - 0.86)	45	0.14	0.01
Excluded	4	0.92 (0.88 - 0.96)	0	0.64	
**Early follow-up years** [4]					
Included	3	0.92 (0.86 - 0.98)	0	0.77	0.79
Excluded	3	0.93 (0.87 - 1.00)	0	0.45	
**Incidence vs. mortality**					
Incidence	2	0.91 (0.87 - 0.95)	0	0.84	0.66
Mortality	7	0.86 (0.75 - 0.98)	79	0.000	
**Outcome type**					
Cardiovascular disease	5	0.82 (0.73-0.93)	74	0.01	0.12
Circulatory disease	5	0.94 (0.87-1.03)	55	0.07	
**Excluding prevalent disease at baseline**					
Yes	3	0.90 (0.86 - 0.95)	0	0.85	0.30
No	5	0.82 (0.71 - 0.94)	75	0.003	
**Geographic location**					
Europe	5	0.91 (0.87 - 0.95)	0	0.92	0.007

North America		3	0.76 (0.66 - 0.87)	61	0.08	
Asia		0				
**Number of cases**						
Cases <250		1	0.83 (0.62 - 1.12)	NC	NC	NC
Cases 250 – 499		1	0.93 (0.77 - 1.12)	NC	NC	
Cases ≥ 500		6	0.84 (0.77 - 0.92)	77	0.001	
**Risk of bias (ROBINS)**						
Bias due to confounding	Low	0				0.33
	Moderate	4	0.90 (0.86-0.94)	0	0.89	
	Serious	4	0.82 (0.69-0.96)	81	0.001	
Bias in selection of participants	Low	2	0.91 (0.87-0.95)	0	0.84	0.34
	Moderate	6	0.83 (0.73-0.93)	69	0.006	
	Serious	0				
Bias in classification of exposure	Low	0				0.93
	Moderate	4	0.85 (0.77 - 0.94)	76	0.006	
	Serious	4	0.85 (0.74 - 0.99)	67	0.03	
Bias due to departures from intended exposures	Low	2	0.81 (0.65 - 1.01)	85	0.01	0.58
	Moderate	6	0.87 (0.79 - 0.95)	65	0.01	
	Serious	0				
Bias due to missing data	Low	8	0.85 (0.79 - 0.92)	68	0.003	NC
	Moderate	0				
	Serious	0				
Bias in measurement of outcomes	Low	8	0.85 (0.79 - 0.92)	68	0.003	NC
	Moderate	0				
	Serious	0				
Bias in selection of the reported results	Low	0				NC
	Moderate	8	0.85 (0.79 - 0.92)	68	0.003	
	Serious	0				
Overall risk of bias	Low	0				0.30

	Moderate	3	0.90 (0.86 - 0.95)	0	0.85	
	Serious	5	0.82 (0.71 - 0.94)	75	0.003	
**Adjustment for confounding factors**						
Age	Yes	8	0.85 (0.79 - 0.92)	87	0.003	NC
	No	0				
Education	Yes	3	0.89 (0.83 - 0.96)	0	0.84	0.66
	No	5	0.84 (0.74 - 0.95)	81	0.000	
Alcohol	Yes	4	0.90 (0.86 - 0.94)	0	0.89	0.33
	No	4	0.82 (0.69 - 0.96)	81	0.001	
Smoking	Yes	8	0.85 (0.79 - 0.92)	87	0.003	NC
	No	0				
BMI	Yes	3	0.91 (0.87 - 0.96)	0	0.75	0.32
	No	5	0.83 (0.72 - 0.94)	75	0.003	
BMI[5]	Yes	2	0.92 (0.87 - 0.96)	0	0.68	0.18
	No	2	0.86 (0.81 - 0.92)	41	0.19	
Physical activity	Yes	4	0.90 (0.86 - 0.94)	0	0.89	0.33
	No	4	0.82 (0.69 - 0.96)	81	0.001	

Abbreviations: NC not calculable because no studies were present in one of the subgroups; ROBINS-I Risk Of Bias In Non-randomized Studies of Interventions;

n denotes the number of risk estimates or observations included in each subgroup analysis (some publications reported RRs for more than one study)

Ph[1] for heterogeneity within each subgroup

Ph[2] for heterogeneity between subgroups with meta-regression analysis

[3A] P for heterogeneity between men, women and men/women combined with meta-regression analysis

[3B] P for heterogeneity between men and women (excluding studies with both sexes combined) with meta-regression analysis

[4] Restricted to studies reporting results with both inclusion of early follow-up and exclusion of

early follow-up (two risk estimates included in this subgroup analysis for each relevant study)

[5] Restricted to studies reporting both BMI-adjusted and BMI-unadjusted results (two risk estimates included in this subgroup analysis for each relevant study)

Supplementary Table 12. Subgroup analysis of vegetarian vs. nonvegetarian diets and risk of ischemic heart disease and total stroke

	**Vegetarianism and ischemic heart disease**					**Vegetarianism and total stroke**				
	n	Relative risk (95% CI)	I^2 (%)	$P_h{}^1$	$P_h{}^2$	*n*	Relative risk (95% CI)	I^2 (%)	$P_h{}^1$	$P_h{}^2$
**All studies**	8	0.79 (0.71 - 0.88)	67	0.003		12	0.90 (0.77 - 1.05)	61	0.003	
**Sex**										
Men	5	0.74 (0.63 - 0.86)	63	0.03	0.24[3A]	3	0.92 (0.75 - 1.14)	0	0.70	0.46[3A]
Women	5	0.86 (0.74 - 0.99)	67	0.02	0.19[3B]	4	1.05 (0.83 - 1.33)	47	0.13	
Men, women	3	0.84 (0.73 - 0.97)	0	0.78		7	0.83 (0.66 - 1.04)	62	0.015	0.39[3B]
**Follow-up**										
<10 years	3	0.84 (0.70 - 1.00)	71	0.03	0.38	5	0.71 (0.54 - 0.94)	52	0.08	0.01
≥10 years	5	0.76 (0.67 - 0.86)	55	0.06		7	1.06 (0.96 - 1.18)	0	0.55	
**Early follow-up years**										
Included	4	0.71 (0.62 - 0.80)	28	0.25	0.04	8	0.80 (0.64 - 0.99)	48	0.06	0.13
Excluded	4	0.89 (0.80 - 0.99)	46	0.14		4	1.02 (0.86 - 1.21)	48	0.125	
**Early follow-up years [4]**										
Included	3	0.81 (0.74 - 0.88)	0	0.62	0.60	3	1.12 (0.99 - 1.27)	0	0.38	0.96
Excluded	3	0.84 (0.76 - 0.93)	6	0.34		3	1.11 (0.97 - 1.27)	0	0.42	
**Incidence vs. mortality**										

Incidence	2	0.87 (0.71 - 1.06)	85	0.01	0.48	7	0.85 (0.66 - 1.09)	69	0.004	0.55
Mortality	7	0.79 (0.69-0.90)	58	0.03		6	0.95 (0.78-1.17)	57	0.04	
**Outcome type**										
Total stroke						7	0.85 (0.66-1.09)	69	0.004	0.66
Cerebrovascular disease						5	0.92 (0.75-1.13)	51	0.09	
**Stroke subtype**										
Ischemic stroke						3	0.56 (0.22 - 1.42)	82	0.004	0.67
Hemorrhagic stroke						2	0.77 (0.19 - 3.09)	85	0.01	
**Excluding prevalent disease at baseline**										
Yes	3	0.85 (0.73 - 0.99)	71	0.03	0.25	7	0.85 (0.66 - 1.09)	69	0.004	0.66
No	5	0.75 (0.65 - 0.86)	53	0.08		5	0.92 (0.75 - 1.13)	51	0.09	
**Geographic location**										
Europe	5	0.85 (0.77 - 0.95)	44	0.13	0.09	5	1.05 (0.89 - 1.23)	45	0.12	0.01
North America	3	0.71 (0.61 - 0.83)	52	0.13		5	0.85 (0.71 - 1.03)	22	0.27	
Asia	0					2	0.52 (0.35 - 0.76)	0	0.96	
**Number of cases**										
Cases <250	1	0.70 (0.41 - 1.19)	NC	NC	0.97	5	0.75 (0.52 - 1.09)	65	0.02	0.13
Cases 250 – 499	2	0.83 (0.70 - 0.99)	0	0.74		2	0.96 (0.82 - 1.14)	0	0.71	
Cases ≥ 500	5	0.79 (0.68 - 0.91)	81	0.000		5	1.00 (0.83 - 1.21)	43	0.13	

**Risk of bias (ROBINS)**											
Bias due to confounding	Low	0				0.32	0				0.66
	Moderate	4	0.84 (0.73-0.97)	59	0.06		7	0.85 (0.66-1.09)	69	0.004	
	Serious	4	0.75 (0.64-0.88)	64	0.04		5	0.92 (0.75-1.13)	51	0.09	
Bias in selection of participants	Low	2	0.86 (0.71-1.06)	85	0.01		2	1.02 (0.72-1.44)	83	0.02	
	Moderate	6	0.76 (0.67-0.85)	44	0.11		10	0.86 (0.72-1.02)	47	0.05	
	Serious	0					0				
Bias in classification of exposures	Low	0				0.91	0				0.73
	Moderate	4	0.79 (0.65-0.94)	85	0.00		8	0.88 (0.71-1.07)	64	0.007	
	Serious	4	0.80 (0.72-0.89)	0	0.61		4	0.93 (0.70-1.26)	63	0.04	
Bias due to departures from intended exposures	Low	4	0.84 (0.73-0.97)	59	0.06	0.67	5	0.92 (0.69-1.23)	71	0.008	0.70
	Moderate	4	0.75 (0.64-0.88)	64	0.04		7	0.87 (0.72-1.06)	50	0.06	
	Serious	0					0				
Bias due to missing data	Low	8	0.79 (0.71 - 0.88)	67	0.003	NC	8	0.90 (0.77-1.05)	61	0.003	NC
	Moderate	0					0				
	Serious	0					0				
Bias in measurement of outcomes	Low	8	0.79 (0.71-0.88)	67	0.003	NC	9	0.88 (0.73-1.07)	71	0.001	0.79
	Moderate	0					3	0.96 (0.73-1.27)	0	0.71	
	Serious	0					0				

Bias in selection of the reported results	Low	0				NC	0				NC
	Moderate	8	0.79 (0.71-0.88)	67	0.003		12	0.90 (0.77-1.05)	61	0.003	
	Serious	0					0				
Overall risk of bias	Low	0				0.25	0				0.66
	Moderate	3	0.85 (0.73-0.99)	71	0.03		7	0.85 (0.66-1.09)	69	0.004	
	Serious	5	0.75 (0.65-0.86)	53	0.08		5	0.92 (0.75-1.13)	51	0.09	
**Adjustment for confounding factors**											
Age	Yes	8	0.79 (0.71 - 0.89)	67	0.003	NC	12	0.90 (0.77 - 1.05)	61	0.003	NC
	No	0					0				
Education	Yes	3	0.78 (0.71 - 0.86)	0	0.88	0.86	3	0.72 (0.36 - 1.41)	87	0.000	0.55
	No	5	0.80 (0.67 - 0.94)	81	0.000		9	0.91 (0.80 - 1.03)	16	0.31	
Alcohol	Yes	4	0.84 (0.73 - 0.97	59	0.06	0.32	7	0.85 (0.66 - 1.09)	69	0.004	0.66
	No	4	0.75 (0.64 - 0.88)	64	0.04		5	0.92 (0.75 - 1.13)	51	0.09	
Smoking	Yes	8	0.79 (0.71 - 0.89)	87	0.003	NC	12	0.90 (0.77 - 1.05)	61	0.003	NC
	No	0					0				
BMI	Yes	3	0.82 (0.68-0.98)	83	0.002	0.92	6	0.86 (0.68 - 1.08)	63	0.02	0.56
	No	2	0.84 (0.73-0.96)	0	0.75		6	0.95 (0.77 - 1.18)	52	0.06	
BMI[5]	Yes	3	0.82 (0.68-0.98)	83	0.002	0.73	2	1.02 (0.71 - 1.46)	84	0.01	0.96
	No	3	0.78 (0.66-0.92)	79	0.008		2	1.00 (0.69 - 1.46)	85	0.01	

Physical activity	Yes	4	0.84 (0.73 - 0.97)	59	0.06	0.32	7	0.85 (0.66 - 1.09)	69	0.004	0.66
	No	4	0.75 (0.64 - 0.88)	64	0.04		5	0.92 (0.75 - 1.13)	51	0.09	

Abbreviations: NC not calculable because no studies were present in one of the subgroups; ROBINS-I Risk Of Bias In Non-randomized Studies of Interventions;

n denotes the number of risk estimates or observations included in subgroup analysis (some publications reported risk estimates for more than one study)

Ph[1] for heterogeneity within each subgroup

Ph[2] for heterogeneity between subgroups with meta-regression analysis

[3A] P for heterogeneity between men, women and men/women combined with meta-regression analysis

[3B] P for heterogeneity between men and women (excluding studies with both sexes combined) with meta-regression analysis

[4] Restricted to studies reporting results with both inclusion of early follow-up and exclusion of early follow-up (two risk estimates included in this subgroup analysis for each relevant study)

[5] Restricted to studies reporting both BMI-adjusted and BMI-unadjusted results (two risk estimates included in this subgroup analysis for each relevant study)

Supplementary Table 13. Justification for evidence grading for vegetarian diets and cardiovascular disease, ischemic heart disease and stroke [1,2]

Requirements for grading of convincing	Cardiovascular disease	Ischemic heart disease	Stroke
Statistically significant and robust association	Statistically significant moderate inverse association for vegetarians vs. non-vegetarians which is robust in influence analyses.	Statistically significant moderate inverse association for vegetarians vs. non-vegetarians which is robust in influence analyses.	No statistically significant inverse association for vegetarians vs. non-vegetarians, however, in influence analyses, there is a suggestive inverse association when one study (EPIC-Oxford) is excluded, suggesting that this finding is not robust.
Evidence from at least two independent cohort studies	8 cohort studies	8 cohort studies	12 cohort studies
No substantial unexplained	There is heterogeneity in the overall analysis, however, this is	There is heterogeneity in the overall analysis, however, this is	There is heterogeneity in the overall analysis and there is some

heterogeneity within or between study types or in different populations relating to the presence or absence of an association, or direction of effect	more due to differences in the effect sizes as all studies show risk estimates in the direction of reduced risk.	more due to differences in the effect sizes as all studies show risk estimates in the direction of reduced risk.	heterogeneity in the direction of the observed effect sizes with two studies reporting RRs of 1.2 or higher (one statistically significant), three studies reporting RRs close to 1.0 (between 0.9-1.1), and four studies reporting RRs of <0.85 (2 statistically significant)
Good quality studies to exclude with confidence the possibility that the observed association results from random or systematic error, including confounding,	No indication of publication bias<br><br>Results persisted in nearly all subgroup analyses. Results persisted in subgroup analyses of studies that adjusted for age, education, smoking, alcohol, BMI, and physical activity.	No indication of publication bias<br><br>Results persisted in nearly all subgroup analyses. Results persisted in subgroup analyses of studies that adjusted for age, education, smoking, alcohol, BMI, and physical activity.	No indication of publication bias.<br><br>Most subgroup analyses show no statistically significant association. One exception is the analysis stratified by geographic location which shows a significant inverse association in Asian

measurement error, and selection bias	Results persisted in subgroup analyses excluding participants with prevalent disease. Exposed and non-exposed participants were selected from the same populations.	Results were slightly weaker (but persisted) in subgroup analyses excluding participants with prevalent disease. One study showed similar results when using baseline or updated dietary data. Exposed and non-exposed participants were selected from the same populations.	studies, while European and American studies show no clear association.<br><br>One study showed similar results when using baseline or updated dietary data. Exposed and non-exposed participants were selected from the same populations.
Presence of a plausible biological gradient in the association. Such a gradient need not be linear or even in the same direction across different	Not applicable as the exposure is dichotomous	Not applicable as the exposure is dichotomous	Not applicable as the exposure is dichotomous

levels of exposure, so long as this can be explained plausibly			
Strong and plausible experimental evidence, either from human studies or relevant animal models, that typical human exposures can lead to relevant outcomes	There is strong evidence from randomized trials that vegetarian diets reduce cardiovascular risk factors including LDL cholesterol, systolic and diastolic blood pressure, and weight gain, and there is strong evidence from cohort studies that vegetarian diets reduce risk of type 2 diabetes.<br><br>Evidence regarding certain food groups that usually differ	There is strong evidence from randomized trials that vegetarian diets reduce cardiovascular risk factors including LDL cholesterol, systolic and diastolic blood pressure, and weight gain, and there is strong evidence from cohort studies that vegetarian diets reduce risk of type 2 diabetes.<br><br>Evidence regarding certain food groups that usually differ	There is strong evidence from randomized trials that vegetarian diets reduce cardiovascular risk factors including LDL cholesterol, systolic and diastolic blood pressure, and weight gain, and there is strong evidence from cohort studies that vegetarian diets reduce risk of type 2 diabetes.<br><br>Evidence regarding certain food groups that usually differ importantly between vegetarians and non-

	importantly between vegetarians and non-vegetarians are also consistent with the observed results. Red and processed meat are consistently associated with increased CVD risk, while intake of fruits, vegetables, whole grains, and nuts have been consistently associated with lower CVD risk. Biologically plausible mechanisms exist by which these foods influence CVD risk.	importantly between vegetarians and non-vegetarians are also consistent with the observed results. Red and processed meat are consistently associated with increased IHD risk, while intake of fruits, vegetables, whole grains, legumes, and nuts have been consistently associated with lower IHD risk. Biologically plausible mechanisms exist by which these foods influence IHD risk.	vegetarians are also consistent with the observed results. Red and processed meat have been associated with increased stroke risk in some, but not all studies, while intake of fruits, vegetables, whole grains, and nuts have been consistently associated with lower stroke risk. Biologically plausible mechanisms exist by which these foods influence stroke risk.
Final grading and justification for overall assessment.	Probable evidence that vegetarian diets reduce cardiovascular disease risk.	Probable evidence that vegetarian diets reduce ischemic heart disease risk.	Limited-no conclusion evidence that vegetarian diets are associated with risk of stroke.

	Justification: Statistically significant 15% reduction in risk based on 8 cohort studies. Results are robust in influence analyses and consistent across most subgroup analyses. Although there is heterogeneity, this is driven by differences in the strength (not direction) of the association. There is no indication of publication bias. Randomized trials provide ample evidence that vegetarian diets reduce cardiovascular risk factors including LDL-cholesterol, systolic and diastolic blood	Justification: Statistically significant 21% reduction in risk based on 8 cohort studies. Results are robust in influence analyses and consistent across most subgroup analyses. Although there is heterogeneity, this is driven by differences in the strength (not direction) of the association. There is no indication of publication bias. Randomized trials provide ample evidence that vegetarian diets reduce cardiovascular risk factors including LDL-cholesterol, systolic and diastolic blood	Justification: No statistically significant association overall, based on 12 cohort studies. The overall lack of a statistically significant association is the primary reason for this judgement as there are mechanistic data and data on food groups that would point towards an inverse association. Results are not robust in influence analyses and exclusion of one study shows a marginally significant inverse association. The null results are consistent across most subgroup analyses, but one exception is geographic location, where there is a statistically significant inverse

	pressure, weight gain and cohorts show inverse associations with type 2 diabetes risk. Results for several food groups that differ between vegetarians and non-vegetarians are consistent with the observed associations and biologically plausible mechanisms also exist for these.	pressure, weight gain and cohorts show inverse associations with type 2 diabetes risk. Results for several food groups that differ between vegetarians and non-vegetarians are consistent with the observed associations and biologically plausible mechanisms also exist for these.	association in Asian studies, but not in European or American studies. There is heterogeneity and this is also due to differences in the direction of the association. There is no indication of publication bias. Randomized trials provide ample evidence that vegetarian diets reduce cardiovascular risk factors including LDL-cholesterol, systolic and diastolic blood pressure, weight gain and cohorts show inverse associations with type 2 diabetes risk. Results for several food groups that differ between vegetarians and non-vegetarians are consistent with the observed associations and biologically

			plausible mechanisms also exist for these.

[1]Few studies were published on vegans and results showed an imprecisely estimated inverse association for IHD and null results for CVD and total stroke. For this reason, the judgement was considered limited-suggestive for IHD and limited-no conclusion for CVD and stroke

[2]Few studies were published on vegetarian diets and subtypes of stroke and the results showed no clear associations for either outcome as the confidence intervals were wide.

Supplementary Table 14. Evidence grading for vegetarian or vegan vs. nonvegetarian diets and cardiovascular disease, ischemic heart disease and stroke

	Reduced risk	Increased risk
Convincing	-	-
Probable	Cardiovascular disease (vegetarian diets)<br><br>Ischemic heart disease (vegetarian diets)	-
Limited-suggestive	Ischemic heart disease (vegan diets)	-
Limited - no conclusion	Total stroke, ischemic stroke, hemorrhagic stroke (vegetarian diets, vegan diets)<br><br>Cardiovascular disease (vegan diets)	

Supplementary Figure 1. Risk of bias judgements for seven domains and overall, for studies used in main analysis of vegetarian vs. nonvegetarian diets and cardiovascular disease using a modified ROBINS tool [1, 2]

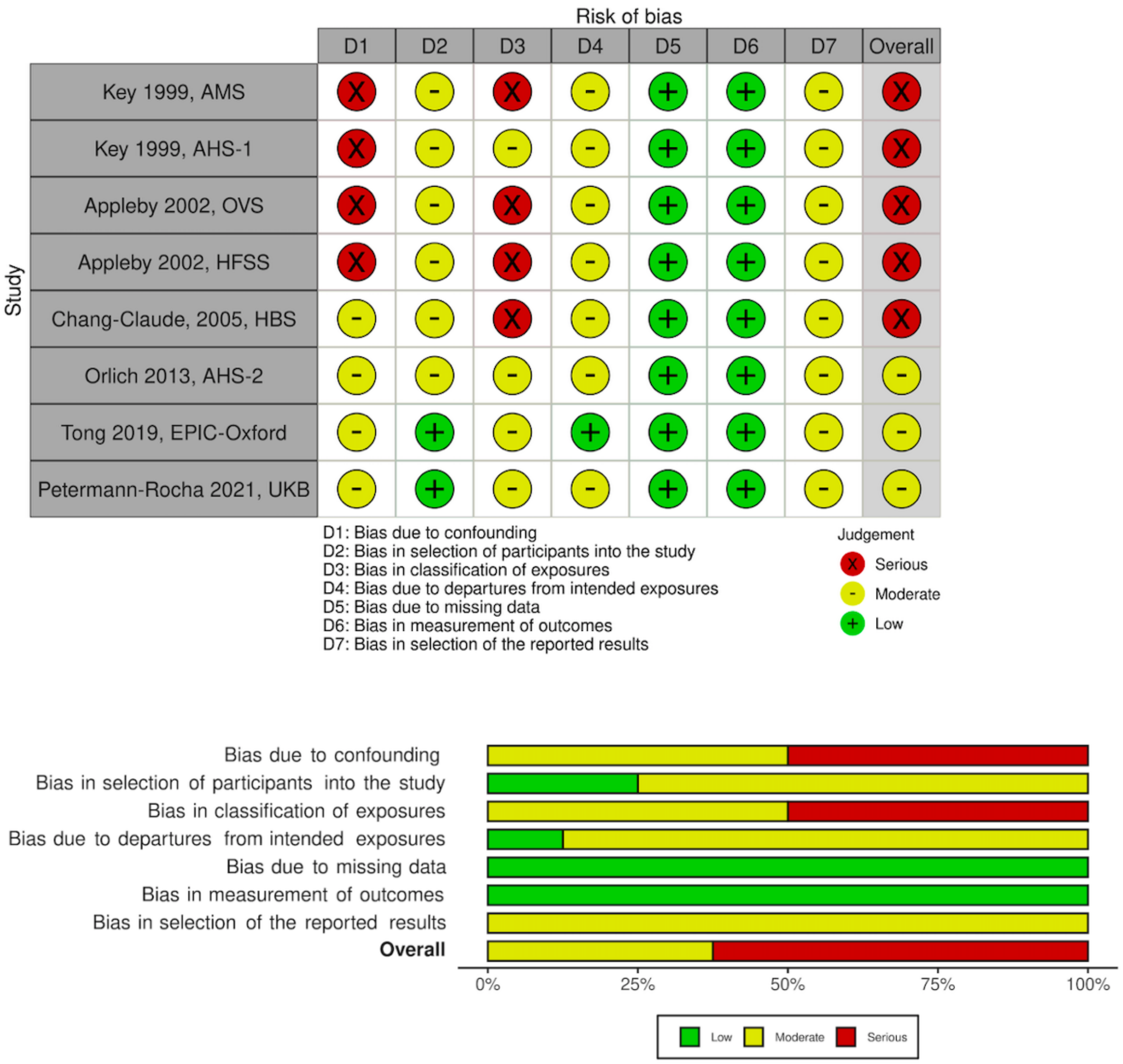

From: McGuinness LA, Higgins JPT (2021) Risk-of-bias VISualization (robvis): An R package and Shiny web app for visualizing risk-of-bias assessments. Res Synth Methods 12 (1):55-61. doi: 10.1002/jrsm.1411. Epub 2020 May 6.

Abbreviations: AHS-1 Adventist Health Study 1; AHS-2 Adventist Health Study 2; AMS Adventist Mortality Study; EPIC-Oxford European Prospective Investigation into Cancer and Nutrition - Oxford; HBS Heidelberg Study; HFSS Health Food Shoppers Study;

OVS Oxford Vegetarian Study; RoB Risk of Bias; ROBINS-I Risk Of Bias In Non-randomized Studies of Interventions; UKB UK Biobank Study

[1] The ROBINS-I tool was modified in accordance with a similarly adapted tool (preliminary ROBINS-E) to better assess risk of bias in studies of exposure.

[2] Each domain was evaluated for the direction and magnitude of the potential for risk of bias (RoB) to assess whether there were obvious additive effects between the domains e.g., as two domains judged ‘serious’ RoB or two domains judged ‘moderate’ RoB could in theory equate a higher risk of bias (critical and moderate RoB respectively), however, this potential was not recognized in the study material.

Supplementary Figure 2. Risk of bias judgements for seven domains and overall, for studies used in main analysis of vegetarian vs. nonvegetarian diets and ischemic heart disease using a modified ROBINS tool [1, 2]

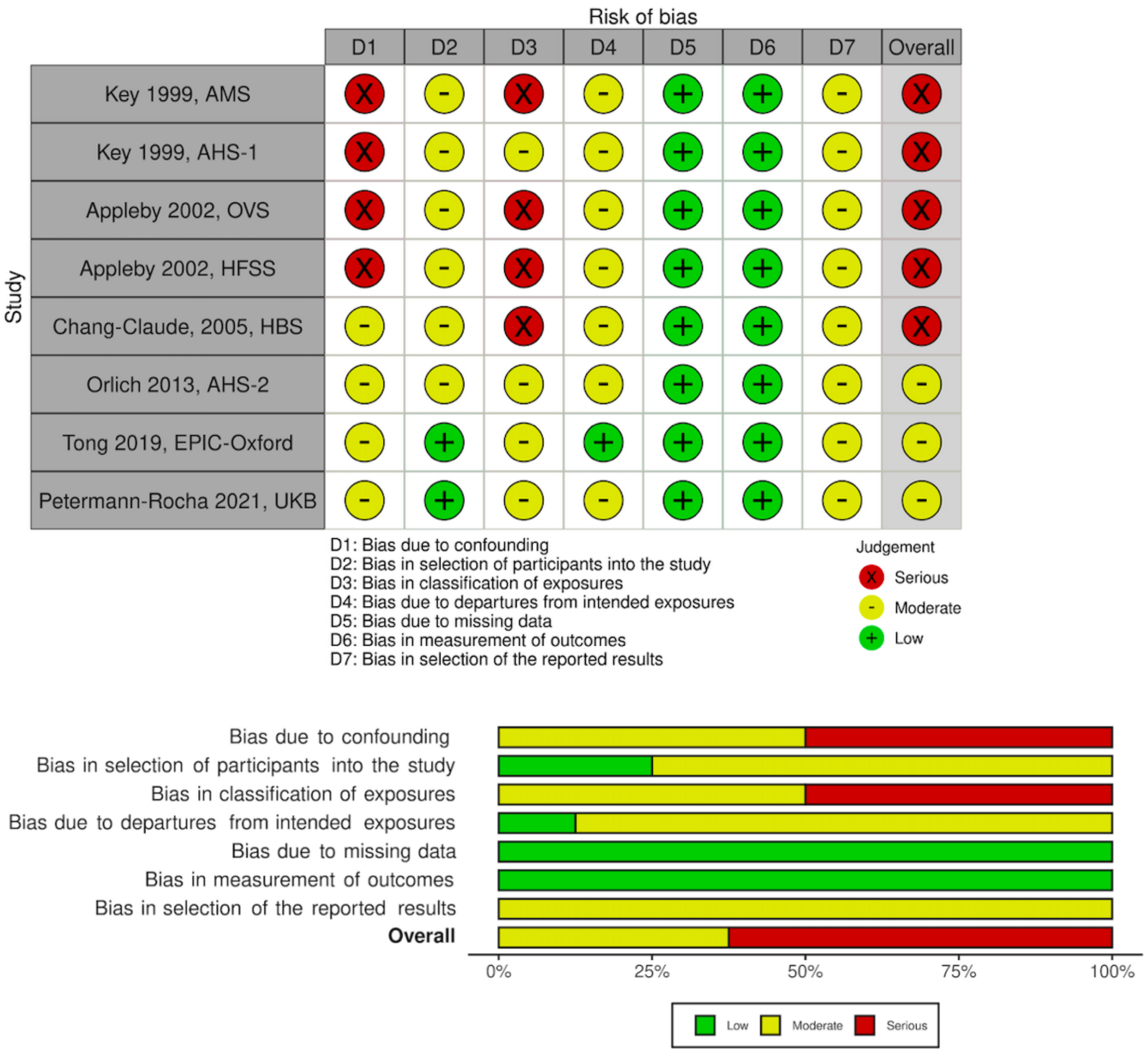

From: McGuinness LA, Higgins JPT (2021) Risk-of-bias VISualization (robvis): An R package and Shiny web app for visualizing risk-of-bias assessments. Res Synth Methods 12 (1):55-61. doi: 10.1002/jrsm.1411. Epub 2020 May 6.

Abbreviations: AHS-1 Adventist Health Study 1; AHS-2 Adventist Health Study 2; AMS Adventist Mortality Study; EPIC-Oxford European Prospective Investigation into Cancer and Nutrition - Oxford; HBS Heidelberg Study; HFSS Health Food Shoppers Study;

OVS Oxford Vegetarian Study; RoB Risk of Bias; ROBINS-I Risk Of Bias In Non-randomized Studies of Interventions; UKB UK Biobank Study

[1] The ROBINS-I tool was modified in accordance with a similarly adapted tool (preliminary ROBINS-E) to better assess risk of bias in studies of exposure.

[2] Each domain was evaluated for the direction and magnitude of the potential for risk of bias (RoB) to assess whether there were obvious additive effects between the domains e.g., as two domains judged 'serious' RoB or two domains judged 'moderate' RoB could in theory equate a higher RoB (critical and moderate RoB respectively), however, this potential was not recognized in the study material.

Supplementary Figure 3. Risk of bias judgements for seven domains and overall, for studies used in main analysis of vegetarian vs. nonvegetarian diets and total stroke using a modified ROBINS tool [1, 2]

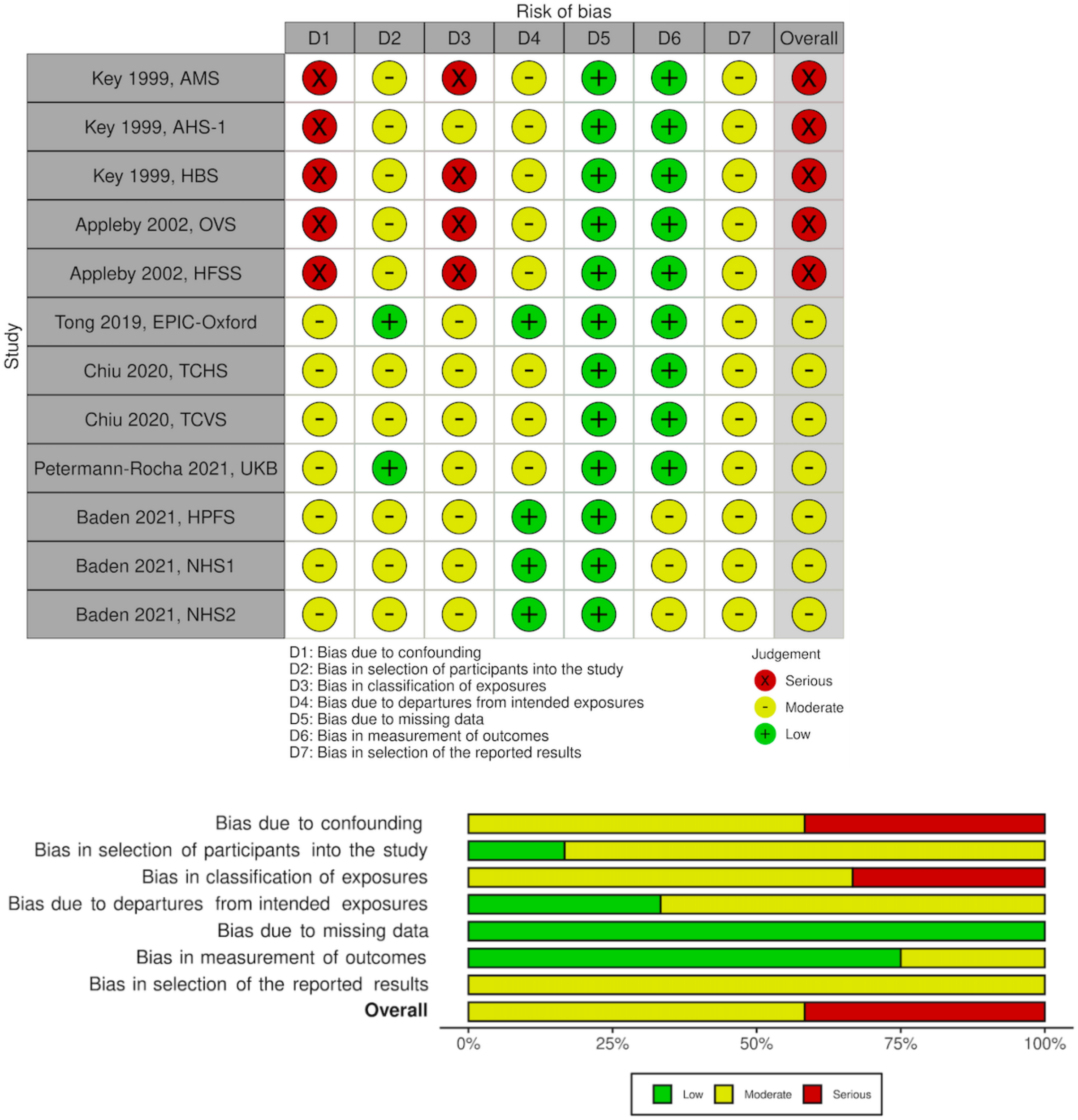

From: McGuinness LA, Higgins JPT (2021) Risk-of-bias VISualization (robvis): An R package and Shiny web app for visualizing risk-of-bias assessments. Res Synth Methods 12 (1):55-61. Doi: 10.1002/jrsm.1411. Epub 2020 May 6.

Abbreviations: AHS-1 Adventist Health Study 1; AMS Adventist Mortality Study; EPIC-Oxford European Prospective Investigation into Cancer and Nutrition - Oxford; HBS

Heidelberg Study; HFSS Health Food Shoppers Study; HPFS Health Professionals Follow-up Study; NHS1 Nurses' Health Study 1; NHS2 Nurses' Health Study 2; OVS Oxford Vegetarian Study; RoB Risk of Bias; ROBINS-I Risk Of Bias In Non-randomized Studies of Interventions; TCHS Tzu Chi Health Study; TCVS Tzu Chi Vegetarian Study; UKB UK Biobank Study

[1] The ROBINS-I tool was modified in accordance with a similarly adapted tool (preliminary ROBINS-E) to better assess risk of bias in studies of exposure.

[2] Each domain was evaluated for the direction and magnitude of the potential for risk of bias (RoB) to assess whether there were obvious additive effects between the domains e.g., as two domains judged 'serious' RoB or two domains judged 'moderate' RoB could in theory equate a higher RoB (critical and moderate RoB respectively), however, this potential was not recognized in the study material.

Supplementary Figure 4. Funnel plot of publication bias analysis of vegetarian vs. nonvegetarian diets and cardiovascular disease

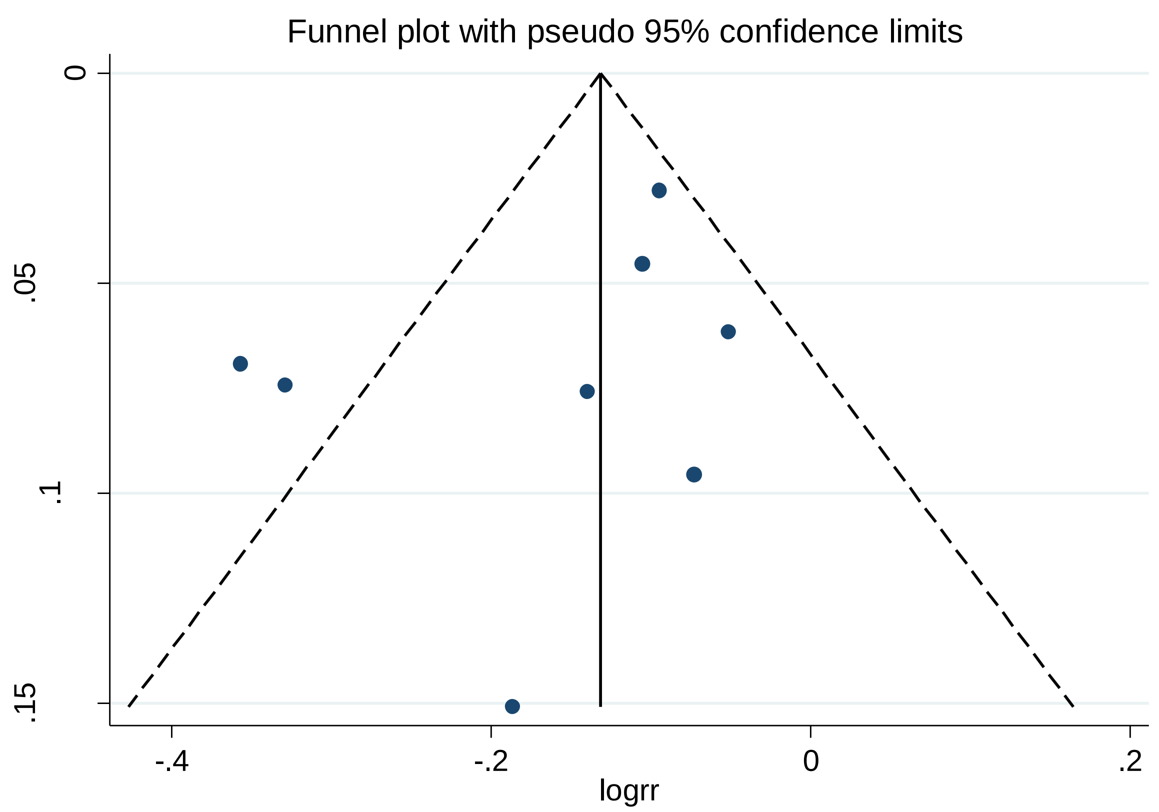

Egger's test: p=0.28

Begg's test: p=0.39

Supplementary Figure 5. Influence analysis of vegetarian vs. nonvegetarian diets and cardiovascular disease

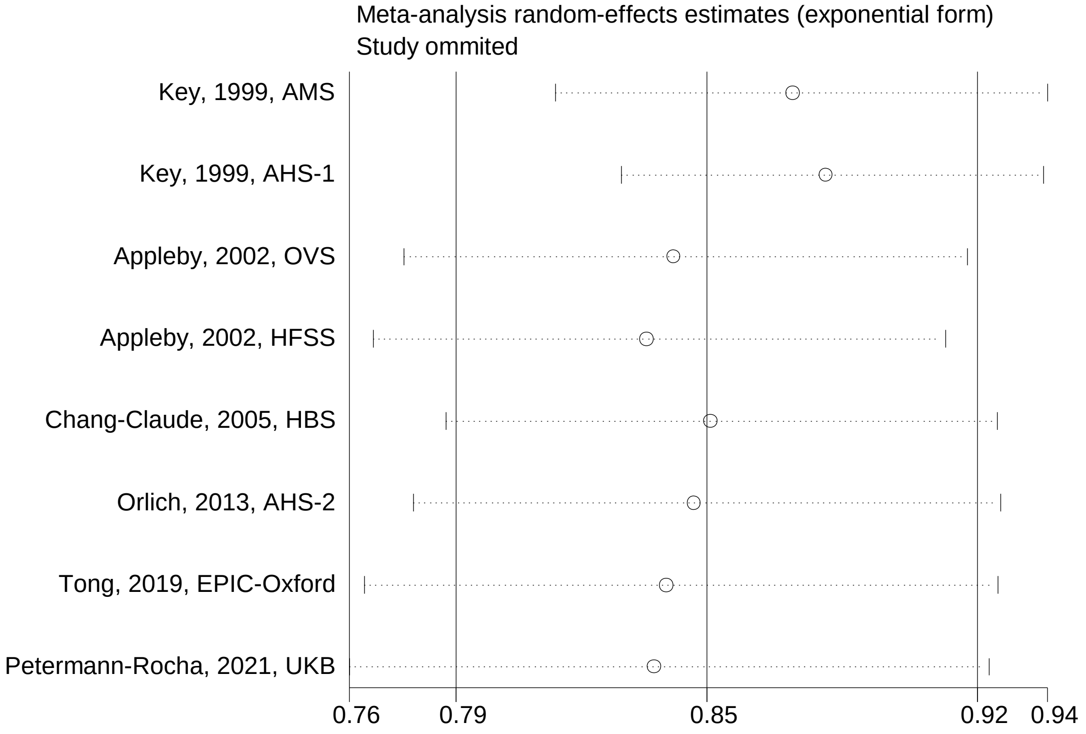

Study omitted	e^coef.	[95% Conf.	Interval]	I^2 (%)
Key, 1999, AHS-1	0.88271922	0.83081025	0.93787146	42
Key, 1999, AMS	0.87424785	0.81400406	0.93895024	58
Appleby, 2002, HFSS	0.83723617	0.76775843	0.91300118	70
Appleby, 2002, OVS	0.84407473	0.77565116	0.91853422	72
Chang-Claude, 2005, HBS	0.8533538	0.78626084	0.92617196	72
Orlich, 2013, AHS-2	0.8492139	0.77797532	0.92697573	73
Tong, 2019, EPIC-Oxford	0.84215099	0.7655769	0.92638415	72
Petermann-Rocha, 2021, UKB	0.83901322	0.76174772	0.92411596	68
Combined	0.85249322	0.78895491	0.92114858	68

Abbreviations: AHS-1 Adventist Health Study 1; AHS-2 Adventist Health Study 2; AMS Adventist Mortality Study; CVD cardiovascular disease; EPIC-Oxford European Prospective Investigation into Cancer and Nutrition - Oxford; HBS Heidelberg Study; HFSS Health Food Shoppers Study; OVS Oxford Vegetarian Study; UKB UK Biobank Study

Supplementary Figure 6. Funnel plot of publication bias analysis of vegetarian vs. nonvegetarian diets and ischemic heart disease

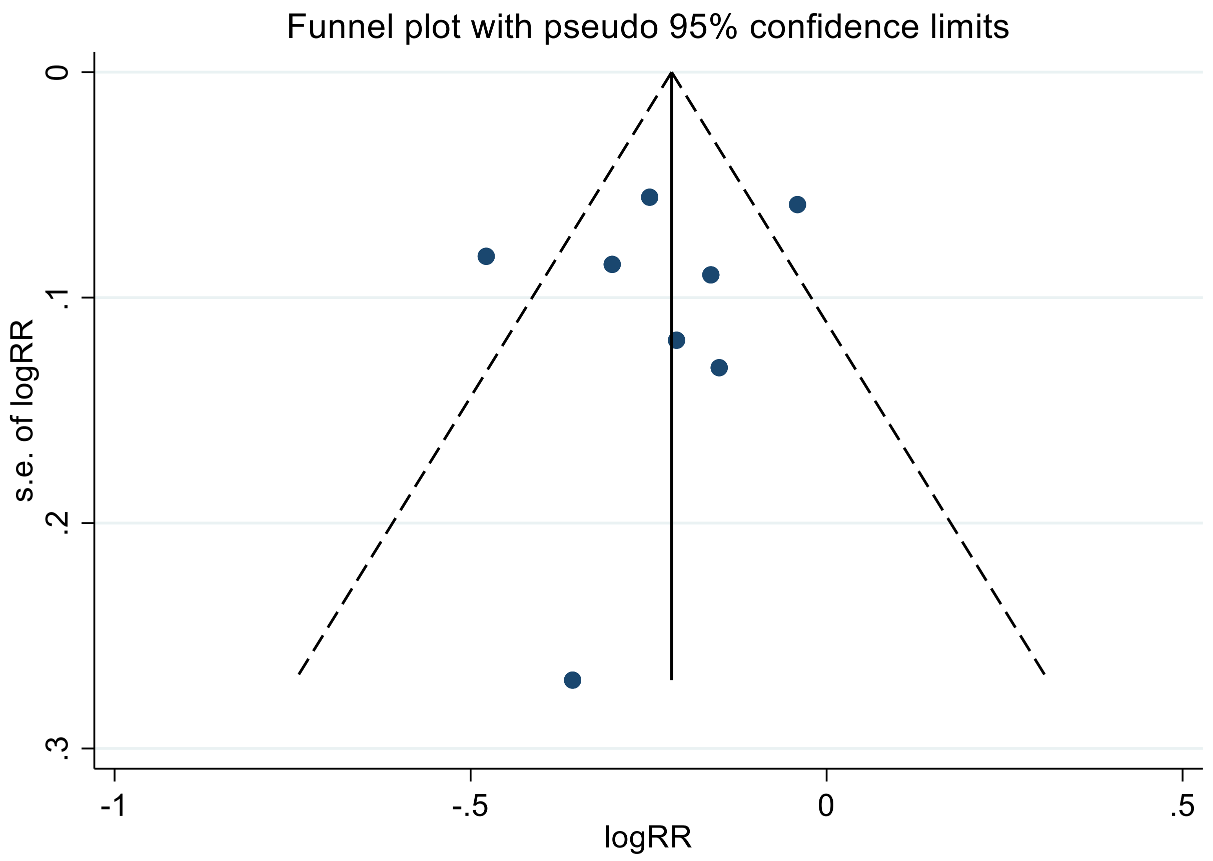

Egger's test: p=0.61

Begg's test: p=0.90

Supplementary Figure 7. Influence analysis of vegetarian diet and ischemic heart disease

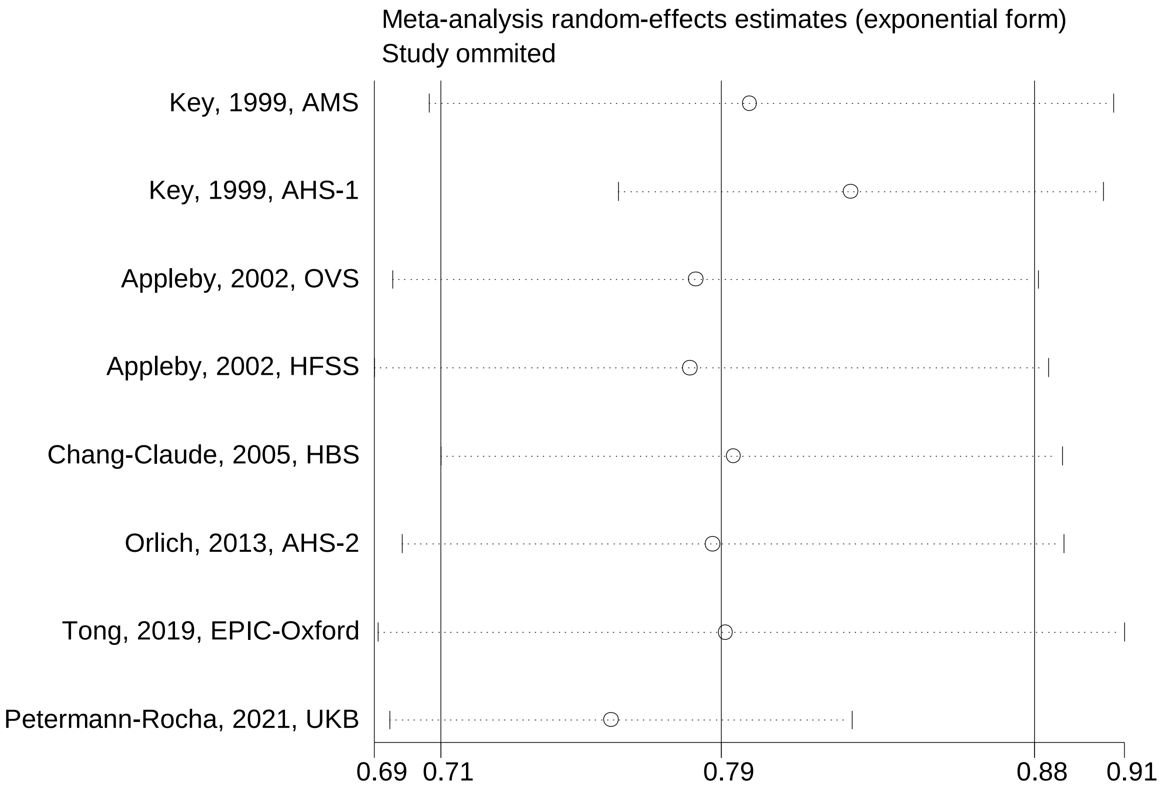

Study omitted	e^coef.	[95% Conf.	Interval]	I^2 (%)
Key, 1999, AMS	0.80089068	0.70663166	0.90772307	71
Key, 1999, AHS-1	0.83040214	0.76221234	0.90469241	38
Appleby, 2002, OVS	0.78504151	0.69584066	0.8856771	72
Appleby, 2002, HFSS	0.78328973	0.69044214	0.88862312	71
Chang-Claude, 2005, HBS	0.79611307	0.7100206	0.89264452	72
Orlich, 2013, AHS-2	0.789931	0.69870925	0.89306241	72
Tong, 2019, EPIC-Oxford	0.7937426	0.6915859	0.91098928	71
Petermann-Rocha, 2021, UKB	0.75995785	0.6950562	0.8309198	36
Combined	0.79251781	0.71009865	0.88450312	67

Abbreviations: AHS-1 Adventist Health Study 1; AHS-2 Adventist Health Study 2; AMS Adventist Mortality Study; EPIC-Oxford European Prospective Investigation into Cancer and Nutrition - Oxford; HBS Heidelberg Study; HFSS Health Food Shoppers Study; IHD ischemic heart disease; OVS Oxford Vegetarian Study; UKB UK Biobank Study

Supplementary Figure 8. Funnel plot of publication bias analysis of vegetarian vs. nonvegetarian diets and total stroke

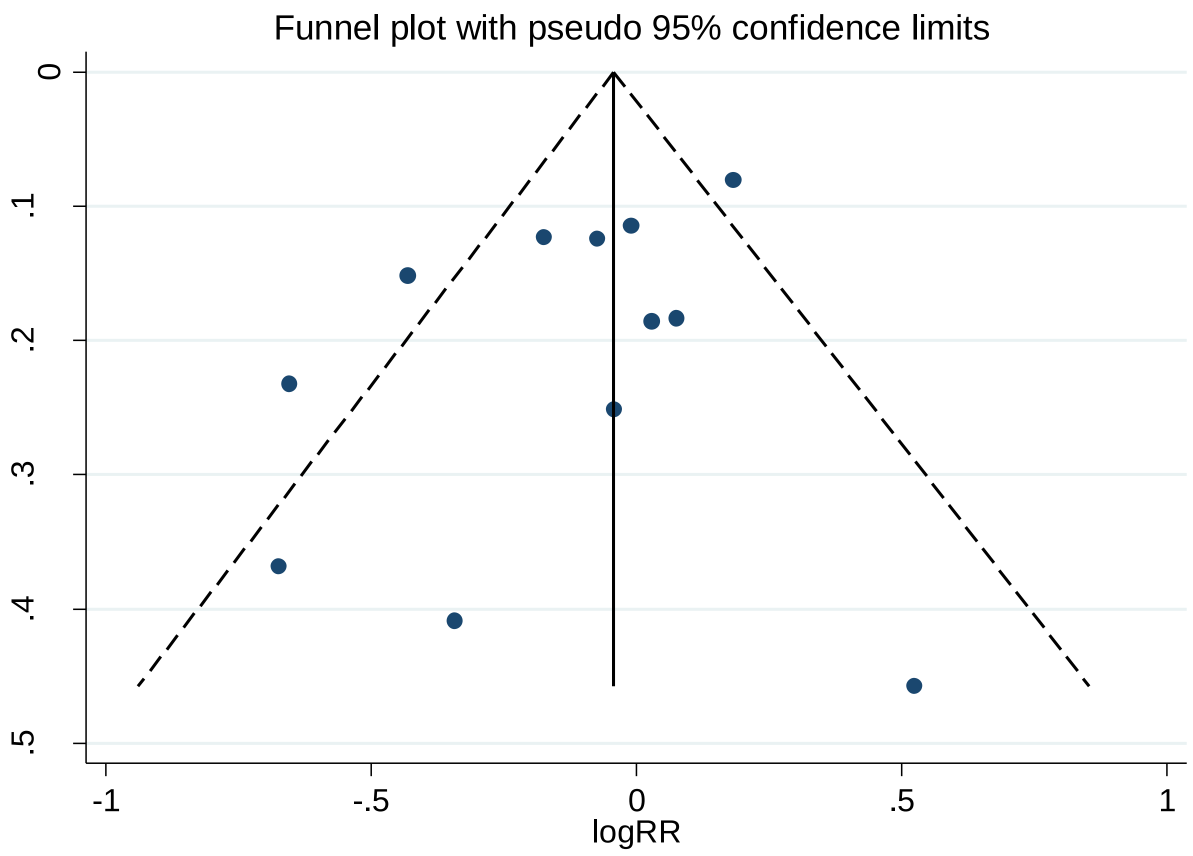

Egger's test: p=0.15

Begg's test: p=0. 0.63

Supplementary Figure 9. Influence analysis of vegetarian vs. nonvegetarian diets and total stroke

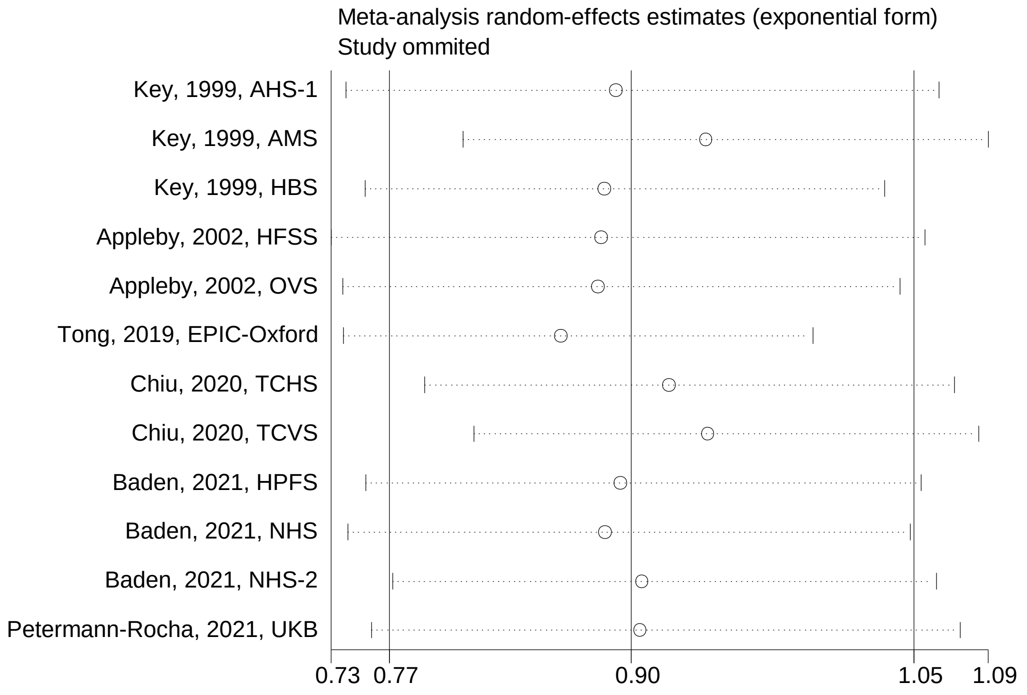

Study omitted	e^coef.	[95% Conf.	Interval]	I^2 (%)
Key, 1999, AHS-1	0.88837582	0.74261391	1.0627482	64
Key, 1999, AMS	0.93694377	0.80576199	1.0894825	62
Key, 1999, HBS	0.88202071	0.75278991	1.0334364	52
Appleby, 2002, HFSS	0.88037497	0.73453367	1.0551729	64
Appleby, 2002, OVS	0.87851447	0.74074394	1.0419087	64
Tong, 2019, EPIC-Oxford	0.85869026	0.74125475	0.9947308	41
Chiu, 2020, TCHS	0.91704398	0.78513765	1.0711112	52
Chiu, 2020, TCVS	0.93799043	0.81151730	1.0841742	63
Petermann-Rocha, 2021, UKB	0.90139925	0.75630683	1.0743268	60
Baden, 2021, HPFS	0.89062762	0.75310785	1.0532589	64
Baden, 2021, NHS1	0.88243562	0.74351639	1.0473107	64
Baden, 2021, NHS2	0.90268236	0.76768732	1.0614158	64
Combined	0.8965236	0.76600396	1.0492825	61

Abbreviations: AHS-1 Adventist Health Study 1; AMS Adventist Mortality Study 2; CBVD cerebrovascular disease; EPIC-Oxford European Prospective Investigation into Cancer and Nutrition - Oxford; HBS Heidelberg Study; HFSS Health Food Shoppers Study; HPFS

Health Professionals Follow-up Study; NHS1 Nurses' Health Study 1; NHS2 Nurses' Health Study 2; OVS Oxford Vegetarian Study; TCHS Tzu Chi Health Study; TCVS Tzu Chi Vegetarian Study; UKB UK Biobank Study

Supplementary Figure 10. Vegetarian vs. nonvegetarian diets and cardiovascular disease, subgroup analysis stratified by incidence or mortality

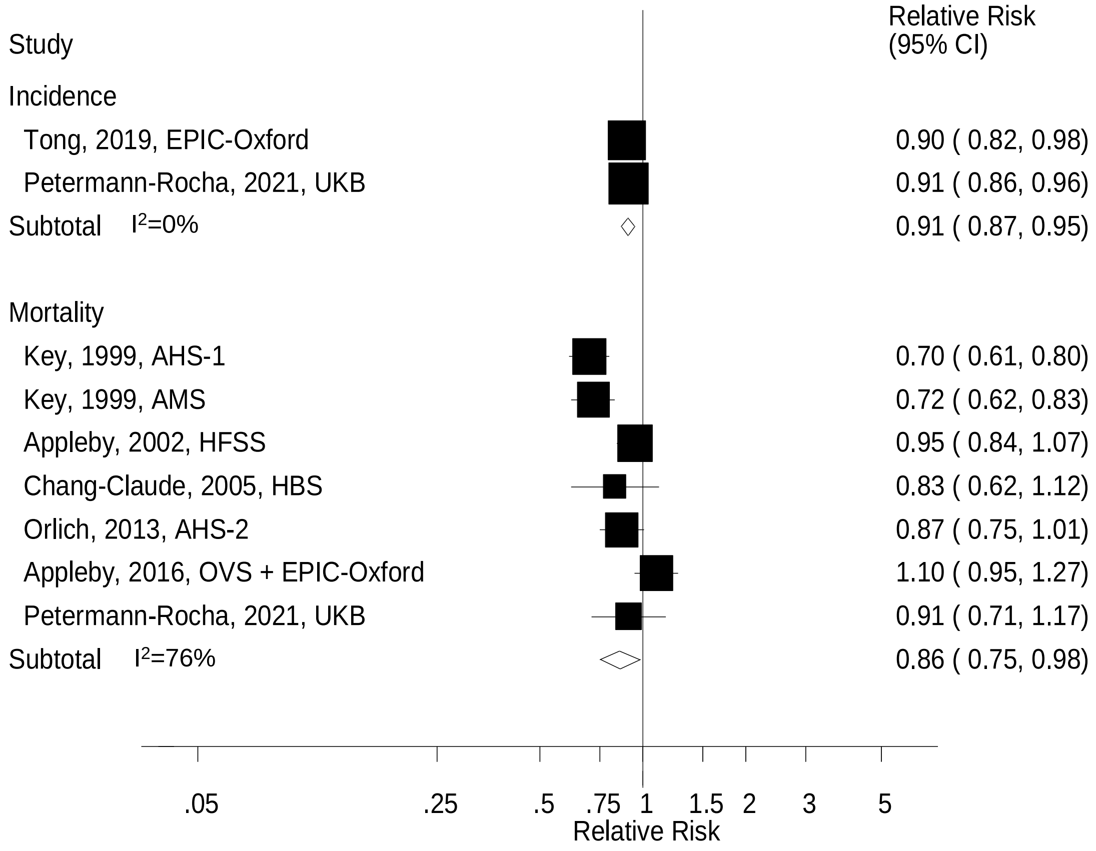

Abbreviations: AHS-1 Adventist Health Study 1; AHS-2 Adventist Health Study 2; AMS Adventist Mortality Study; EPIC-Oxford European Prospective Investigation into Cancer and Nutrition - Oxford; HBS Heidelberg Study; HFSS Health Food Shoppers Study; OVS Oxford Vegetarian Study; UKB UK Biobank Study

Supplementary Figure 11. **Vegetarian vs. nonvegetarian diets and ischemic heart disease, subgroup analysis stratified by incidence or mortality**

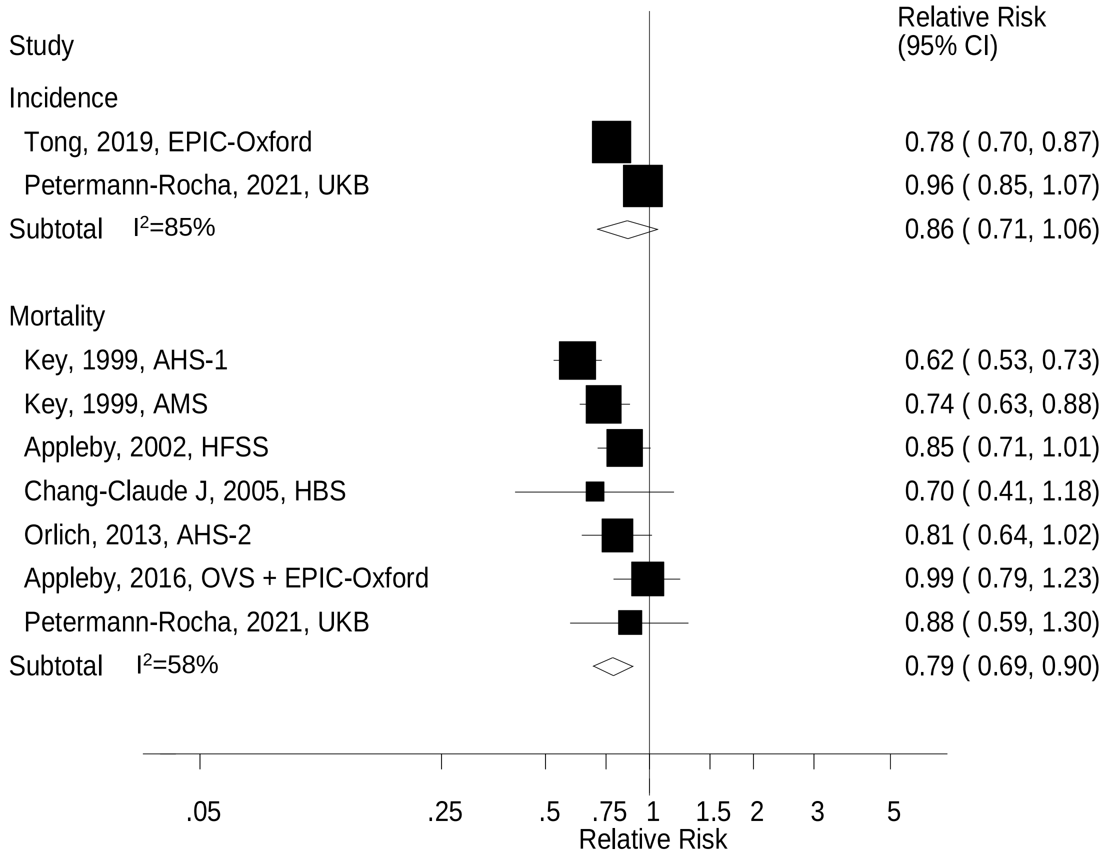

Abbreviations: AHS-1 Adventist Health Study 1; AHS-2 Adventist Health Study 2; AMS Adventist Mortality Study; EPIC-Oxford European Prospective Investigation into Cancer and Nutrition - Oxford; HBS Heidelberg Study; HFSS Health Food Shoppers Study; OVS Oxford Vegetarian Study; UKB UK Biobank Study

Supplementary Figure 12. Vegetarian vs. nonvegetarian diets and total stroke, subgroup analysis stratified by incidence or mortality

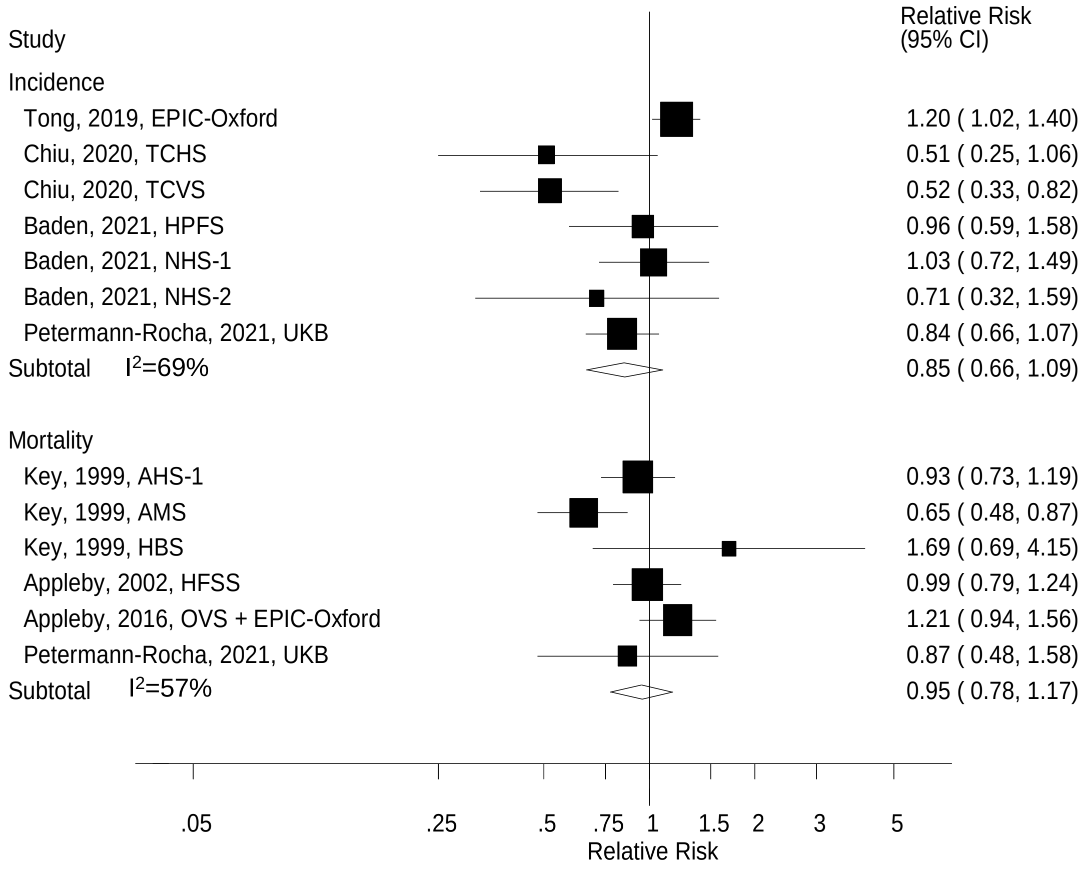

Abbreviations: AHS-1 Adventist Health Study; AMS Adventist Mortality Study; EPIC-Oxford European Prospective Investigation into Cancer and Nutrition - Oxford; HBS Heidelberg Study; HFSS Health Food Shoppers Study; HPFS Health Professionals Follow-up Study; NHS1 Nurses' Health Study 1; NHS2 Nurses' Health Study 2; OVS Oxford Vegetarian Study; TCHS Tzu Chi Health Study; TCVS Tzu Chi Vegetarian Study; UKB UK Biobank Study

Supplementary Figure 13. Vegetarian vs. nonvegetarian diets and cardiovascular disease, subgroup analysis stratified by cardiovascular disease or circulatory disease

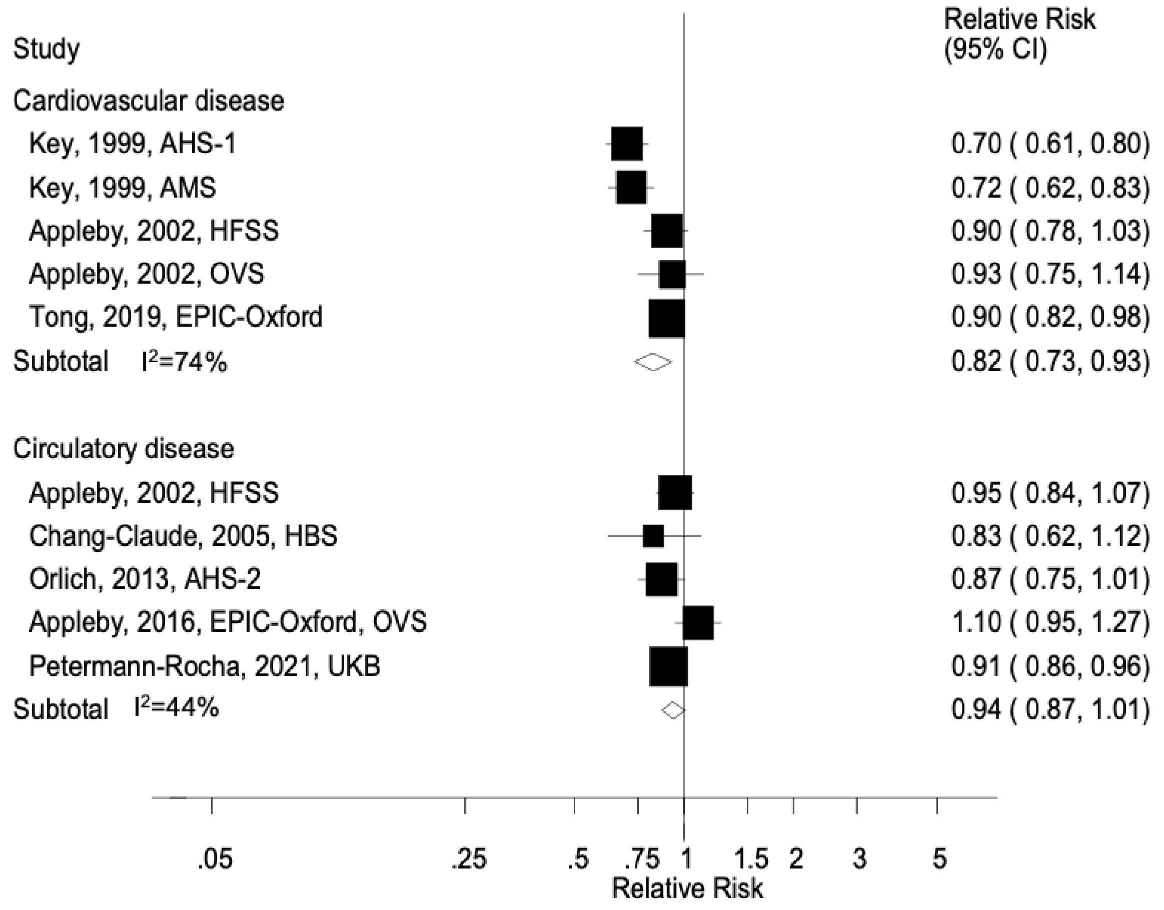

Abbreviations: AHS-1 Adventist Health Study 1; AHS-2 Adventist Health Study 2; AMS Adventist Mortality Study; EPIC-Oxford European Prospective Investigation into Cancer and Nutrition - Oxford; HBS Heidelberg Study; HFSS Health Food Shoppers Study; OVS Oxford Vegetarian Study; UKB UK Biobank Study

Supplementary Figure 14. Vegetarian vs. nonvegetarian diets and total stroke, subgroup analysis stratified by total stroke or cerebrovascular disease

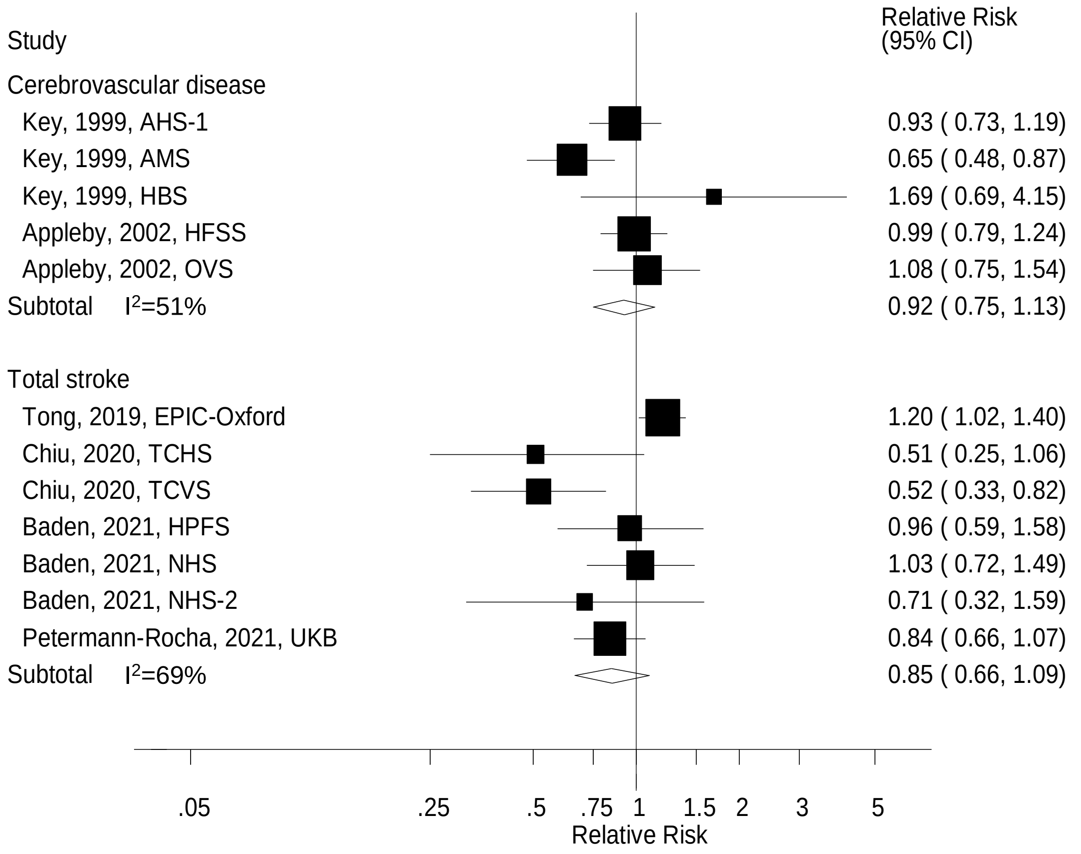

Abbreviations: AHS-1 Adventist Health Study 1; AMS Adventist Mortality Study 2; EPIC-Oxford European Prospective Investigation into Cancer and Nutrition - Oxford; HBS Heidelberg Study; HFSS Health Food Shoppers Study; HPFS Health Professionals Follow-up Study; NHS1 Nurses’ Health Study 1; NHS2 Nurses’ Health Study 2; OVS Oxford Vegetarian Study; TCHS Tzu Chi Health Study; TCVS Tzu Chi Vegetarian Study; UKB UK Biobank Study
